# Distribution and abundance of *Aedes caspius* (Pallas, 1771) and *Aedes vexans* (Meigen, 1830) in the Po Plain (northern Italy)

**DOI:** 10.1186/s13071-024-06527-8

**Published:** 2024-11-05

**Authors:** Mattia Calzolari, Andrea Mosca, Fabrizio Montarsi, Annalisa Grisendi, Mara Scremin, Paolo Roberto, Carlotta Tessarolo, Francesco Defilippo, Federica Gobbo, Cristina Casalone, Davide Lelli, Alessandro Albieri

**Affiliations:** 1 Istituto Zooprofilattico Sperimentale della Lombardia e dell’Emilia Romagna “B. Ubertini”, Brescia, Italy; 2 Istituto per le Piante da Legno e l’Ambiente, Turin, Italy; 3 Istituto Zooprofilattico Sperimentale delle Venezie, Legnaro, Italy; 4 Istituto Zooprofilattico Sperimentale del Piemonte, Liguria e Valle d’Aosta, Turin, Italy; 5 Sanitary Entomology and Zoology Department, Centro Agricoltura Ambiente “G. Nicoli”, Crevalcore, Italy

**Keywords:** *Aedes caspius*, *Aedes vexans*, Entomological surveillance

## Abstract

**Background:**

Knowledge of the distribution and abundance of disease-causing mosquito vectors is fundamental for assessing the risk of disease circulation and introduction. *Aedes caspius* (Pallas, 1771) and *Aedes vexans* (Meigen, 1830) have been implicated, to different extents, in the circulation of several arthropod-borne viruses (arboviruses). These two mosquitoes are vectors of Tahyna virus in Europe and are considered potential vectors of Rift Valley fever virus, a virus not present but at risk of introduction on the continent.

**Methods:**

In this work, we analysed abundance data collected during West Nile virus (WNV) surveillance in northern Italy (Po Plain) via 292 CO_2_-baited traps to evaluate the distribution and density of these two non-target mosquitoes. We modelled the distribution and abundance of these two mosquito species in the surveyed area using two distinct spatial analysis approaches (geostatistical and machine learning), which yielded congruent results.

**Results:**

Both species are more abundant close to the Po River than elsewhere, but *Ae. caspius* is present in the eastern and western parts of the plain, linked with the occurrence of rice fields and wetlands, while *Ae. vexans* is observed in the middle area of the plain.

**Conclusions:**

Presence and abundance data at the municipality level were obtained and made available through this work. This work demonstrates the importance of maintaining and improving entomological surveillance programs with an adequate sampling effort.

**Graphical Abstract:**

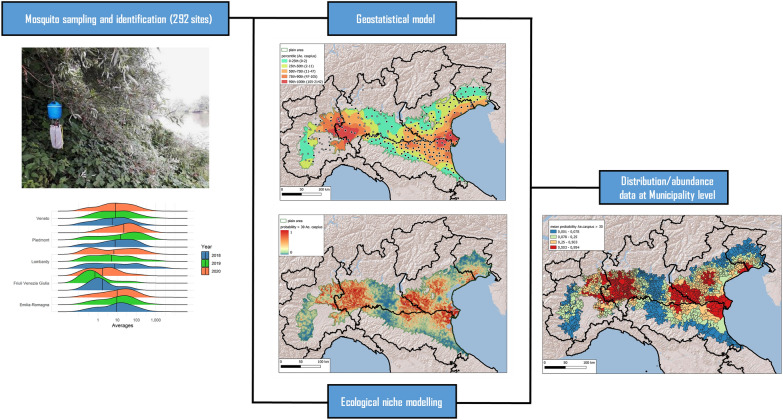

**Supplementary Information:**

The online version contains supplementary material available at 10.1186/s13071-024-06527-8.

## Background

The transmission of arthropod-borne diseases is strictly linked to the presence of a competent vector in a given area. Vector competence is a biological characteristic of arthropod vectors and is characterised by the intrinsic ability to infection, replication and transmission of a vertebrate pathogen [1].

*Aedes caspius* (Pallas, 1771) and *Aedes vexans* (Meigen, 1830) are competent vectors of several vertebrate pathogens. In Europe, both species are involved in the transmission of Tahyna virus (TAHV) [2–6]. TAHV is a mosquito-borne virus that may cause mild flu-like symptoms or neurological symptoms in humans, while hares, rabbits, hedgehogs, and rodents serve as amplifying hosts. The more relevant disease potentially transmitted by these two species is Rift Valley fever (RVF), a mosquito-borne zoonosis affecting mainly humans and ruminants that severely impacts public health and economic losses, especially in Africa [7, 8], and experimental studies have confirmed that this mosquito and *Ae. caspius* are competent vectors [9].

*Aedes vexans* and *Ae. caspius* were suspected to be involved in the transmission of several other pathogens to different extents; among them some are circulating in Europe, such as West Nile virus (WNV), and other are at risk of introduction as Zika virus and Japanese encephalitis virus [10–16]. *Aedes vexans* and *Ae. caspius* were also recorded as potential vectors of *Dirofilaria immitis* [17] and *Dirofilaria repens* [18, 19].

In addition to the ability to transmit various pathogens, these mosquitoes can constitute a source of great nuisance; both species exhibit an aggressive behaviour and readily feed on humans and domestic animals. Moreover, the two mosquitoes are strong flyers that can reach areas very far from their breeding sites with maximum distances exceeding 15 km for *Ae. vexans* [20] and 20 km for *Ae. caspius* [21]. Both are polycyclic mosquitoes and overwinter at the egg stage [20]. The earliest detection of *Ae. caspius* occurred from February to March in southern Europe, while *Ae. vexans* is a typical summer species, with temperatures above 30 °C [22].

The Po Plain, the largest plain in Italy, represents a suitable environment for both species and for the circulation of different arboviruses, such as TAHV, WNV and Usutu virus (USUV) [23–25]. In this study, we estimate the distribution and abundance of *Ae. caspius* and *Ae. vexans* using data collected between 2018 and 2020 via a network of traps activated for the WNV National Surveillance Plan [26]. The distribution and density of *Ae. caspius* and *Ae. vexans* in the Po Plain were estimated by a quantitative geostatistical analysis-based approach using geographic information system (GIS) analysis. The suitability of the surveyed area for high abundance of the two mosquitoes was evaluated by a qualitative machine learning-based approach [27].

## Methods

### Surveyed area

The Po Plain is a continuous plain of ~ 46,000 km^2^ [28], which is crossed by the Po River and other rivers. It is the largest Italian plain and one of the major regions of southern Europe and provides an environment largely suitable for many mosquitoes.

The Po Plain hosts more than 20 million inhabitants and is one of the most densely populated areas of Italy; it covers part of the territories of five Italian regions (Nomenclature of Territorial Units for Statistics of Level 2, NUTS 2): Piedmont, Lombardy, Emilia-Romagna, Veneto and Friuli-Venezia Giulia. This territory is geared towards agriculture, characterised by intensive farming and animal husbandry, with few hedges, rare scattered trees and a dense irrigation network. Industrial settlements and residential areas often intertwine within this agricultural environment. Natural areas are rare and mainly represented by river borders characterised by riparian vegetation or protected and rewilding areas.

The climate is continental temperate and Mediterranean towards the coast; it is characterised by severe summers and precipitation values between 600 and 1000 mm per year, which is evenly distributed across the various seasons, with a slight prevalence in spring and autumn [29].

### Mosquito sampling

Mosquitoes were collected in the period 2018–2020 as part of the WNV surveillance implemented at the regional level. The regional surveillance systems were based on the same model of attractive traps and adopted the same periodicity of sampling in summer. Mosquitoes were collected by CDC-like traps baited with approximately one kilogram of dry ice pellets placed in a bottom-drilled thermos. The traps operated overnight, from approximately 17:00 to 9:00 the next day, every 2 weeks. The sampling period of the WNV surveillance was from May to October.

**Fig. 1:**
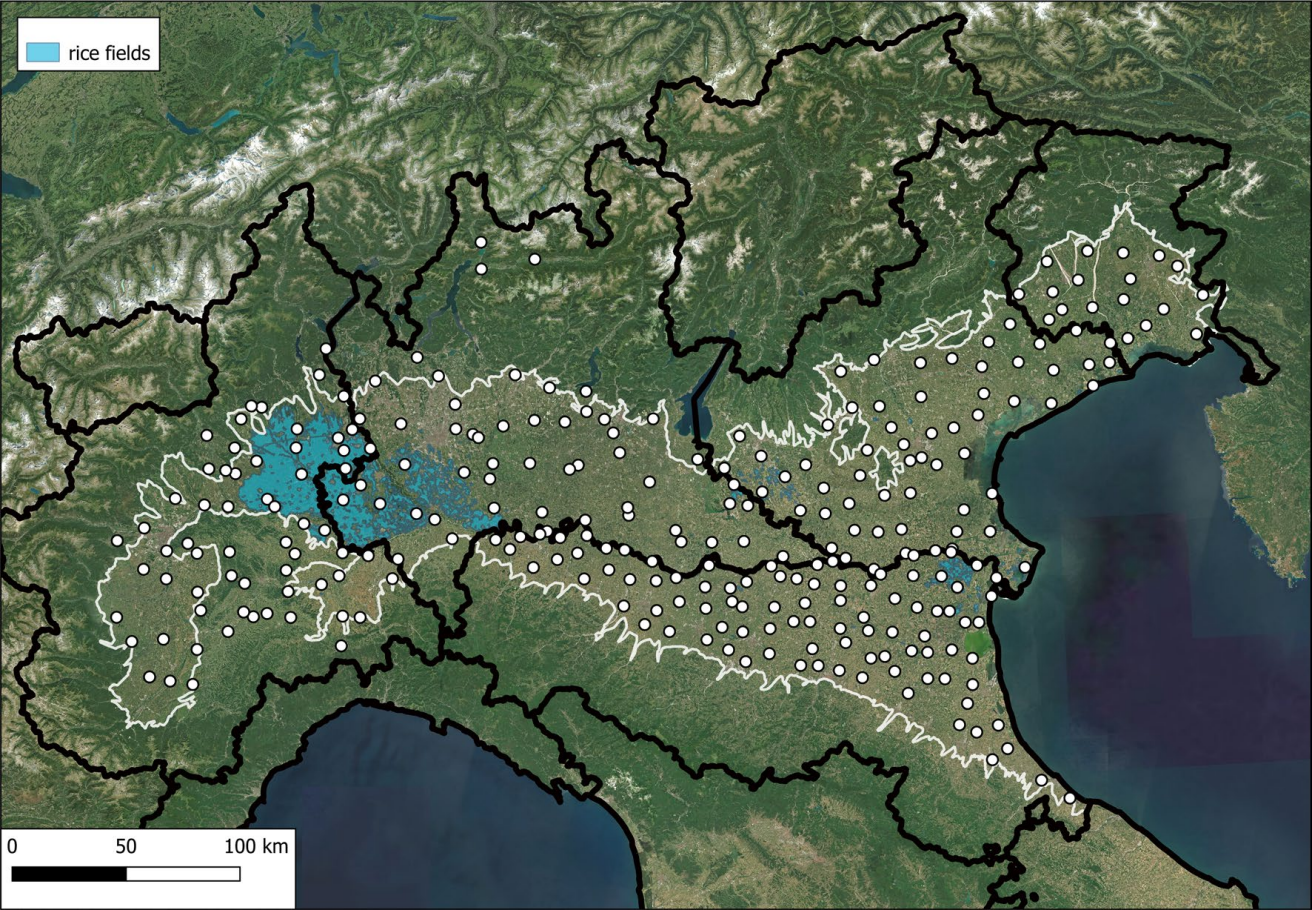
Sample station locations in the Po Plain (delimited by a white line) with reference to the monitored regions and the Italian map

A total of 292 CO_2_-baited traps were placed across the entire studied area. The trap locations were georeferenced and their distribution is shown in Fig. 1.

### Identification and spatial analysis

The collected mosquitoes were killed by freezing before they were identified at the species level according to morphological keys [20, 30]. The traditional classification of mosquitoes was adopted in this work, as proposed by Savage and Strickman [31], *Ochlerotatus* was considered an *Aedes* subgenus.

Since the statistics data indicated non-normal distribution to be normalised, the data were then transformed using the log_10_ function of the average for 2018–2020 (Supplementary Material, Fig. S1, S2). Thus, the traps with zero data for all three years (i.e., 224 sites for *Ae. caspius* and 205 sites for *Ae. vexans*) were removed, and the data were normalised (Supplementary Material Tab S2).

### Geostatistical model

The datasets of the two species comprised the mean between 2018 and 2020 of 223 traps for *Ae. caspius* and 205 traps for *Ae. vexans* (traps with at least one female and a minimum number of 11 observations were used, to exclude occasionally sampled sites). The obtained values were log_10_ transformed to get the required normality of data.

The autocorrelation between the sampling points, based on mosquito abundance, was calculated by the global Moran’s I at multiple distances and measured by Getis Ord Gi. This analysis assessed the existence of hot spots as statistically significant clusters.

As a good compromise between the results of Moran’s I multiple distance calculation and the suggestion of the software for error reduction, a search radius of 40 km was chosen for the ordinary kriging interpolation. The ordinary kriging was performed after the optimal variogram calculations for the two species and interpolated maps were created. The goodness of fit of the variogram models was evaluated by spatial structure contribution criteria and the coefficient of determination (R2). Cross-validation was employed for analysing the estimates using the root mean square error (RMSE) [32, 33]. A stronger spatial structure and a larger R2 value represent a better variogram model [34]. To evaluate the estimates, the smaller the criterion is, the better and the more accurate the estimates.

Statistical data analysis was performed in Jamovi v2, and geostatistical analysis was performed using QGIS 3.22 and Python plugins (Smart-Map and HotSpot Analysis). All the used applications are open-source applications.

### Ecological niche modelling

Maxent software version 3.4.1 [27] is an application for species distribution modelling (SDM) that provides a suitability model across a grid, based on a list of presence points and a set of environmental predictors.

To identify areas with a potential risk of mosquito nuisance, we used Maxent traps with an average number of females greater than a specific threshold as presence points. A limit of 30 mosquitoes per trap per night, proposed as a nuisance threshold in northern Italy [35], was used for *Ae. caspius*; an arbitrary threshold of 15 mosquitoes/trap was used for *Ae. vexans*: this value corresponds to the mean of *Ae. vexans* specimens at sites where this species was observed in this work.

The parameter settings used in our analyses were calculated through the ENMeval package in R 4.1.2 (https://cran.r-project.org/web/packages/ENMeval/index.html). The background was created using 10,000 random points automatically generated by Maxent. Duplicate presence records per cell were removed, and the output grid format was set to complementary log–log model (Cloglog). The minimum training threshold was adopted to convert maps from suitability indices to presence/absence indices (GPS data).

In the model, a bias raster was used to account for the different monitoring efforts in each region, creating a raster with the number of activated traps within 100 km^2^.

The contribution of the predictor variables was assessed by jackknife analysis in the Maxent model to obtain alternate estimates of which variables were most important in the model [36]. All variables with a contribution less than 1% were excluded from the final analysis. To assess the model accuracy, we used tenfold cross validation for *Ae. caspius* and five-fold cross validation for *Ae. vexans* (fewer than 50 observations), and we calculated the mean area under the curve (AUC).

The dataset of covariates (Table S1) was identical to that used to generate the distribution models of *Anopheles maculipennis* s.l. in the same area of study [37].

## Results

Notably, the total number of female mosquitoes collected over 3 years was 319,331 for *Ae. caspius* and 88,153 for *Ae. vexans* (Table 1).

**Table 1:** Total number of mosquitoes collected per year from 2018 to 2020 in the five regions of northern Italy

Region	Traps	*Aedes caspius*				*Aedes vexans*			
		2018	2019	2020	Total	2018	2019	2020	Total
Emilia-Romagna	95	41,875	37,404	57,509	136,788	6858	35,511	36,378	78,747
Friuli Venezia Giulia	18	654	794	1275	2723	18	178	306	502
Lombardy	52	46,149	14,419	9583	70,151	804	1091	1982	3877
Piedmont	68	7248	25,595	21,062	53,905	702	512	286	1500
Veneto	59	12,343	24,498	18,923	55,764	544	1825	1158	3527
Total	292	108,269	102,710	108,352	319,331	8926	39,117	40,110	88,153

**Fig. 2:**
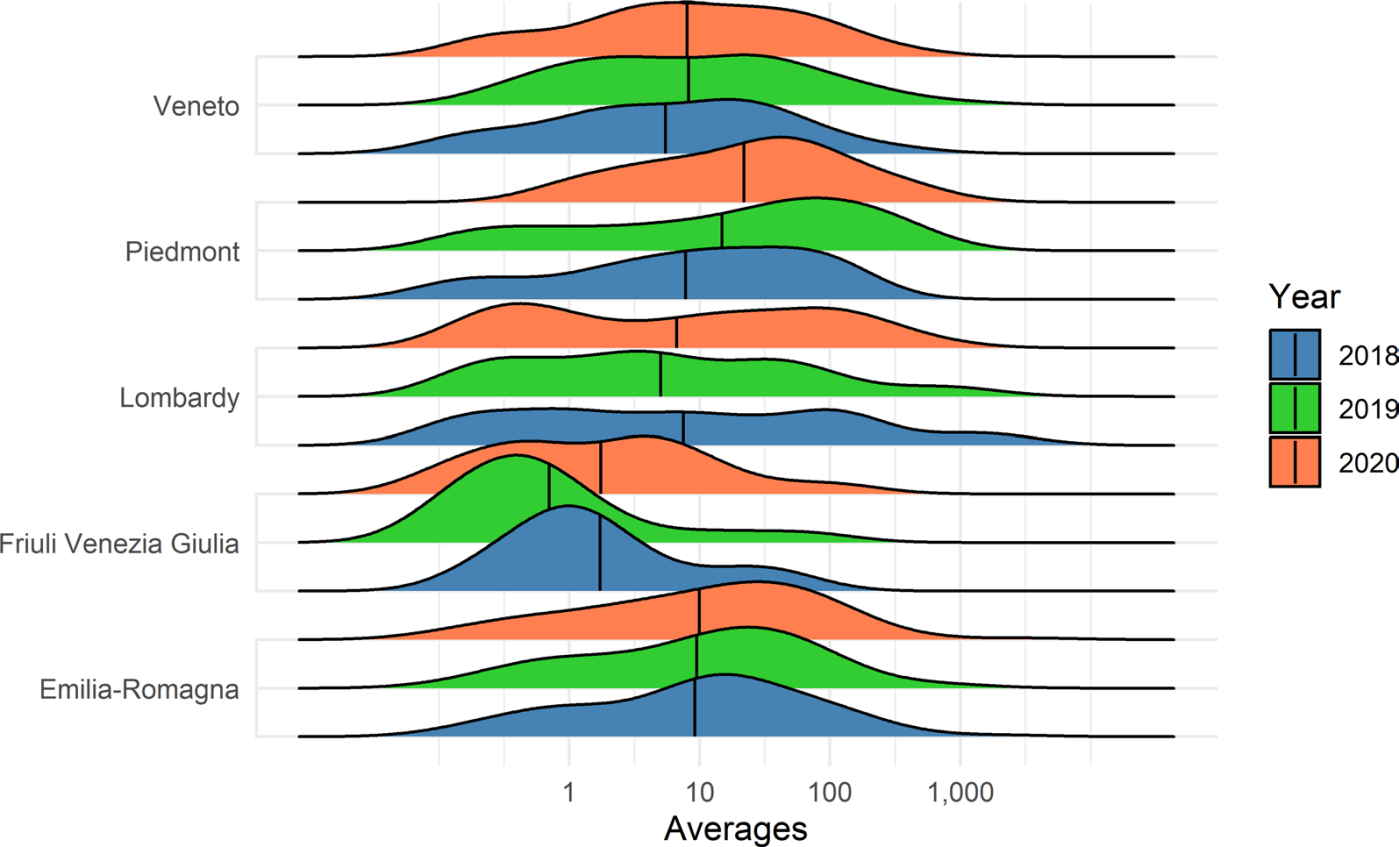
*Aedes caspius*: yearly average collection values for the five regions of northern Italy on a logarithmic scale and reference to the seasonal average (black line)

**Fig. 3:**
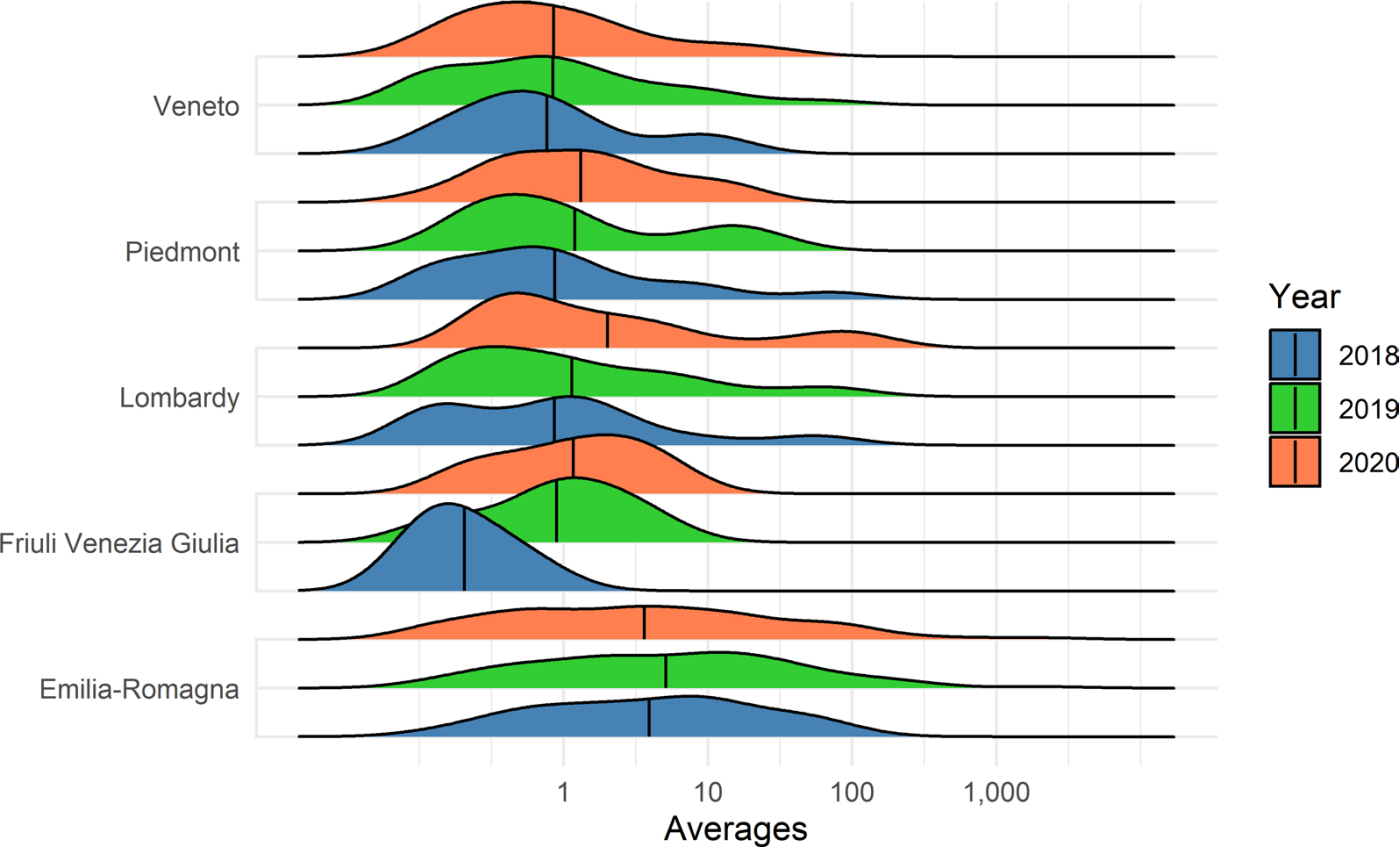
*Aedes vexans*: yearly average collection values for the five regions of northern Italy on a logarithmic scale and reference to the seasonal average (black line)

The mean number of mosquitoes collected per trap differed between years and regions (Figs. 2 and 3, respectively). Univocal trends for all the regions (abundance peak of mosquitoes in the same year) were not evident between years. Emilia-Romagna shows the most consistent pattern between years for both *Ae. caspius* and *Ae. vexans*; however, Friuli Venezia Giulia was the most dissimilar region.

The average number of *Ae. vexans* collected in Emilia-Romagna was significantly larger (Fig. 2) than that collected in the other regions (*F*_3,193_ = 11.2; *P* < 0.001), while no meaningful difference was recorded for *Ae. caspius*. High variability between seasons was recorded in the different regions.

### Geostatistical analysis results

The data for both species demonstrated statistically significant positive spatial autocorrelation, which peaked at 30 km for both species (*Ae. caspius,* Moran’s I: 0.51; *Ae. vexans,* Moran’s I: 0.21).

**Fig. 4:**
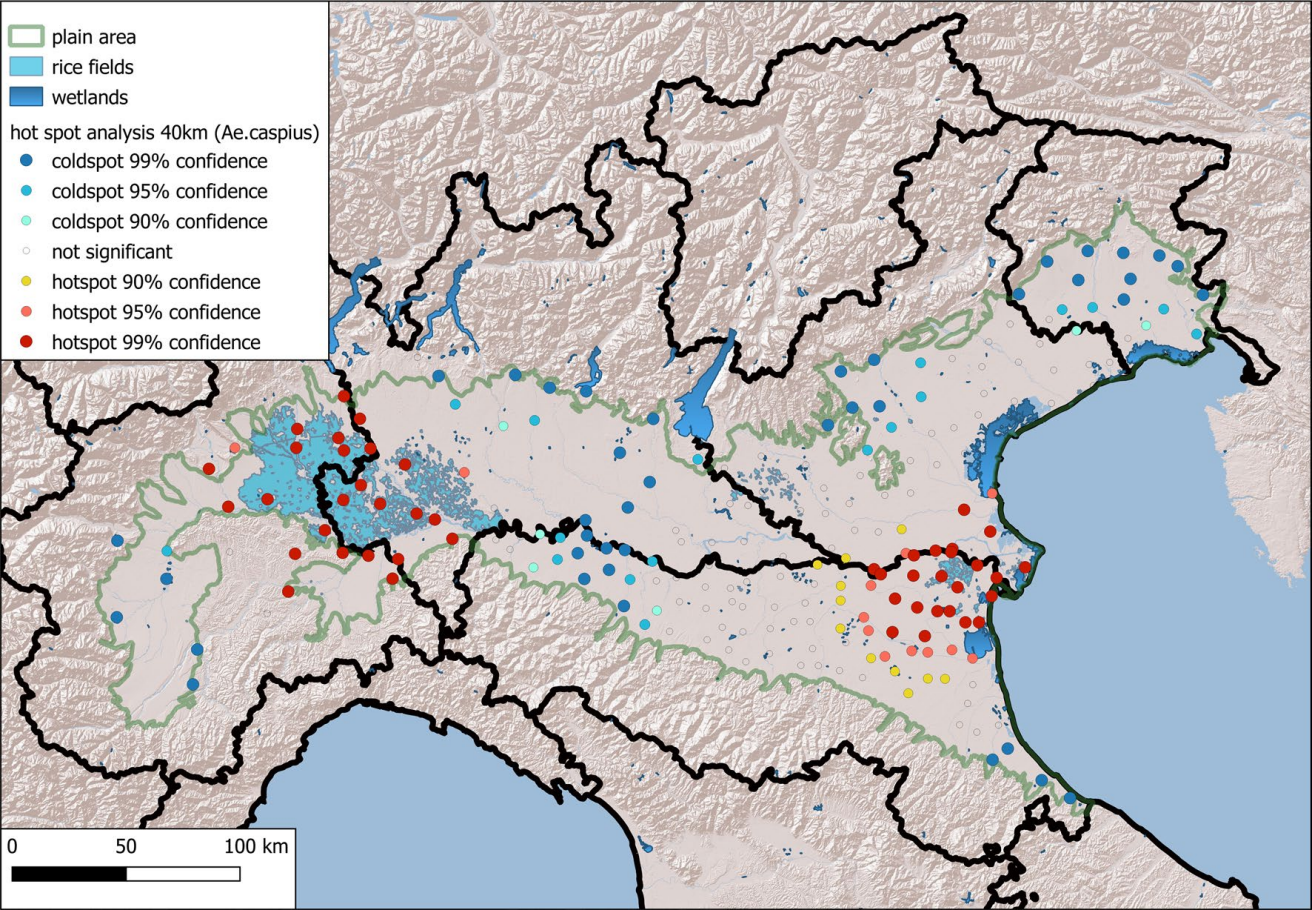
Maps of the hotspots derived from Getis Ord analysis of the local autocorrelation of *Aedes caspius*

**Fig. 5:**
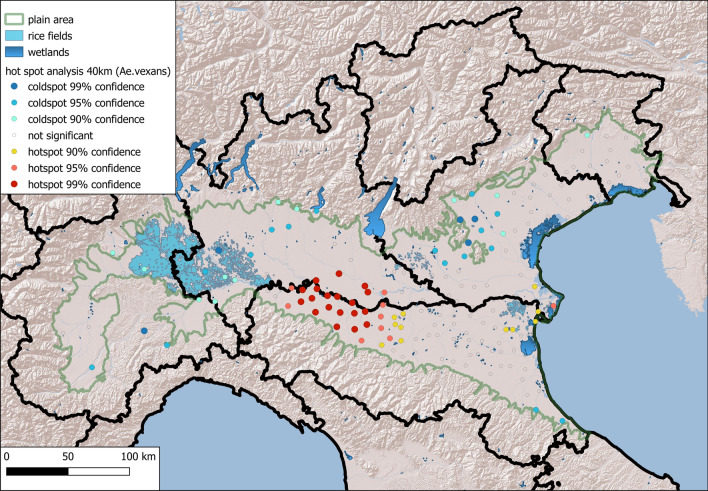
Map of the hotspots derived from Getis Ord analysis of the local autocorrelation of *Aedes vexans*

Getis Ord Gi revealed significant hot spots of *Ae. caspius* coincident with wide areas of rice fields (Fig. 4) in the western Po Plain (between Lombardy and Piedmont) and in the eastern part, near the Adriatic coast, where rice fields and wetlands occur (mean number of females/night: 173). For *Ae. vexans,* a significant hotspot was observed along the Po River in the middle of the study area, and a less significant (90% confidence) hotspot was observed in the eastern part of the Po Plain (Fig. 5) (mean number of females/night: 77).

**Fig. 6:**
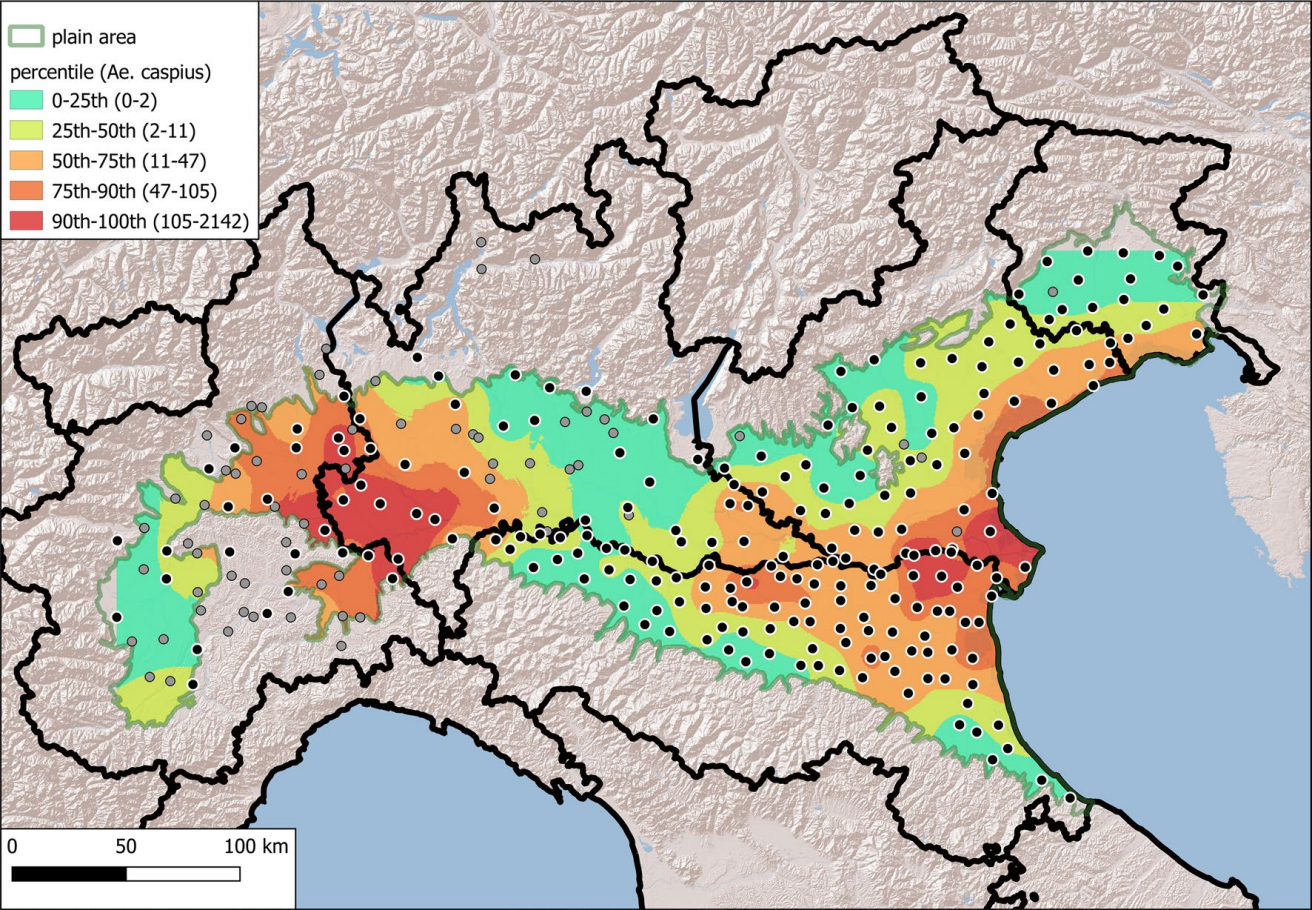
Ordinary kriging interpolation maps of the mean distribution density (log-transformed) of *Aedes caspius* in the three surveillance years (2018, 2019 and 2020); the black dots denote the traps considered in each analysis, in grey traps not considered

**Fig. 7:**
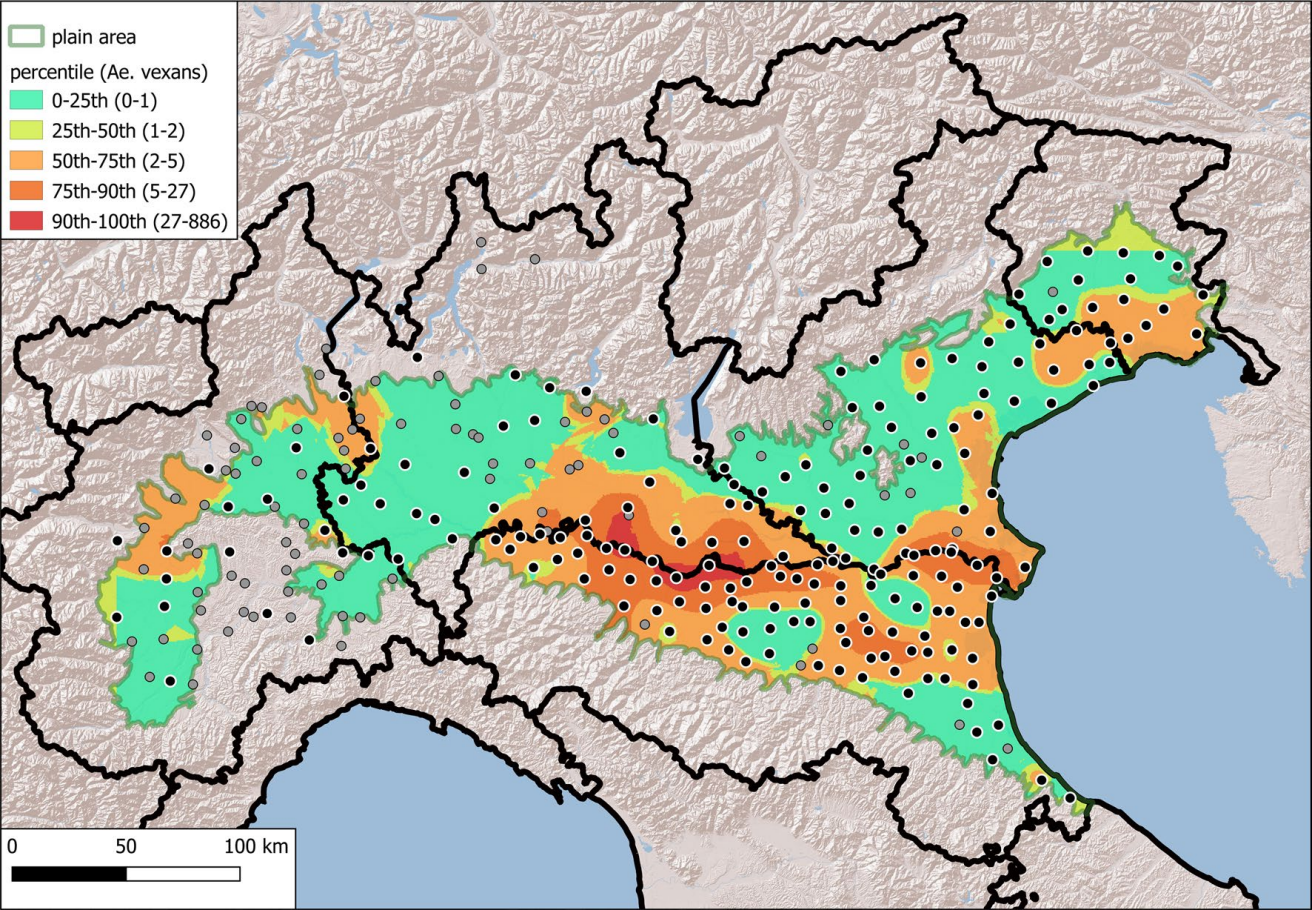
Ordinary kriging interpolation maps of the mean distribution density (log-transformed) of *Aedes vexans* in the three surveillance years (2018, 2019 and 2020); the black dots denote the traps considered in each analysis, in grey traps not considered

The semivariograms created by ordinary kriging showed spatial dependence (range) within approximately 40 km for both species (Figs. 6 and 7), beyond which the semivariance remained constant. The best-fitting model for both species was the spherical model, and summary data are provided in Table 2.

**Table 2:** Parameters related to the variograms of the models and evaluation criteria for the estimates

Species	Model	Model parameters				Prediction errors	
		C/C + C0	C + C0	C0	RMSE	R2	Range
*Aedes caspius*	Spherical	0.881356	0.708	0.084	0.611	0.61	58,784
*Aedes vexans*	Spherical	0.627673	0.795	0.296	0.793	0.21	28,739

Because the consideration of C0 (the nugget effect) alone could be misleading, the proportion of the spatial structure of the data, i.e., the ratio of the scale (C) to the sill (C + C0), is typically employed. The closer the value is to one, the stronger the spatial structure of the data in that model. Values greater than 0.75, 0.25 to 0.75 and less than 0.25 indicate strong, moderate, and weak spatial structures, respectively [38].

Raster maps derived from ordinary kriging interpolation of log-transformed average densities of the two species from 2018 to 2020 were created at a 500 m resolution and a search radius of 40 km (Figs. 6 and 7). The areas with the highest densities of the two species overlapped with the hotspots obtained in the previous analysis.

The average of the obtained abundance interpolated within the area of every municipality (local administrative units, LAUs) in the Po Plain was categorised according to quartiles as low, medium low, medium high, or high. These data are listed in Table S3 and shown in choropleth maps in Figs. S5–S8.

### Results of the Ecological Niche Model (Maxent)

Maxent was used to identify areas with high densities of *Ae. caspius* and *Ae. vexans*. We included 73 traps that exceeded a three-year mean density 30, as the nuisance threshold for *Ae. caspius*, and 34 traps with 3-year mean density greater than 15 for *Ae. vexans*. The relative contributions to the Maxent models are provided in Table 3.

**Table 3:** Relative contributions to the Maxent models of the selected covariates

Covariate	*Aedes caspius* % contribution	*Aedes vexans* % contribution
Proximity of rice fields	65.5	
Soil^a^	1.8	26.7
Corine land cover 2018	6	21.4
Slope		12.9
Proximity of water bodies < 1 km^2^	10.8	9.6
Altitude	0	10.5
Middle infrared^b^		6.3
Enhanced vegetation index	5.5	0
Middle infrared^c^	0.3	5.1
Proximity of rivers	0.3	3.9
Proximity of wetlands	3.8	1.9
Daytime land surface temperature^d^	3.3	

^a^USDA classification

^b^Amplitude of the annual cycle

^c^Phase of the tri-annual cycle

^d^Proportion of the total variance due to the tri-annual cycle

Covariates with greater than 10% contributions to the *Ae. caspius* model were directly related to the proximity of rice fields and small water bodies (< 1 km^2^), such as disused quarries, artificial lakes, re-naturalized areas, fish ponds, irrigation reservoirs and settling basins. The most important explanatory variables for *Ae. vexans* were indicated the presence of water bodies, either directly or indirectly, considered from different approaches (land use, soil type, presence of water bodies): the soil category Aquents (according to the USDA classification), the Corine land cover categories of inland marshes (4.1.1) and water courses (5.1.1), low altitude and slope, and proximity to small water bodies (Table 3, Supplementary Material, Fig. S3).

**Fig. 8:**
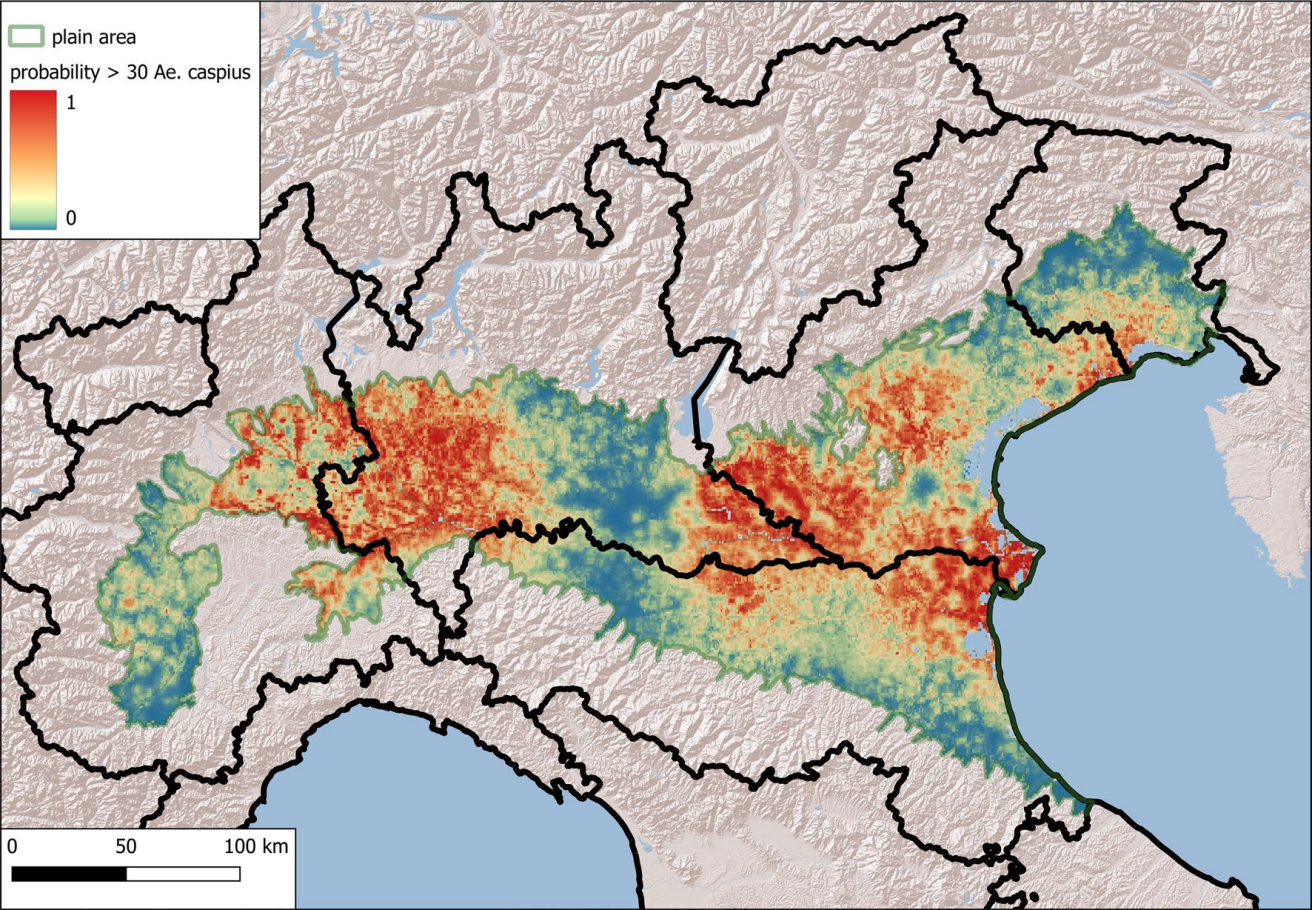
Maxent map of high-density *Aedes caspius* suitability areas (density > 30 females/trap)

The most suitable habitats for *Ae. caspius* were located in the eastern and western parts of the study area. These data agree with the results of other methods, although there are also suitable areas in the middle of the Po Plain (in the provinces of Modena, Reggio-Emilia, and Mantua) (Fig. 8)

**Fig. 9:**
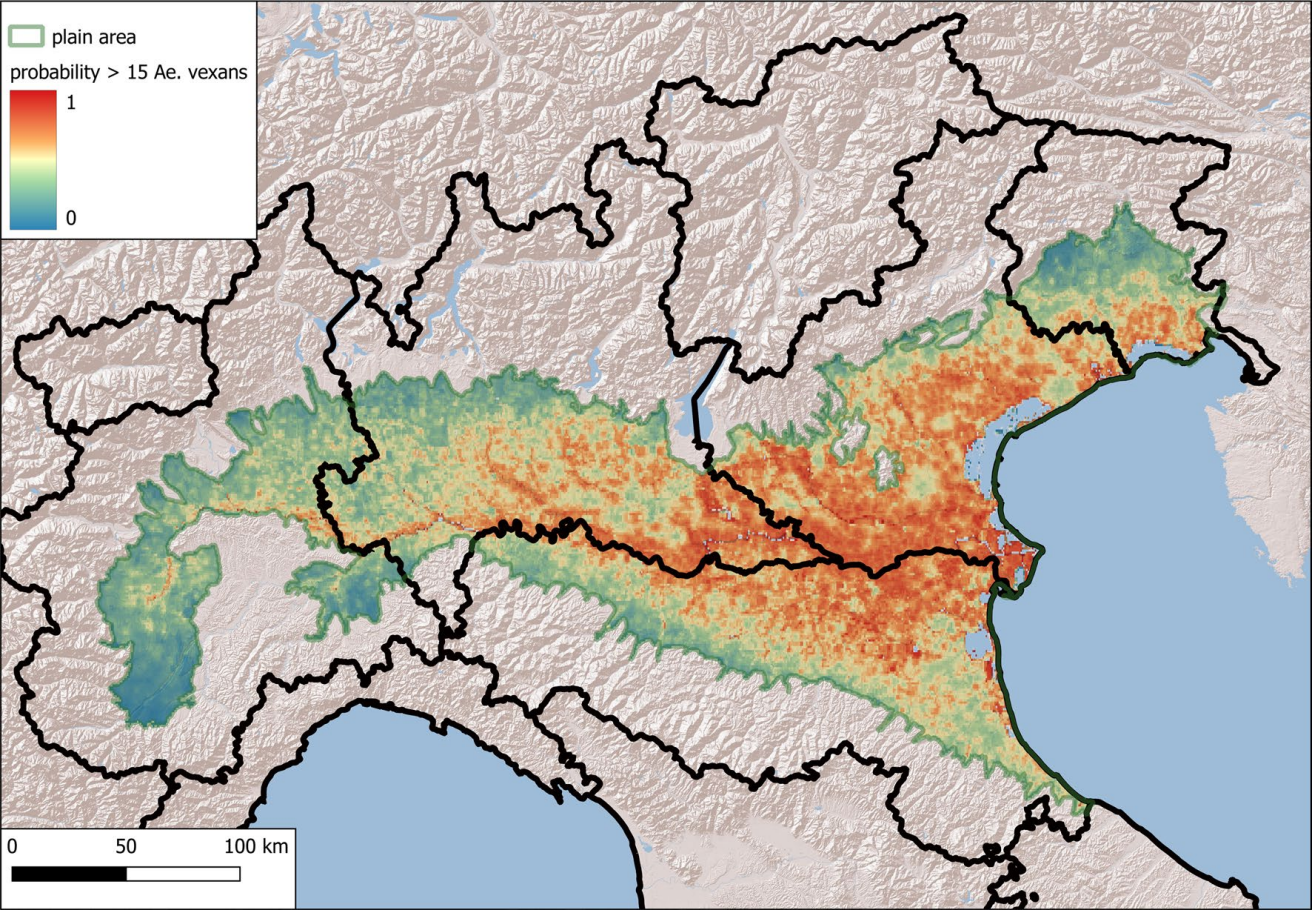
Maxent map of high-density *Aedes vexans* suitability areas (density > 15 females/trap)

The most suitable habitats for *Ae. vexans* are located in the middle of the Po Valley (as demonstrated by other methods) and near the Venice Lagoon (Fig. 9).

The calculated AUC values were 0.80 ± 0.06 for *Ae. caspius* and 0.74 ± 0.06 for *Ae. vexans*.

The jackknife test results for variable importance showed that the environmental variables with the greatest increase in the predictive power of the model when used in isolation were the proximity to rice fields and precipitation during the wettest quarter of the year for *Ae. caspius* and altitude and precipitation during the wettest quarter of the year for *Ae. vexans* (Supplementary Material, Fig. S4).

## Discussion

In this work, we used abundance data collected during WNV surveillance to evaluate the distribution and abundance of *Ae. caspius* and *Ae. vexans*. The WNV surveillance campaign generally targets *Culex pipiens*; however, we obtained data to implement models for *Ae. caspius* and *Ae. vexans*, demonstrating the utility of maintaining and improving such types of surveillance, with an adequate sampling effort.

The utilised data are the result of a wide sampling effort that entails the use of ~ 300 sampling traps, producing accurate and precise data. The two mosquito species showed abundance variability between seasons and even within the same season between the different regions because their abundance is primarily linked to water level fluctuations [30]. The variable abundance of these mosquitoes is strictly linked to precipitation events and artificial flooding in agriculture [20]. We overcame this variability using data from different seasons. Moreover, the samplings were performed in the same area for which the models were developed, avoiding the use of data for estimating abundances in areas without samplings, an approach that could generate incorrect results. We conducted spatial analysis and model creation within an open-source framework, offering a cost-free and widely accessible method to elaborate the obtained data.

We applied two distinct methods to our datasets: a geostatistical model based on the interpolation of the abundance data and a machine learning model based on the relationship between the available environmental variables and presence/absence data. The two models are based on different approaches and provide two types of information: one produces an estimation of abundance, and the other highlights areas suitable for each species. The geostatistical and machine learning models for *Ae. caspius* are very accurate, partly due to the greater availability of data for this species, which was sampled in large numbers and in more traps than *Ae. vexans*. Additionally, the autocorrelation of the *Ae. caspius* data was greater than that of the *Ae. vexans* data, perhaps due to the close link of *Ae. caspius* to wide and homogeneous environments (such as rice fields) with respect to *Ae. vexans*, which breed in more dispersed environments (such as a few semi-permanent water basins), many of which are subjected to periodic larvicidal interventions.

According to the obtained model, both species are widely distributed in the Po Plain, and both models showed that *Ae. caspius* was more abundant in the eastern and western parts, while *Ae. vexans* occurred more diffusely at the centre of the surveyed area. The implemented models were used to obtain abundance and suitability values at the municipality level (LAU) for the two target mosquitoes. These data are useful for assessing the risk related to possible pathogens that can potentially be transmitted by one of the two mosquito species or both. Although the modelled abundance and suitability are largely congruent, some areas are characterised by high suitability and low abundance. This could be due to human interventions (e.g., insecticide treatment) or to model artefacts/sampling bias (small number or non-representative sampling sites). Further sampling efforts could be made in these areas to better characterise the distributions of these two mosquito species. This supports the added value of using different approaches to finely characterise the distribution of mosquitoes.

The two species exhibit common characteristics and often coexist at the same breeding site [39], but their ecology is not completely superimposable; for example, *Ae. caspius* is more resistant to salinity [30] but also flourishes in rice fields, in contrast to *Ae. vexans* [40].

The *Ae. caspius* suitability model is greatly influenced by rice field proximity. Notably, it is a typical mosquito of temporary rainwater accumulations in depressions within wooded areas. This mosquito readily breeds in rice fields, inland marshes, snowmelt, river floods or coastal marshes subjected to intermittent flooding and, usually with little vegetation and a muddy bottom, often with a high concentration of salt [20, 30]. The tolerance to salinity allows *Ae. caspius* to reach relevant abundance in the coastal part of the Po Plain. Parameters that influence the choice of breeding site for this mosquito include the texture of the soil and chemical composition: clay–silt texture, soil humidity and ferric oxide presence favour egg laying. This condition allows the maintenance of humidity and anoxia [41], which are indispensable for egg hatching and larval development [42].

The occurrence of *Ae. vexans* is linked to the altitude and slope of the soil, to the proximity of inland marshes and watercourses, and to the Aquent category of the USDA classification—a typical wet soil—all of which are characteristics of lands subject to waterlogging. All these characteristics agree with the preference of this mosquito for transient waterbodies [20] and with the oviposition behaviour of *Ae. vexans*, which lay eggs individually at sites subjected to flooding by rainwater, overflow, seepage or tidal water [43, 44].

*Aedes vexans* and *Ae. caspius* can be relevant vectors of different diseases. The more relevant disease potentially transmitted by these two species is Rift Valley fever (RVF), and an experimental study confirmed the vector status of the European population of *Ae. vexans* [45]. *Aedes caspius* is rare in Africa and has never been found to be positive for RVF in the field; thus, it could be a potentially competent vector, as its infection rate has been shown to be high in experimental studies [46]. RVF has never been reported in Europe, but it is increasingly expanding in northern Africa and the Middle East [47]. However, its introduction in Europe is considered unlikely, although it is possible that the virus can be established, particularly with infected mosquitoes introduced by aerial transportation [47]. Tahyna virus (THAV) is present in Europe and vector competence of *Ae. caspius* and *Ae. vexans* was demonstrated by experimental transmission [48, 49]. While human cases of the disease are unreported in the surveyed area, the virus was widely detected in mosquitoes, especially in *Ae. caspius*, which is suspected to be the principal vector in the surveyed area [50].

High densities of mosquitoes, particularly of aggressive species with high mobility, such as *Ae. caspius* and *Ae. vexans*, pose a significant challenge, particularly in urban and touristic areas. The management of the impact of these mosquitoes is not easy and requires an understanding of the human annoyance threshold, namely the maximum number of bites that most community members find tolerable [35]. Various factors contribute to this perception, extending beyond the mosquito density, which typically increases from urban to rural areas and the specific mosquito species. Socioeconomic and psychological factors play a crucial role in determining the level of nuisance experienced by the population. Understanding these multifaceted influences is essential for developing effective strategies to mitigate the impact of mosquito invasions on urban communities.

The approaches can be different or improved if they are integrated. Ideally, preventing the formation of breeding sites (source reduction) is the most effective approach because this solves the problem permanently and primarily. However, in some cases, this is very difficult. Prevention in the case of floodwater mosquitoes, such as *Ae. caspius* and *Ae. vexans*, can be primarily achieved in two ways: by eliminating the causes that determine water accumulation or by preventing variations in water levels that trigger egg deposition and hatching cycles. Examples of the first approach are the complete drainage of marshes, the filling of lowlands and reclamation [51]. With the recognition that natural wetlands are important wildlife and biodiversity resources, such measures have largely ceased in many countries [20]. Therefore, more recently, the second method (preventing variations in water levels) has been preferred, for example, with the creation of ditches or the installation of pumps that maintain the water level constant on land periodically subjected to flooding. This also allows us to safeguard or even increase fauna, especially aquatic fauna that compete with or prey on mosquito larvae [51].

The major problems in this kind of approach (prevention) are encountered when land is periodically submerged due to human activity that benefits from it. A typical example is a rice field cultivated under alternating submergence conditions. In this case, the reasons for those who have to manage the problems arising from mosquito annoyance often clash with the legitimate interests of farmers, and a solution that satisfies both parties cannot be found.

Another fundamental approach to eliminate floodwater mosquitoes is larvicidal control, especially where standing water cannot be reduced or eliminated. Since the trigger for the hatching of eggs and the consequent presence of larvae at breeding sites is an increase in the water level, by monitoring the latter, it is possible to predict larval infestation slightly earlier. If the rise in water levels can be predicted and is uniform throughout the territory, larvicidal intervention can be easily programmed. In contrast, if this phenomenon is unpredictable and/or spreads across the territory, larvicidal treatment will be difficult to successfully implement. Aerial intervention, usually conducted by helicopters and drones, is often the best solution for reaching all infested surfaces within the short time available before larval pupation, but it requires adequate permits, funding and acceptance by the population [52]. Aerial interventions often require refinements from the ground. In recent decades, biorational pesticides, such as *Bacillus thuringiensis* serovar *israelensis*, have replaced synthetic larvicides almost everywhere [53]. In some cases, granular formulations of these products can be applied to the ground just before flooding, allowing more efficient action of the active ingredient on newly hatched larvae [40].

A common approach is adulticidal control. Unfortunately, this is often the only method used by urban communities to oppose the periodic invasions of adult mosquitoes in residential areas. This method exhibits large and important gaps. First, it acts directly on the life stage that is already creating the problem rather than preventing it. Moreover, there are no adulticidal products that are completely safe and produce a low environmental impact. Moreover, adulticide use can cause resistance in mosquitoes, decreasing their effectiveness in the event of an epidemic [54]. Appropriate thresholds can be defined as indications for adulticide treatments to avoid unnecessary treatments.

The last possible and least effective approach is personal protection: mosquito screens, repellents and other devices can help avoid bites in particular situations but cannot guarantee total protection.

## Conclusions

The data presented in this study could allow the identification of areas at high risk, providing the possibility to optimise and reinforce entomological surveillance. The detailed characterisation of the distribution of the two mosquito species in the surveyed area could be utilised for risk assessment of diseases potentially transmitted by these two mosquito species. These data can be useful for evaluating the appropriate control interventions in the case of an outbreak of a disease spread by one of these mosquito species or for limiting their nuisance.

## Supplementary Information

Additional file 1

## Data Availability

No datasets were generated or analysed during the current study.

## Abbreviations

AUC: Area under the curve
LAUs: Local administrative units
NUT: Nomenclature of territorial units
RMSE: Root mean square error
RVF: Rift Valley fever
SDM: Species distribution modelling
THAV: Tahyna virus
WNV: West Nile virus
