# Supplementary material for: Distribution and abundance of Aedes caspius (Pallas, 1771) and Aedes vexans (Meigen, 1830) in the Po Plain (northern Italy)
Source: Parasit Vectors. 2024 Nov 5;17:452. doi: 10.1186/s13071-024-06527-8 (PMC11539340; doi:10.1186/s13071-024-06527-8)

SUPPLEMENTAL MATERIAL

Table S1. List of screened covariates (Cov.) with reference to those selected (Se.) for implementing the models.

**Variable Source**	(Reference)	Time	Cov	Se
Middle Infra-red (MIR)	https://www.palebludata.com/	2001–2016	14	5
Day-time land surface t (DLST)			14	4
Night-time land surface t (NLST)			14	4
Enhanced vegetation index (EVI)			14	3
Norm. dif. veg. index (NDVI)			14	2
Monthly and total precipitation	https://www.worldclim.org/	1970–2000	13	3
Monthly t (min, max and mean)			36	-
Bioclimatic variables			19	4
Altitude and slope (Globe DEM)	https://www.ngdc.noaa.gov/mgg/topo/globe.html	2011	2	2
Corine land cover	https://land.copernicus.eu/	2018	3	3
Tree cover density		2018	1	1
Water bodies < 1 km2	EU-Hydro, Openstreetmap.org	2018	1	1
Soil suborders (USDA)	ISRIC 1 km^2grid www. isric. org	2018	1	1
Drainage basins	ING www. pcn. minam biente. it/ mattm	2010		
			148	34

Table S2 Descriptive statistics of the geostatistical analysis data

	Aedes caspius (224)		*Aedes vexans* (205)	
	Mean	log10	Mean	log10
Mean (SD)	58.647 (195.4)	0.887 (0.988)	17.16 (85.32)	0.137 (0.928)
Range			004-885.7 (885.7)	4.35 2.95
Min–max (range)	0.04–2142 (2142)	-1.398–3,33 (4.35)	0.04–885.7 (885.7)	-1.398–2.95 (4,35)
Skewness	7.73 (0.2)	-0.2 (0.2)	8.93 (0.2)	0.39 (0.2)
Kurtosis	69.95 (0.32)	-0.602 (0.32)	84.13 (0.34)	0.191 (0.34)

Table S2. Reference to numeric values of the ordinary kriging interpolations and Maxent models of the two mosquitoes to the municipalities in the surveyed area. Quartile classifications are also reported.

Munic code	Comune	log_Ae. caspius	quartile class	log_Ae. vexans	quartile class	prob_Ae. caspius	quartile class	prob_Ae. vexans	quartile class
1065	Castagnole Piemonte	-0.126	low	-0.057	medium high	0.139	medium low	0.355	medium low
1090	Collegno	0.028	medium low	0.542	high	0.072	low	0.298	medium low
1025	Bibiana	0.000	low	0.000	medium high	0.094	medium low	0.097	low
1046	Cafasse	-0.076	low	0.360	high	0.019	low	0.076	low
1047	Caluso	0.896	medium high	0.262	high	0.181	medium low	0.273	medium low
1049	Campiglione Fenile	0.000	low	0.000	medium high	0.166	medium low	0.223	low
1059	Carmagnola	0.259	medium low	-0.126	medium high	0.130	medium low	0.346	medium low

Munic code	Comune	log_Ae. caspius	quartile class	log_Ae. vexans	quartile class	prob_Ae. caspius	quartile class	prob_Ae. vexans	quartile class
1078	Chieri	0.934	medium high	0.193	high	0.124	medium low	0.313	medium low
1050	Candia Canavese	0.841	medium high	0.350	high	0.160	medium low	0.290	medium low
1051	Candiolo	-0.049	low	0.340	high	0.059	low	0.294	medium low
1048	Cambiano	0.705	medium high	0.287	high	0.091	medium low	0.342	medium low
1068	Castiglione Torinese	0.713	medium high	0.218	high	0.149	medium low	0.207	low
1015	Bairo	0.921	medium high	0.425	high	0.084	medium low	0.198	low
1016	Balangero	-0.058	low	0.525	high	0.010	low	0.057	low
1017	Baldissero Canavese	1.016	medium high	0.417	high	0.054	low	0.092	low
1069	Cavagnolo	1.388	medium high	-0.193	medium low	0.326	medium high	0.284	medium low
1094	Corio	0.060	medium low	0.529	high	0.002	low	0.012	low
1070	Cavour	-0.402	low	-0.145	medium high	0.087	medium low	0.243	low
1214	Rivalta di Torino	-0.340	low	0.393	high	0.075	low	0.244	low
1033	Bosconero	0.541	medium low	0.224	high	0.118	medium low	0.281	medium low
1034	Brandizzo	0.800	medium high	0.090	medium high	0.286	medium high	0.442	medium high
1071	Cercenasco	-0.257	low	-0.168	medium high	0.197	medium low	0.423	medium high
1035	Bricherasio	0.000	low	0.000	medium high	0.109	medium low	0.133	low
1001	Agliè	0.866	medium high	0.437	high	0.220	medium low	0.202	low
1002	Airasca	-0.354	low	0.051	medium high	0.272	medium high	0.412	medium low
1038	Bruino	-0.565	low	0.310	high	0.056	low	0.191	low
1058	Carignano	0.181	medium low	-0.026	medium high	0.164	medium low	0.492	medium high
1041	Buriasco	-0.444	low	-0.153	medium high	0.150	medium low	0.296	medium low
1039	Brusasco	1.441	medium high	-0.261	medium low	0.370	medium high	0.350	medium low
1083	Ciconio	0.825	medium high	0.432	high	0.155	medium low	0.295	medium low
1063	Caselle Torinese	0.304	medium low	0.338	high	0.087	medium low	0.318	medium low
1082	Chivasso	0.994	medium high	0.035	medium high	0.362	medium high	0.398	medium low
1008	Alpignano	-0.259	low	0.418	high	0.061	low	0.247	low
1009	Andezeno	1.062	medium high	0.059	medium high	0.210	medium low	0.257	low
1043	Busano	0.367	medium low	0.544	high	0.048	low	0.214	low
1064	Castagneto Po	1.081	medium high	-0.044	medium high	0.124	medium low	0.071	low
1066	Castellamonte	0.899	medium high	0.488	high	0.035	low	0.099	low
1021	Barbania	0.311	medium low	0.451	high	0.034	low	0.180	low
1023	Barone Canavese	0.835	medium high	0.334	high	0.156	medium low	0.221	low
1024	Beinasco	0.037	medium low	0.559	high	0.073	low	0.287	medium low
1028	Borgaro Torinese	0.375	medium low	0.435	high	0.109	medium low	0.387	medium low
1086	Cirié	0.087	medium low	0.319	high	0.020	low	0.206	low
1012	Arignano	1.097	medium high	-0.026	medium high	0.221	medium low	0.257	low

Munic code	Comune	log_Ae. caspius	quartile class	log_Ae. vexans	quartile class	prob_Ae. caspius	quartile class	prob_Ae. vexans	quartile class
1163	Moriondo Torinese	1.173	medium high	-0.162	medium high	0.213	medium low	0.191	low
1164	Nichelino	0.212	medium low	0.588	high	0.143	medium low	0.315	medium low
1142	Macello	-0.424	low	-0.201	medium low	0.099	medium low	0.296	medium low
1166	Nole	0.033	medium low	0.389	high	0.028	low	0.184	low
1183	Pecetto Torinese	0.795	medium high	0.473	high	0.065	low	0.123	low
1178	Pancalieri	-0.130	low	-0.246	medium low	0.151	medium low	0.475	medium high
1153	Mombello di Torino	1.121	medium high	-0.108	medium high	0.162	medium low	0.215	low
1097	Cumiana	-0.593	low	0.084	medium high	0.077	low	0.145	low
1191	Pinerolo	-0.523	low	-0.015	medium high	0.088	medium low	0.177	low
1187	Pertusio	0.384	medium low	0.541	high	0.040	low	0.095	low
1119	Grosso	0.044	medium low	0.500	high	0.011	low	0.121	low
1120	Grugliasco	0.051	medium low	0.569	high	0.060	low	0.264	low
1161	Montanaro	0.852	medium high	0.138	high	0.117	medium low	0.301	medium low
1162	Monteu da Po	1.338	medium high	-0.192	medium low	0.401	medium high	0.368	medium low
1098	Cuorgné	0.913	medium high	0.544	high	0.018	low	0.059	low
1189	Pianezza	-0.144	low	0.453	high	0.053	low	0.279	medium low
1123	Isolabella	0.711	medium high	-0.103	medium high	0.159	medium low	0.344	medium low
1099	Druento	-0.033	low	0.396	high	0.035	low	0.272	medium low
1195	Piscina	-0.522	low	-0.002	medium high	0.222	medium low	0.274	medium low
1156	Moncalieri	0.506	medium low	0.527	high	0.149	medium low	0.313	medium low
1159	Montalenghe	0.828	medium high	0.331	high	0.158	medium low	0.222	low
1101	Favria	0.404	medium low	0.543	high	0.087	medium low	0.281	medium low
1102	Feletto	0.571	medium high	0.250	high	0.117	medium low	0.260	low
1197	Poirino	0.649	medium high	-0.056	medium high	0.131	medium low	0.370	medium low
1146	Mathi	-0.019	low	0.463	high	0.015	low	0.114	low
1104	Fiano	-0.087	low	0.348	high	0.016	low	0.165	low
1127	La Loggia	0.378	medium low	0.360	high	0.165	medium low	0.493	medium high
1148	Mazzé	1.061	medium high	0.174	high	0.389	medium high	0.325	medium low
1109	Front	0.347	medium low	0.361	high	0.042	low	0.215	low
1129	Lauriano	1.270	medium high	-0.204	medium low	0.258	medium high	0.376	medium low
1168	None	-0.175	low	0.168	high	0.091	medium low	0.318	medium low
1110	Frossasco	-0.605	low	0.001	medium high	0.047	low	0.136	low
1111	Garzigliana	0.000	low	0.000	medium high	0.115	medium low	0.244	low
1130	Leinù	0.438	medium low	0.266	high	0.132	medium low	0.345	medium low
1112	Gassino Torinese	0.803	medium high	0.128	high	0.127	medium low	0.212	low

Munic code	Comune	log_Ae. caspius	quartile class	log_Ae. vexans	quartile class	prob_Ae. caspius	quartile class	prob_Ae. vexans	quartile class
1135	Lombardore	0.494	medium low	0.111	medium high	0.040	low	0.230	low
1106	Foglizzo	0.795	medium high	0.242	high	0.144	medium low	0.294	medium low
1193	Piobesi Torinese	0.030	medium low	0.106	medium high	0.047	low	0.331	medium low
1143	Maglione	1.428	medium high	-0.020	medium high	0.375	medium high	0.217	low
1170	Oglianico	0.526	medium low	0.473	high	0.066	low	0.268	low
1194	Piossasco	-0.499	low	0.232	high	0.053	low	0.142	low
1136	Lombriasco	-0.023	low	-0.206	medium low	0.189	medium low	0.513	medium high
1171	Orbassano	-0.159	low	0.434	high	0.068	low	0.278	medium low
1172	Orio Canavese	0.828	medium high	0.339	high	0.172	medium low	0.204	low
1173	Osasco	0.000	low	0.000	medium high	0.132	medium low	0.284	medium low
1203	Pralormo	0.466	medium low	-0.123	medium high	0.121	medium low	0.289	medium low
1141	Lusigliè	0.813	medium high	0.279	high	0.172	medium low	0.293	medium low
1174	Osasio	-0.026	low	-0.134	medium high	0.144	medium low	0.377	medium low
1176	Ozegna	0.849	medium high	0.430	high	0.104	medium low	0.255	low
1116	Givoletto	-0.286	low	0.426	high	0.029	low	0.050	low
2070	Lignana	1.933	high	-0.337	medium low	0.217	medium low	0.415	medium low
2131	San Germano Vercellese	1.790	high	-0.298	medium low	0.199	medium low	0.389	medium low
2082	Motta de' Conti	2.127	high	-0.274	medium low	0.454	medium high	0.459	medium high
2104	Prarolo	1.990	high	-0.323	medium low	0.245	medium low	0.442	medium high
3027	Briona	1.761	high	0.057	medium high	0.404	medium high	0.304	medium low
3030	Caltignaga	1.943	high	0.077	medium high	0.520	high	0.383	medium low
2047	Costanzana	2.029	high	-0.316	medium low	0.303	medium high	0.367	medium low
3012	Barengo	1.727	high	0.235	high	0.468	medium high	0.320	medium low
3016	Bellinzago Novarese	1.910	high	0.190	high	0.674	high	0.342	medium low
3018	Biandrate	1.807	high	-0.111	medium high	0.250	medium low	0.321	medium low
1219	Rivoli	-0.233	low	0.408	high	0.042	low	0.177	low
1220	Robassomero	0.027	medium low	0.324	high	0.043	low	0.256	low
2088	Olcenengo	1.832	high	-0.213	medium low	0.082	medium low	0.317	medium low
2158	Vercelli	1.922	high	-0.207	medium low	0.458	medium high	0.399	medium low
2142	Stroppiana	2.050	high	-0.313	medium low	0.229	medium low	0.377	medium low
1290	Vauda Canavese	0.323	medium low	0.362	high	0.024	low	0.161	low
2054	Desana	1.989	high	-0.328	medium low	0.293	medium high	0.413	medium low
2108	Quinto Vercellese	1.823	high	-0.151	medium high	0.297	medium high	0.392	medium low
1292	Venaria Reale	0.195	medium low	0.460	high	0.087	medium low	0.319	medium low
2006	Arborio	1.550	high	-0.132	medium high	0.354	medium high	0.315	medium low

Munic code	Comune	log_Ae. caspius	quartile class	log_Ae. vexans	quartile class	prob_Ae. caspius	quartile class	prob_Ae. vexans	quartile class
2163	Villarboit	1.685	high	-0.137	medium high	0.364	medium high	0.390	medium low
1215	Riva presso Chieri	0.994	medium high	-0.042	medium high	0.122	medium low	0.354	medium low
2133	Santhià	1.771	high	-0.253	medium low	0.501	medium high	0.420	medium high
2115	Rive	2.072	high	-0.329	medium low	0.241	medium low	0.358	medium low
3021	Bogogno	1.658	high	0.396	high	0.622	high	0.208	low
3023	Borgolavezzaro	2.066	high	-0.177	medium low	0.295	medium high	0.326	medium low
2122	Rovasenda	1.578	high	0.034	medium high	0.335	medium high	0.314	medium low
2116	Roasio	1.603	high	0.052	medium high	0.357	medium high	0.190	low
2147	Tricerro	1.998	high	-0.381	medium low	0.437	medium high	0.437	medium high
1301	Villanova Canavese	-0.032	low	0.324	high	0.028	low	0.216	low
2089	Oldenico	1.804	high	-0.149	medium high	0.468	medium high	0.436	medium high
2090	Palazzolo Vercellese	1.929	high	-0.439	medium low	0.512	high	0.466	medium high
1221	Rocca Canavese	0.084	medium low	0.529	high	0.011	low	0.052	low
1222	Roletto	0.000	low	0.000	medium high	0.017	low	0.076	low
2058	Fontanetto Po	1.811	high	-0.486	low	0.387	medium high	0.482	medium high
2071	Livorno Ferraris	1.590	high	-0.297	medium low	0.285	medium high	0.363	medium low
2091	Pertengo	2.022	high	-0.321	medium low	0.137	medium low	0.324	medium low
2164	Villata	1.829	high	-0.163	medium high	0.281	medium high	0.375	medium low
2065	Greggio	1.640	high	-0.152	medium high	0.599	high	0.407	medium low
3032	Cameri	2.022	high	0.212	high	0.676	high	0.383	medium low
2126	Salasco	1.840	high	-0.346	medium low	0.183	medium low	0.380	medium low
2127	Sali Vercellese	1.895	high	-0.325	medium low	0.162	medium low	0.368	medium low
2118	Ronsecco	1.896	high	-0.433	medium low	0.193	medium low	0.407	medium low
3024	Borgomanero	1.590	high	0.153	high	0.382	medium high	0.157	low
1304	Villareggia	1.203	medium high	0.103	medium high	0.382	medium high	0.287	medium low
3036	Carpignano Sesia	1.578	high	-0.019	medium high	0.575	high	0.246	low
3037	Casalbeltrame	1.835	high	-0.119	medium high	0.186	medium low	0.344	medium low
2059	Formigliana	1.722	high	-0.170	medium high	0.383	medium high	0.401	medium low
2062	Ghislarengo	1.537	high	-0.027	medium high	0.531	high	0.332	medium low
2072	Lozzolo	1.601	high	0.091	medium high	0.302	medium high	0.153	low
2049	Crescentino	1.607	high	-0.347	medium low	0.516	high	0.483	medium high
1225	Rondissone	1.141	medium high	0.064	medium high	0.581	high	0.376	medium low
2093	Pezzana	2.026	high	-0.387	medium low	0.201	medium low	0.385	medium low
2067	Lamporo	1.556	high	-0.302	medium low	0.222	medium low	0.345	medium low
3026	Briga Novarese	1.592	high	0.117	medium high	0.387	medium high	0.145	low

Munic code	Comune	log_Ae. caspius	quartile class	log_Ae. vexans	quartile class	prob_Ae. caspius	quartile class	prob_Ae. vexans	quartile class
2148	Trino	1.951	high	-0.426	medium low	0.484	medium high	0.473	medium high
2128	Saluggia	1.349	medium high	-0.106	medium high	0.435	medium high	0.408	medium low
2150	Tronzano Vercellese	1.763	high	-0.351	medium low	0.332	medium high	0.388	medium low
2045	Collobiano	1.778	high	-0.148	medium high	0.461	medium high	0.395	medium low
1251	San Ponso	0.496	medium low	0.546	high	0.051	low	0.193	low
2061	Gattinara	1.588	high	0.090	medium high	0.384	medium high	0.184	low
2079	Moncrivello	1.374	medium high	-0.042	medium high	0.426	medium high	0.326	medium low
2052	Crova	1.804	high	-0.387	medium low	0.236	medium low	0.382	medium low
1252	San Raffaele Cimena	0.841	medium high	0.070	medium high	0.178	medium low	0.208	low
2068	Lenta	1.565	high	0.054	medium high	0.497	medium high	0.263	low
2042	Cigliano	1.355	medium high	-0.044	medium high	0.276	medium high	0.293	medium low
1236	San Benigno Canavese	0.611	medium high	0.126	high	0.078	low	0.290	medium low
1244	San Giorgio Canavese	0.818	medium high	0.311	high	0.189	medium low	0.281	medium low
1246	San Giusto Canavese	0.806	medium high	0.284	high	0.166	medium low	0.266	low
1253	San Sebastiano da Po	1.148	medium high	-0.134	medium high	0.147	medium low	0.271	medium low
1254	San Secondo di Pinerolo	0.000	low	0.000	medium high	0.045	low	0.109	low
1310	Virle Piemonte	-0.122	low	-0.178	medium low	0.229	medium low	0.457	medium high
1287	Valperga	0.779	medium high	0.546	high	0.043	low	0.126	low
1237	San Carlo Canavese	0.147	medium low	0.354	high	0.010	low	0.141	low
2030	Caresana	2.076	high	-0.343	medium low	0.443	medium high	0.434	medium high
2031	Caresanablot	1.870	high	-0.151	medium high	0.311	medium high	0.330	medium low
2032	Carisio	1.767	high	-0.200	medium low	0.390	medium high	0.358	medium low
2003	Albano Vercellese	1.722	high	-0.158	medium high	0.500	medium high	0.415	medium low
2004	Alice Castello	1.736	high	-0.127	medium high	0.538	high	0.335	medium low
1217	Rivarolo Canavese	0.575	medium high	0.397	high	0.089	medium low	0.269	medium low
1248	San Maurizio Canavese	0.206	medium low	0.241	high	0.040	low	0.243	low
1300	Villafranca Piemonte	-0.307	low	-0.376	medium low	0.246	medium low	0.359	medium low
1272	Torino	0.397	medium low	0.648	high	0.118	medium low	0.251	low
2017	Borgo Vercelli	1.891	high	-0.174	medium low	0.382	medium high	0.412	medium low
1293	Verolengo	1.233	medium high	-0.131	medium high	0.657	high	0.529	medium high
2033	Casanova Elvo	1.764	high	-0.180	medium low	0.253	medium high	0.369	medium low
2011	Bianzè	1.686	high	-0.307	medium low	0.257	medium high	0.367	medium low
18180	Villanterio	1.942	high	-0.554	low	0.659	high	0.540	medium high
20042	Poggio Rusco	1.179	medium high	0.576	high	0.412	medium high	0.615	high

Munic code	Comune	log_Ae. caspius	quartile class	log_Ae. vexans	quartile class	prob_Ae. caspius	quartile class	prob_Ae. vexans	quartile class
18085	Magherno	1.959	high	-0.542	low	0.683	high	0.503	medium high
18086	Marcignago	2.069	high	-0.670	low	0.548	high	0.393	medium low
18087	Marzano	1.977	high	-0.692	low	0.519	high	0.425	medium high
17138	San Paolo	-0.163	low	0.069	medium high	0.075	low	0.614	high
34048	Zibello	0.115	medium low	1.442	high	0.062	low	0.724	high
35001	Albinea	0.196	medium low	0.389	high	0.136	medium low	0.195	low
20055	San Benedetto Po	1.220	medium high	1.048	high	0.529	high	0.710	high
18016	Borgo Priolo	2.000	high	-0.188	medium low	0.182	medium low	0.167	low
18075	Gravellona Lomellina	1.992	high	0.001	medium high	0.375	medium high	0.459	medium high
20010	Casalmoro	-0.355	low	0.190	high	0.123	medium low	0.627	high
20011	Casaloldo	-0.274	low	0.160	high	0.047	low	0.455	medium high
20012	Casalromano	0.059	medium low	0.696	high	0.007	low	0.388	medium low
17130	Paderno Franciacorta	-0.028	low	0.509	high	0.028	low	0.384	medium low
20013	Castelbelforte	1.560	high	-0.202	medium low	0.720	high	0.682	high
18003	Albonese	2.056	high	-0.224	medium low	0.475	medium high	0.288	medium low
18004	Albuzzano	2.071	high	-0.526	low	0.452	medium high	0.450	medium high
1240	San Francesco al Campo	0.259	medium low	0.216	high	0.015	low	0.214	low
2007	Asigliano Vercellese	1.983	high	-0.263	medium low	0.259	medium high	0.392	medium low
2035	San Giacomo Vercellese	1.593	high	-0.058	medium high	0.146	medium low	0.288	medium low
1249	San Mauro Torinese	0.666	medium high	0.325	high	0.135	medium low	0.211	low
1231	Salassa	0.872	medium high	0.553	high	0.078	low	0.239	low
1241	Sangano	-0.667	low	0.291	high	0.025	low	0.068	low
1218	Rivarossa	0.433	medium low	0.215	high	0.023	low	0.218	low
1280	Trofarello	0.647	medium high	0.352	high	0.066	low	0.307	medium low
1265	Settimo Torinese	0.630	medium high	0.264	high	0.231	medium low	0.429	medium high
1260	Scalenghe	-0.309	low	-0.066	medium high	0.170	medium low	0.331	medium low
1308	Villastellone	0.466	medium low	0.061	medium high	0.111	medium low	0.379	medium low
2021	Buronzo	1.661	high	-0.045	medium high	0.412	medium high	0.373	medium low
1314	Volpiano	0.634	medium high	0.136	high	0.178	medium low	0.388	medium low
1294	Verrua Savoia	1.585	high	-0.283	medium low	0.503	medium high	0.375	medium low
1243	San Gillio	-0.255	low	0.450	high	0.060	low	0.244	low
1273	Torrazza Piemonte	1.198	medium high	-0.052	medium high	0.556	high	0.420	medium high
1274	Torre Canavese	0.967	medium high	0.416	high	0.052	low	0.121	low
2009	Balocco	1.671	high	-0.131	medium high	0.619	high	0.422	medium high
1284	Val della Torre	-0.388	low	0.519	high	0.022	low	0.016	low

Munic code	Comune	log_Ae. caspius	quartile class	log_Ae. vexans	quartile class	prob_Ae. caspius	quartile class	prob_Ae. vexans	quartile class
1299	Vigone	-0.274	low	-0.271	medium low	0.147	medium low	0.401	medium low
1309	Vinovo	0.185	medium low	0.358	high	0.093	medium low	0.349	medium low
1315	Volvera	-0.322	low	0.226	high	0.064	low	0.253	low
2015	Borgo d'Ale	1.594	high	-0.117	medium high	0.329	medium high	0.242	low
4143	Moretta	-0.233	low	-0.322	medium low	0.067	low	0.302	medium low
4121	Martiniana Po	0.000	low	0.000	medium high	0.031	low	0.021	low
4114	Magliano Alpi	0.819	medium high	-0.191	medium low	0.005	low	0.106	low
4130	Mondovù	0.800	medium high	-0.229	medium low	0.007	low	0.107	low
4171	Polonghera	-0.162	low	-0.265	medium low	0.096	medium low	0.411	medium low
4181	Rifreddo	0.000	low	0.000	medium high	0.113	medium low	0.045	low
4179	Racconigi	-0.114	low	-0.210	medium low	0.151	medium low	0.337	medium low
4144	Morozzo	0.635	medium high	-0.285	medium low	0.031	low	0.138	low
4085	Envie	-0.377	low	-0.150	medium high	0.154	medium low	0.118	low
1216	Rivara	0.333	medium low	0.541	high	0.042	low	0.142	low
4116	Manta	-0.071	low	-0.427	medium low	0.208	medium low	0.151	low
1257	Santena	0.679	medium high	0.132	high	0.133	medium low	0.336	medium low
4094	Gambasca	0.000	low	0.000	medium high	0.053	low	0.032	low
4096	Genola	0.026	medium low	-0.300	medium low	0.065	low	0.184	low
4082	Dronero	0.221	medium low	-0.212	medium low	0.009	low	0.033	low
4180	Revello	-0.243	low	-0.394	medium low	0.221	medium low	0.202	low
4163	Peveragno	0.595	medium high	-0.338	medium low	0.002	low	0.029	low
4140	Monteu Roero	0.281	medium low	-0.121	medium high	0.054	low	0.239	low
4087	Faule	-0.189	low	-0.291	medium low	0.143	medium low	0.493	medium high
4118	Margarita	0.649	medium high	-0.314	medium low	0.028	low	0.120	low
4104	Lagnasco	-0.108	low	-0.411	medium low	0.199	medium low	0.230	low
4117	Marene	-0.253	low	-0.144	medium high	0.025	low	0.173	low
4078	Cuneo	0.440	medium low	-0.345	medium low	0.043	low	0.110	low
4128	Monasterolo di Savigliano	-0.205	low	-0.119	medium high	0.128	medium low	0.289	medium low
4146	Murello	-0.171	low	-0.221	medium low	0.063	low	0.271	medium low
4136	Montanera	0.554	medium high	-0.260	medium low	0.044	low	0.138	low
4165	Pianfei	0.706	medium high	-0.307	medium low	0.010	low	0.086	low
4147	Narzole	-0.114	low	-0.190	medium low	0.017	low	0.161	low
4189	Rocca de' Baldi	0.724	medium high	-0.276	medium low	0.026	low	0.161	low
4089	Fossano	0.216	medium low	-0.274	medium low	0.068	low	0.183	low

Munic code	Comune	log_Ae. caspius	quartile class	log_Ae. vexans	quartile class	prob_Ae. caspius	quartile class	prob_Ae. vexans	quartile class
4166	Piasco	0.020	low	-0.340	medium low	0.039	low	0.030	low
4138	Montemale di Cuneo	0.000	low	0.000	medium high	0.001	low	0.007	low
4028	Boves	0.523	medium low	-0.351	medium low	0.011	low	0.037	low
4058	Cavallerleone	-0.200	low	-0.145	medium high	0.054	low	0.282	medium low
4065	Cervere	-0.236	low	-0.276	medium low	0.064	low	0.206	low
4067	Cherasco	-0.321	low	-0.179	medium low	0.046	low	0.212	low
3097	Mezzomerico	1.735	high	0.439	high	0.603	high	0.221	low
4064	Cervasca	0.401	medium low	-0.369	medium low	0.019	low	0.069	low
3100	Momo	1.802	high	0.241	high	0.610	high	0.324	medium low
3073	Ghemme	1.598	high	0.153	high	0.323	medium high	0.177	low
3076	Gozzano	1.588	high	0.100	medium high	0.357	medium high	0.136	low
3158	Vespolate	2.092	high	-0.124	medium high	0.132	medium low	0.321	medium low
4042	Cardè	-0.305	low	-0.406	medium low	0.052	low	0.261	low
4043	Carrù	0.854	medium high	-0.178	medium low	0.015	low	0.142	low
3159	Vicolungo	1.720	high	-0.128	medium high	0.383	medium high	0.312	medium low
3164	Vinzaglio	1.978	high	-0.237	medium low	0.207	medium low	0.382	medium low
3055	Cressa	1.632	high	0.356	high	0.736	high	0.252	low
3058	Cureggio	1.582	high	0.225	high	0.456	medium high	0.181	low
4029	Bra	-0.238	low	-0.080	medium high	0.066	low	0.253	low
3141	Sozzago	2.015	high	0.171	high	0.337	medium high	0.426	medium high
3143	Suno	1.678	high	0.380	high	0.619	high	0.265	low
4075	Costigliole Saluzzo	0.040	medium low	-0.379	medium low	0.085	medium low	0.095	low
3104	Nibbiola	2.116	high	-0.051	medium high	0.084	medium low	0.330	medium low
3108	Oleggio	1.776	high	0.304	high	0.712	high	0.344	medium low
4025	Borgo San Dalmazzo	0.454	medium low	-0.358	medium low	0.011	low	0.055	low
3077	Granozzo con Monticello	2.086	high	-0.139	medium high	0.338	medium high	0.402	medium low
4009	Bagnolo Piemonte	0.000	low	0.000	medium high	0.090	medium low	0.075	low
3042	Castellazzo Novarese	1.760	high	-0.043	medium high	0.327	medium high	0.358	medium low
4040	Caraglio	0.301	medium low	-0.360	medium low	0.020	low	0.080	low
4068	Chiusa di Pesio	0.670	medium high	-0.330	medium low	0.002	low	0.049	low
3065	Fara Novarese	1.634	high	0.144	high	0.503	medium high	0.206	low
3044	Cavaglietto	1.668	high	0.328	high	0.462	medium high	0.250	low
3045	Cavaglio d'Agogna	1.634	high	0.326	high	0.164	medium low	0.169	low
3131	Romentino	1.934	high	0.297	high	0.694	high	0.481	medium high
4041	Caramagna Piemonte	-0.033	low	-0.183	medium low	0.102	medium low	0.319	medium low

Munic code	Comune	log_Ae. caspius	quartile class	log_Ae. vexans	quartile class	prob_Ae. caspius	quartile class	prob_Ae. vexans	quartile class
3066	Fontaneto d'Agogna	1.613	high	0.335	high	0.429	medium high	0.210	low
3144	Terdobbiate	2.080	high	0.052	medium high	0.150	medium low	0.375	medium low
4062	Ceresole Alba	0.182	medium low	-0.158	medium high	0.072	low	0.222	low
3134	San Nazzaro Sesia	1.747	high	-0.158	medium high	0.422	medium high	0.372	medium low
4059	Cavallermaggiore	-0.251	low	-0.075	medium high	0.099	medium low	0.292	medium low
3146	Tornaco	2.037	high	0.023	medium high	0.226	medium low	0.413	medium low
3149	Trecate	1.957	high	0.265	high	0.458	medium high	0.417	medium low
3082	Invorio	1.594	high	0.002	medium high	0.191	medium low	0.109	low
3083	Landiona	1.582	high	-0.136	medium high	0.692	high	0.302	medium low
4019	Bene Vagienna	0.374	medium low	-0.238	medium low	0.010	low	0.135	low
4049	Castelletto Stura	0.542	medium low	-0.297	medium low	0.021	low	0.111	low
3106	Novara	2.142	high	0.056	medium high	0.547	high	0.398	medium low
4061	Centallo	0.346	medium low	-0.296	medium low	0.025	low	0.127	low
3068	Galliate	2.009	high	0.276	high	0.622	high	0.355	medium low
3049	Cerano	1.816	high	0.227	high	0.401	medium high	0.420	medium low
3069	Garbagna Novarese	2.136	high	0.040	medium high	0.166	medium low	0.345	medium low
3153	Vaprio d'Agogna	1.724	high	0.390	high	0.698	high	0.268	low
3090	Mandello Vitta	1.686	high	-0.098	medium high	0.345	medium high	0.271	medium low
3091	Marano Ticino	1.680	high	0.391	high	0.787	high	0.251	low
3135	San Pietro Mosezzo	1.970	high	-0.085	medium high	0.285	medium high	0.351	medium low
3138	Sillavengo	1.606	high	-0.097	medium high	0.315	medium high	0.213	low
3071	Gattico	1.597	high	0.154	high	0.485	medium high	0.195	low
4034	Busca	0.190	medium low	-0.360	medium low	0.035	low	0.089	low
3139	Sizzano	1.612	high	0.202	high	0.330	medium high	0.170	low
4022	Bernezzo	0.359	medium low	-0.382	medium low	0.007	low	0.025	low
4048	Castellar	-0.135	low	-0.409	medium low	0.152	medium low	0.136	low
6181	Viguzzolo	2.208	high	-0.176	medium low	0.176	medium low	0.212	low
6183	Villalvernia	2.061	high	-0.229	medium low	0.149	medium low	0.187	low
4198	Ruffia	-0.222	low	-0.167	medium high	0.212	medium low	0.381	medium low
4012	Barge	-0.383	low	-0.216	medium low	0.110	medium low	0.135	low
4045	Casalgrasso	-0.104	low	-0.255	medium low	0.163	medium low	0.511	medium high
6186	Villaromagnano	2.072	high	-0.323	medium low	0.096	medium low	0.190	low
3039	Casaleggio Novara	1.823	high	-0.078	medium high	0.300	medium high	0.383	medium low
3040	Casalino	1.991	high	-0.192	medium low	0.245	medium low	0.393	medium low
3041	Casalvolone	1.846	high	-0.135	medium high	0.148	medium low	0.339	medium low

Munic code	Comune	log_Ae. caspius	quartile class	log_Ae. vexans	quartile class	prob_Ae. caspius	quartile class	prob_Ae. vexans	quartile class
3129	Recetto	1.680	high	-0.164	medium high	0.678	high	0.333	medium low
3130	Romagnano Sesia	1.604	high	0.125	high	0.265	medium high	0.139	low
4016	Beinette	0.632	medium high	-0.342	medium low	0.007	low	0.083	low
6142	Quattordio	1.748	high	-0.304	medium low	0.351	medium high	0.259	low
6089	Lu	2.174	high	0.114	medium high	0.419	medium high	0.215	low
6091	Masio	1.675	high	-0.237	medium low	0.428	medium high	0.303	medium low
6087	Isola Sant'Antonio	2.226	high	-0.309	medium low	0.809	high	0.549	medium high
6138	Pozzolo Formigaro	1.954	high	-0.234	medium low	0.118	medium low	0.150	low
6109	Morano sul Po	2.080	high	-0.338	medium low	0.572	high	0.510	medium high
6131	Pomaro Monferrato	2.176	high	0.087	medium high	0.796	high	0.547	medium high
6099	Moncestino	1.746	high	-0.349	medium low	0.812	high	0.325	medium low
6133	Pontestura	2.101	high	-0.305	medium low	0.890	high	0.434	medium high
6151	Sale	1.982	high	-0.310	medium low	0.492	medium high	0.312	medium low
6132	Pontecurone	2.357	high	-0.138	medium high	0.186	medium low	0.239	low
6122	Oviglio	1.699	high	-0.186	medium low	0.548	high	0.304	medium low
6114	Novi Ligure	1.960	high	-0.260	medium low	0.110	medium low	0.158	low
6094	Mirabello Monferrato	2.122	high	0.132	high	0.611	high	0.317	medium low
6123	Ozzano Monferrato	2.144	high	-0.147	medium high	0.573	high	0.293	medium low
12048	Cavaria con Premezzo	0.927	medium high	-0.004	medium high	0.594	high	0.221	low
12050	Cislago	0.529	medium low	-0.265	medium low	0.283	medium high	0.249	low
12040	Cassano Magnago	0.956	medium high	-0.034	medium high	0.528	high	0.247	low
12070	Gallarate	1.082	medium high	0.036	medium high	0.558	high	0.217	low
37012	Casalfiumanese	0.887	medium high	0.121	high	0.012	low	0.227	low
36036	San Cesario sul Panaro	0.090	medium low	-0.174	medium low	0.155	medium low	0.491	medium high
28013	Borgoricco	0.149	medium low	-0.258	medium low	0.619	high	0.680	high
28014	Bovolenta	0.820	medium high	-0.668	low	0.066	low	0.521	medium high
28015	Brugine	0.737	medium high	-0.725	low	0.243	medium low	0.630	high
36013	Fiorano Modenese	0.123	medium low	0.376	high	0.126	medium low	0.227	low
37009	Calderara di Reno	0.535	medium low	0.164	high	0.071	low	0.485	medium high
28061	Pernumia	0.622	medium high	-0.586	low	0.314	medium high	0.601	high
28062	Piacenza d'Adige	0.591	medium high	-0.560	low	0.671	high	0.772	high
37002	Argelato	1.024	medium high	0.214	high	0.124	medium low	0.592	high
36037	San Felice sul Panaro	1.740	high	0.792	high	0.294	medium high	0.611	high
36038	San Possidonio	1.977	high	1.082	high	0.418	medium high	0.604	high

Munic code	Comune	log_Ae. caspius	quartile class	log_Ae. vexans	quartile class	prob_Ae. caspius	quartile class	prob_Ae. vexans	quartile class
36006	Castelfranco Emilia	0.233	medium low	-0.315	medium low	0.137	medium low	0.458	medium high
36019	Maranello	0.128	medium low	0.233	high	0.113	medium low	0.180	low
36020	Marano sul Panaro	0.102	medium low	0.005	medium high	0.057	low	0.130	low
37053	San Giovanni in Persiceto	0.858	medium high	-0.307	medium low	0.167	medium low	0.569	medium high
37019	Castel Maggiore	0.778	medium high	0.388	high	0.081	medium low	0.579	high
37060	Zola Predosa	0.160	medium low	0.124	high	0.044	low	0.318	medium low
38003	Bondeno	1.354	medium high	0.668	high	0.391	medium high	0.701	high
37046	Ozzano dell'Emilia	1.106	medium high	-0.002	medium high	0.033	low	0.334	medium low
37030	Granarolo dell'Emilia	1.022	medium high	0.500	high	0.093	medium low	0.517	medium high
37020	Castel San Pietro Terme	1.237	medium high	-0.044	medium high	0.032	low	0.286	medium low
37024	Crevalcore	1.371	medium high	-0.128	medium high	0.269	medium high	0.666	high
37050	Sala Bolognese	0.870	medium high	-0.029	medium high	0.138	medium low	0.612	high
37039	Molinella	1.561	high	0.835	high	0.285	medium high	0.691	high
37025	Dozza	1.228	medium high	-0.171	medium high	0.042	low	0.326	medium low
37037	Medicina	1.481	high	0.393	high	0.145	medium low	0.592	high
40019	Meldola	-0.272	low	-0.194	medium low	0.012	low	0.191	low
6168	Strevi	1.453	medium high	-0.650	low	0.297	medium high	0.169	low
6187	Visone	1.313	medium high	-0.318	medium low	0.091	medium low	0.134	low
6188	Volpedo	2.175	high	-0.136	medium high	0.181	medium low	0.156	low
6189	Volpeglino	2.206	high	-0.125	medium high	0.242	medium low	0.182	low
6177	Valenza	1.976	high	0.075	medium high	0.694	high	0.404	medium low
6171	Terruggia	2.250	high	0.010	medium high	0.495	medium high	0.261	low
6173	Ticineto	2.257	high	0.075	medium high	0.802	high	0.559	medium high
6178	Valmacca	2.202	high	0.003	medium high	0.895	high	0.654	high
4192	Roccavione	0.436	medium low	-0.098	medium high	0.004	low	0.041	low
6174	Tortona	2.037	high	-0.285	medium low	0.253	medium high	0.235	low
6167	Stazzano	0.000	low	0.000	medium high	0.042	low	0.145	low
6185	Villanova Monferrato	2.154	high	-0.246	medium low	0.525	high	0.496	medium high
13064	Cermenate	0.014	low	-0.404	medium low	0.654	high	0.214	low
13159	Mozzate	0.471	medium low	-0.269	medium low	0.343	medium high	0.230	low
13201	Rovellasca	0.284	medium low	-0.348	medium low	0.589	high	0.254	low
13012	Arosio	-0.150	low	-1.004	low	0.361	medium high	0.216	low
13128	Limido Comasco	0.277	medium low	-0.338	medium low	0.469	medium high	0.227	low
13131	Locate Varesino	0.468	medium low	-0.243	medium low	0.143	medium low	0.184	low

Munic code	Comune	log_Ae. caspius	quartile class	log_Ae. vexans	quartile class	prob_Ae. caspius	quartile class	prob_Ae. vexans	quartile class
13133	Lomazzo	0.149	medium low	-0.380	medium low	0.459	medium high	0.197	low
13163	Novedrate	0.116	medium low	-0.576	low	0.623	high	0.221	low
6086	Guazzora	2.219	high	-0.301	medium low	0.755	high	0.420	medium low
13045	Carbonate	0.466	medium low	-0.257	medium low	0.311	medium high	0.211	low
13046	Carimate	0.044	medium low	-0.524	low	0.666	high	0.225	low
13048	Carugo	-0.168	low	-1.002	low	0.308	medium high	0.167	low
13028	Bregnano	0.120	medium low	-0.389	medium low	0.497	medium high	0.215	low
13143	Mariano Comense	0.095	medium low	-1.010	low	0.379	medium high	0.197	low
6158	Sarezzano	2.137	high	-0.169	medium high	0.111	medium low	0.168	low
6096	Molino dei Torti	2.377	high	-0.264	medium low	0.756	high	0.387	medium low
13035	Cabiate	0.319	medium low	-0.885	low	0.466	medium high	0.203	low
6101	Monleale	2.155	high	-0.061	medium high	0.160	medium low	0.126	low
17133	Palazzolo sull'Oglio	-0.060	low	-0.051	medium high	0.058	low	0.410	medium low
17156	Provaglio d'Iseo	0.095	medium low	0.770	high	0.035	low	0.200	low
18019	Bornasco	2.013	high	-0.706	low	0.718	high	0.468	medium high
18020	Bosnasco	1.417	medium high	-0.167	medium high	0.530	high	0.391	medium low
17201	Villanuova sul Clisi	-0.716	low	-0.092	medium high	0.013	low	0.049	low
34036	Soragna	0.366	medium low	1.080	high	0.018	low	0.445	medium high
18008	Barbianello	1.825	high	-0.684	low	0.460	medium high	0.519	medium high
18009	Bascapè	1.772	high	-0.889	low	0.720	high	0.512	medium high
34037	Sorbolo	0.381	medium low	1.265	high	0.117	medium low	0.586	high
18011	Bastida Pancarana	2.357	high	-0.417	medium low	0.631	high	0.607	high
30114	Talmassons	0.319	medium low	0.131	high	0.230	medium low	0.530	medium high
17177	Seniga	-0.015	low	0.681	high	0.011	low	0.531	medium high
35014	Castellarano	0.081	medium low	0.504	high	0.106	medium low	0.194	low
35026	Luzzara	1.139	medium high	1.473	high	0.502	medium high	0.678	high
35027	Montecchio Emilia	-0.019	low	-0.027	medium high	0.063	low	0.355	medium low
17190	Trenzano	-0.123	low	0.053	medium high	0.064	low	0.545	medium high
18012	Battuda	1.966	high	-0.767	low	0.484	medium high	0.386	medium low
18037	Casteggio	2.059	high	-0.367	medium low	0.256	medium high	0.244	low
18038	Castelletto di Branduzzo	2.284	high	-0.402	medium low	0.544	high	0.473	medium high
18039	Castello d'Agogna	2.052	high	-0.468	low	0.502	medium high	0.415	medium low
18022	Breme	2.184	high	-0.161	medium high	0.743	high	0.598	high
20052	Roncoferraro	1.484	high	0.488	high	0.664	high	0.711	high

Munic code	Comune	log_Ae. caspius	quartile class	log_Ae. vexans	quartile class	prob_Ae. caspius	quartile class	prob_Ae. vexans	quartile class
17125	Orzinuovi	-0.053	low	0.163	high	0.118	medium low	0.635	high
18013	Belgioioso	1.874	high	-0.479	low	0.706	high	0.617	high
20053	Roverbella	0.970	medium high	-0.469	low	0.679	high	0.677	high
18191	Cornale e Bastida	2.435	high	-0.253	medium low	0.799	high	0.424	medium high
12075	Gerenzano	0.591	medium high	-0.263	medium low	0.331	medium high	0.271	medium low
19003	Annicco	0.353	medium low	0.379	high	0.127	medium low	0.618	high
19004	Azzanello	0.228	medium low	0.212	high	0.126	medium low	0.611	high
19005	Bagnolo Cremasco	0.670	medium high	-0.416	medium low	0.358	medium high	0.569	medium high
18115	Pinarolo Po	2.067	high	-0.595	low	0.635	high	0.498	medium high
18116	Pizzale	2.330	high	-0.322	medium low	0.493	medium high	0.429	medium high
18162	Travacù Siccomario	2.516	high	-0.490	low	0.825	high	0.670	high
18163	Trivolzio	2.028	high	-0.714	low	0.766	high	0.406	medium low
19107	Torre de' Picenardi	0.259	medium low	1.215	high	0.067	low	0.633	high
19108	Torricella del Pizzo	0.277	medium low	1.490	high	0.082	medium low	0.769	high
18129	Roncaro	2.069	high	-0.660	low	0.576	high	0.439	medium high
18110	Pavia	2.308	high	-0.554	low	0.742	high	0.501	medium high
18164	Tromello	2.637	high	-0.230	medium low	0.654	high	0.401	medium low
19040	Derovere	0.235	medium low	1.501	high	0.010	low	0.511	medium high
19041	Dovera	0.825	medium high	-0.581	low	0.333	medium high	0.573	medium high
18130	Rosasco	2.041	high	-0.511	low	0.346	medium high	0.481	medium high
18131	Rovescala	0.000	low	0.000	medium high	0.261	medium high	0.221	low
18173	Vellezzo Bellini	1.956	high	-0.747	low	0.797	high	0.473	medium high
18174	Verretto	2.157	high	-0.388	medium low	0.432	medium high	0.479	medium high
18153	Stradella	1.459	medium high	-0.541	low	0.455	medium high	0.355	medium low
18154	Suardi	1.952	high	-0.167	medium high	0.771	high	0.637	high
16198	Seriate	-0.494	low	-0.752	low	0.035	low	0.229	low
19042	Drizzona	0.181	medium low	0.862	high	0.025	low	0.495	medium high
19043	Fiesco	0.458	medium low	-0.056	medium high	0.185	medium low	0.617	high
20031	Marcaria	0.418	medium low	0.381	high	0.402	medium high	0.701	high
18175	Verrua Po	2.294	high	-0.549	low	0.748	high	0.514	medium high
18176	Vidigulfo	1.914	high	-0.777	low	0.763	high	0.498	medium high
19058	Monte Cremasco	0.736	medium high	-0.543	low	0.198	medium low	0.474	medium high
19059	Montodine	0.884	medium high	-0.022	medium high	0.384	medium high	0.603	high
19027	Castelvisconti	0.143	medium low	0.273	high	0.070	low	0.628	high
19028	Cella Dati	0.162	medium low	1.560	high	0.014	low	0.559	medium high

Munic code	Comune	log_Ae. caspius	quartile class	log_Ae. vexans	quartile class	prob_Ae. caspius	quartile class	prob_Ae. vexans	quartile class
19029	Chieve	0.735	medium high	-0.321	medium low	0.386	medium high	0.622	high
19030	Cicognolo	0.237	medium low	1.438	high	0.003	low	0.449	medium high
19031	Cingia de' Botti	0.236	medium low	1.452	high	0.038	low	0.620	high
16152	Osio Sopra	-0.259	low	-0.689	low	0.121	medium low	0.286	medium low
96015	Castelletto Cervo	1.651	high	0.007	medium high	0.584	high	0.367	medium low
96016	Cavagliù	1.818	high	-0.108	medium high	0.640	high	0.356	medium low
96017	Cerreto Castello	1.693	high	0.070	medium high	0.632	high	0.264	low
4250	Vottignasco	0.100	medium low	-0.327	medium low	0.069	low	0.191	low
93020	Fanna	0.000	low	0.000	medium high	0.001	low	0.084	low
5052	Dusino San Michele	0.898	medium high	-0.193	medium low	0.138	medium low	0.272	medium low
5012	Buttigliera d'Asti	1.174	medium high	-0.193	medium low	0.154	medium low	0.266	low
93018	Cordovado	0.628	medium high	0.183	high	0.284	medium high	0.424	medium high
4209	Sanfront	0.000	low	0.000	medium high	0.043	low	0.014	low
4243	Vignolo	0.427	medium low	-0.384	medium low	0.010	low	0.055	low
93004	Aviano	-0.888	low	-0.698	low	0.013	low	0.107	low
5036	Cerro Tanaro	1.618	high	-0.260	medium low	0.547	high	0.342	medium low
4244	Villafalletto	0.096	medium low	-0.348	medium low	0.098	medium low	0.183	low
4245	Villanova Mondovù	0.738	medium high	0.000	medium high	0.006	low	0.064	low
93021	Fiume Veneto	-0.085	low	-0.269	medium low	0.123	medium low	0.429	medium high
5028	Castello di Annone	1.636	high	-0.288	medium low	0.458	medium high	0.297	medium low
4232	Trinitù	0.525	medium low	-0.263	medium low	0.025	low	0.132	low
4246	Villanova Solaro	-0.192	low	-0.233	medium low	0.154	medium low	0.402	medium low
4247	Villar San Costanzo	0.187	medium low	-0.170	medium high	0.032	low	0.041	low
4234	Valgrana	0.000	low	0.000	medium high	0.001	low	0.011	low
4240	Verzuolo	-0.027	low	-0.410	medium low	0.138	medium low	0.110	low
4208	Sanfrè	-0.126	low	-0.124	medium high	0.056	low	0.256	low
5033	Cellarengo	0.642	medium high	-0.089	medium high	0.133	medium low	0.285	medium low
93002	Arba	-1.222	low	-0.262	medium low	0.002	low	0.149	low
93005	Azzano Decimo	0.251	medium low	-0.266	medium low	0.167	medium low	0.502	medium high
6075	Frugarolo	1.774	high	-0.150	medium high	0.225	medium low	0.239	low
93008	Budoia	-0.400	low	-0.644	low	0.015	low	0.093	low
93009	Caneva	0.173	medium low	-0.392	medium low	0.014	low	0.121	low
4215	Savigliano	-0.076	low	-0.282	medium low	0.075	low	0.220	low
4217	Scarnafigi	-0.198	low	-0.219	medium low	0.126	medium low	0.289	medium low
17060	Comezzano-Cizzago	-0.085	low	0.016	medium high	0.045	low	0.482	medium high

Munic code	Comune	log_Ae. caspius	quartile class	log_Ae. vexans	quartile class	prob_Ae. caspius	quartile class	prob_Ae. vexans	quartile class
17061	Concesio	-0.312	low	0.166	high	0.016	low	0.087	low
4202	Salmour	-0.009	low	-0.289	medium low	0.031	low	0.140	low
4203	Saluzzo	-0.193	low	-0.401	medium low	0.180	medium low	0.229	low
6076	Fubine	1.949	high	-0.322	medium low	0.419	medium high	0.299	medium low
93017	Cordenons	-0.804	low	-0.408	medium low	0.065	low	0.313	medium low
4211	Sant'Albano Stura	0.544	medium low	-0.238	medium low	0.061	low	0.167	low
93010	Casarsa della Delizia	-0.326	low	-0.010	medium high	0.211	medium low	0.434	medium high
93011	Castelnovo del Friuli	0.000	low	0.000	medium high	0.015	low	0.106	low
93012	Cavasso Nuovo	0.000	low	0.000	medium high	0.001	low	0.090	low
4228	Torre San Giorgio	-0.221	low	-0.297	medium low	0.137	medium low	0.287	medium low
4225	Tarantasca	0.247	medium low	-0.332	medium low	0.031	low	0.122	low
17045	Castrezzato	-0.051	low	0.011	medium high	0.059	low	0.496	medium high
17046	Cazzago San Martino	0.008	low	0.345	high	0.047	low	0.381	medium low
17059	Cologne	0.012	low	0.130	high	0.044	low	0.327	medium low
5096	Rocchetta Tanaro	1.539	high	-0.225	medium low	0.299	medium high	0.272	medium low
93013	Chions	0.357	medium low	-0.081	medium high	0.261	medium high	0.573	medium high
4214	Santo Stefano Roero	0.312	medium low	-0.164	medium high	0.033	low	0.187	low
6074	Fresonara	1.741	high	-0.180	medium low	0.220	medium low	0.248	low
4223	Sommariva Perno	0.029	medium low	-0.143	medium high	0.031	low	0.190	low
93007	Brugnera	0.205	medium low	-0.364	medium low	0.069	low	0.417	medium low
17048	Cellatica	-0.252	low	0.205	high	0.015	low	0.206	low
4222	Sommariva del Bosco	-0.058	low	-0.154	medium high	0.077	low	0.255	low
5101	San Paolo Solbrito	1.042	medium high	-0.278	medium low	0.198	medium low	0.341	medium low
17080	Gottolengo	-0.354	low	0.137	high	0.025	low	0.535	medium high
17039	Carpenedolo	-0.750	low	-0.142	medium high	0.098	medium low	0.572	medium high
6029	Capriata d'Orba	1.691	high	-0.263	medium low	0.197	medium low	0.195	low
17040	Castegnato	-0.150	low	0.315	high	0.037	low	0.423	medium high
17041	Castelcovati	-0.034	low	-0.063	medium high	0.057	low	0.442	medium high
17056	Coccaglio	0.020	low	0.169	high	0.046	low	0.395	medium low
17033	Calvagese della Riviera	-0.867	low	-0.122	medium high	0.045	low	0.233	low
17042	Castel Mella	-0.353	low	-0.014	medium high	0.043	low	0.480	medium high
6047	Castellazzo Bormida	1.673	high	-0.159	medium high	0.506	high	0.296	medium low
17091	Lograto	-0.219	low	0.070	medium high	0.054	low	0.494	medium high
17093	Longhena	-0.276	low	-0.100	medium high	0.048	low	0.521	medium high
17081	Gussago	-0.198	low	0.288	high	0.017	low	0.221	low

Munic code	Comune	log_Ae. caspius	quartile class	log_Ae. vexans	quartile class	prob_Ae. caspius	quartile class	prob_Ae. vexans	quartile class
6073	Frassineto Po	2.214	high	-0.045	medium high	0.819	high	0.600	high
17043	Castenedolo	-0.728	low	-0.284	medium low	0.037	low	0.435	medium high
6068	Felizzano	1.810	high	-0.327	medium low	0.441	medium high	0.290	medium low
17103	Manerbio	-0.362	low	-0.091	medium high	0.031	low	0.581	high
17066	Dello	-0.368	low	-0.232	medium low	0.055	low	0.583	high
17086	Isorella	-0.429	low	0.068	medium high	0.042	low	0.618	high
17069	Erbusco	0.043	medium low	0.287	high	0.042	low	0.323	medium low
17092	Lonato del Garda	-0.981	low	-0.162	medium high	0.134	medium low	0.419	medium low
6077	Gabiano	1.865	high	-0.336	medium low	0.813	high	0.320	medium low
5089	Refrancore	1.862	high	-0.278	medium low	0.356	medium high	0.345	medium low
17078	Ghedi	-0.661	low	-0.404	medium low	0.047	low	0.542	medium high
17057	Collebeato	-0.337	low	0.119	medium high	0.014	low	0.155	low
6011	Balzola	2.094	high	-0.336	medium low	0.389	medium high	0.508	medium high
17034	Calvisano	-0.649	low	-0.198	medium low	0.054	low	0.588	high
17064	Corzano	-0.193	low	-0.081	medium high	0.072	low	0.606	high
6082	Giarole	2.141	high	0.157	high	0.806	high	0.517	medium high
17077	Gavardo	-0.760	low	-0.099	medium high	0.027	low	0.116	low
5112	Valfenera	0.756	medium high	-0.122	medium high	0.148	medium low	0.278	medium low
6012	Basaluzzo	1.764	high	-0.177	medium low	0.127	medium low	0.160	low
6071	Frascaro	1.612	high	-0.192	medium low	0.348	medium high	0.219	low
6078	Gamalero	1.592	high	-0.241	medium low	0.364	medium high	0.246	low
17088	Leno	-0.508	low	-0.281	medium low	0.034	low	0.585	high
17037	Capriano del Colle	-0.410	low	-0.298	medium low	0.037	low	0.512	medium high
17038	Capriolo	-0.051	low	0.167	high	0.039	low	0.219	low
17097	Maclodio	-0.171	low	0.025	medium high	0.066	low	0.490	medium high
17052	Chiari	0.008	low	-0.016	medium high	0.076	low	0.511	medium high
17053	Cigole	-0.302	low	0.083	medium high	0.031	low	0.594	high
17071	Fiesse	-0.061	low	0.583	high	0.014	low	0.467	medium high
17072	Flero	-0.431	low	-0.263	medium low	0.038	low	0.534	medium high
17073	Gambara	-0.178	low	0.452	high	0.023	low	0.519	medium high
17099	Mairano	-0.299	low	-0.100	medium high	0.045	low	0.475	medium high
6147	Rocca Grimalda	1.656	high	-0.347	medium low	0.207	medium low	0.175	low
6127	Pasturana	1.784	high	-0.209	medium low	0.190	medium low	0.189	low
6162	Silvano d'Orba	1.665	high	-0.184	medium low	0.189	medium low	0.290	medium low

Munic code	Comune	log_Ae. caspius	quartile class	log_Ae. vexans	quartile class	prob_Ae. caspius	quartile class	prob_Ae. vexans	quartile class
6105	Montecastello	1.841	high	-0.163	medium high	0.359	medium high	0.275	medium low
6161	Sezzadio	1.592	high	-0.300	medium low	0.364	medium high	0.215	low
6129	Pietra Marazzi	1.823	high	-0.138	medium high	0.387	medium high	0.340	medium low
6130	Piovera	1.865	high	-0.235	medium low	0.613	high	0.364	medium low
6030	Carbonara Scrivia	2.040	high	-0.252	medium low	0.214	medium low	0.198	low
6112	Morsasco	1.358	medium high	-0.595	low	0.108	medium low	0.106	low
6031	Carentino	1.620	high	-0.115	medium high	0.486	medium high	0.256	low
6163	Solero	1.828	high	-0.174	medium low	0.267	medium high	0.259	low
5115	Viarigi	1.880	high	-0.377	medium low	0.171	medium low	0.297	medium low
6015	Bergamasco	1.616	high	-0.204	medium low	0.443	medium high	0.260	low
6046	Castellar Guidobono	2.216	high	-0.131	medium high	0.233	medium low	0.214	low
6119	Orsara Bormida	1.523	high	-0.629	low	0.240	medium low	0.147	low
6144	Rivalta Bormida	1.527	high	-0.548	low	0.345	medium high	0.200	low
6145	Rivarone	1.849	high	-0.175	medium low	0.514	high	0.391	medium low
6160	Serravalle Scrivia	2.105	high	-0.252	medium low	0.094	medium low	0.166	low
12109	Origgio	0.822	medium high	-0.416	medium low	0.199	medium low	0.216	low
6140	Predosa	1.672	high	-0.240	medium low	0.235	medium low	0.195	low
6115	Occimiano	2.204	high	0.119	medium high	0.429	medium high	0.366	medium low
6141	Quargnento	1.937	high	-0.145	medium high	0.449	medium high	0.235	low
12042	Castellanza	0.962	medium high	-0.072	medium high	0.455	medium high	0.266	low
12044	Castelseprio	0.550	medium low	-0.177	medium low	0.329	medium high	0.191	low
12026	Busto Arsizio	1.200	medium high	0.043	medium high	0.537	high	0.261	low
12029	Cairate	0.662	medium high	-0.143	medium high	0.432	medium high	0.253	low
12032	Cardano al Campo	1.316	medium high	0.155	high	0.489	medium high	0.225	low
12098	Marnate	0.818	medium high	-0.142	medium high	0.489	medium high	0.233	low
12033	Carnago	0.629	medium high	-0.147	medium high	0.421	medium high	0.198	low
12034	Caronno Pertusella	0.844	medium high	-0.492	low	0.273	medium high	0.218	low
12078	Gorla Maggiore	0.642	medium high	-0.186	medium low	0.209	medium low	0.209	low
12079	Gorla Minore	0.717	medium high	-0.175	medium low	0.343	medium high	0.229	low
12080	Gornate Olona	0.436	medium low	-0.215	medium low	0.410	medium high	0.201	low
12039	Casorate Sempione	1.273	medium high	0.149	high	0.212	medium low	0.176	low
12067	Fagnano Olona	0.780	medium high	-0.112	medium high	0.447	medium high	0.240	low
12068	Ferno	1.464	medium high	0.201	high	0.495	medium high	0.292	medium low
12108	Olgiate Olona	0.932	medium high	-0.072	medium high	0.528	high	0.261	low

Munic code	Comune	log_Ae. caspius	quartile class	log_Ae. vexans	quartile class	prob_Ae. caspius	quartile class	prob_Ae. vexans	quartile class
12089	Lonate Ceppino	0.482	medium low	-0.208	medium low	0.263	medium high	0.214	low
12090	Lonate Pozzolo	1.636	high	0.208	high	0.430	medium high	0.301	medium low
13202	Rovello Porro	0.372	medium low	-0.322	medium low	0.551	high	0.263	low
18145	San Zenone al Po	1.641	high	-0.289	medium low	0.626	high	0.602	high
19016	Casalbuttano ed Uniti	0.133	medium low	0.403	high	0.024	low	0.492	medium high
19017	Casale Cremasco-Vidolasco	0.196	medium low	-0.337	medium low	0.127	medium low	0.432	medium high
19018	Casaletto Ceredano	0.889	medium high	-0.231	medium low	0.469	medium high	0.604	high
19019	Casaletto di Sopra	0.198	medium low	-0.144	medium high	0.137	medium low	0.530	medium high
19020	Casaletto Vaprio	0.411	medium low	-0.371	medium low	0.168	medium low	0.507	medium high
19086	Romanengo	0.258	medium low	-0.093	medium high	0.100	medium low	0.467	medium high
19087	Salvirola	0.283	medium low	0.037	medium high	0.198	medium low	0.602	high
19088	San Bassano	0.607	medium high	0.296	high	0.201	medium low	0.616	high
13227	Turate	0.370	medium low	-0.320	medium low	0.325	medium high	0.210	low
20049	Revere	1.026	medium high	0.265	high	0.818	high	0.811	high
19089	San Daniele Po	0.069	medium low	1.592	high	0.049	low	0.722	high
18090	Mezzana Bigli	2.399	high	-0.269	medium low	0.815	high	0.605	high
15002	Abbiategrasso	1.669	high	-0.243	medium low	0.644	high	0.416	medium low
15005	Albairate	1.449	medium high	-0.419	medium low	0.733	high	0.449	medium high
18146	Sartirana Lomellina	2.153	high	-0.115	medium high	0.589	high	0.547	medium high
18147	Scaldasole	2.526	high	-0.252	medium low	0.341	medium high	0.399	medium low
18148	Semiana	2.218	high	-0.237	medium low	0.286	medium high	0.355	medium low
19021	Casalmaggiore	0.546	medium low	1.255	high	0.205	medium low	0.710	high
20007	Bozzolo	0.175	medium low	0.120	high	0.130	medium low	0.583	high
20008	Canneto sull'Oglio	0.097	medium low	0.560	high	0.013	low	0.426	medium high
20009	Carbonara di Po	1.086	medium high	0.152	high	0.793	high	0.781	high
20050	Rivarolo Mantovano	0.285	medium low	0.555	high	0.054	low	0.521	medium high
20051	Rodigo	0.454	medium low	0.210	high	0.458	medium high	0.636	high
15007	Arconate	1.453	medium high	0.068	medium high	0.495	medium high	0.378	medium low
15009	Arese	1.141	medium high	-0.568	low	0.662	high	0.372	medium low
15010	Arluno	1.243	medium high	-0.321	medium low	0.693	high	0.417	medium low
15011	Assago	1.537	high	-0.935	low	0.760	high	0.423	medium high
18070	Genzone	1.923	high	-0.439	medium low	0.542	high	0.548	medium high
12118	Samarate	1.307	medium high	0.120	high	0.528	high	0.260	low
12119	Saronno	0.585	medium high	-0.354	medium low	0.231	medium low	0.184	low

Munic code	Comune	log_Ae. caspius	quartile class	log_Ae. vexans	quartile class	prob_Ae. caspius	quartile class	prob_Ae. vexans	quartile class
18071	Gerenzago	1.948	high	-0.480	low	0.712	high	0.573	medium high
18072	Giussago	1.933	high	-0.739	low	0.712	high	0.452	medium high
19006	Bonemerse	-0.034	low	1.168	high	0.064	low	0.658	high
19007	Bordolano	0.059	medium low	0.367	high	0.121	medium low	0.685	high
13068	Cirimido	0.144	medium low	-0.380	medium low	0.633	high	0.225	low
19008	Ca' d'Andrea	0.257	medium low	1.323	high	0.026	low	0.592	high
19009	Calvatone	0.124	medium low	0.227	high	0.070	low	0.548	medium high
19010	Camisano	0.206	medium low	-0.273	medium low	0.087	medium low	0.447	medium high
19011	Campagnola Cremasca	0.364	medium low	-0.200	medium low	0.153	medium low	0.498	medium high
19077	Pozzaglio ed Uniti	-0.073	low	0.647	high	0.008	low	0.439	medium high
13100	Fenegrù	0.170	medium low	-0.370	medium low	0.440	medium high	0.200	low
19078	Quintano	0.378	medium low	-0.504	low	0.193	medium low	0.484	medium high
19079	Ricengo	0.285	medium low	-0.217	medium low	0.185	medium low	0.537	medium high
19080	Ripalta Arpina	0.761	medium high	-0.093	medium high	0.416	medium high	0.634	high
19081	Ripalta Cremasca	0.754	medium high	-0.144	medium high	0.372	medium high	0.570	medium high
12122	Solbiate Olona	0.844	medium high	-0.102	medium high	0.487	medium high	0.289	medium low
18068	Gambolù	2.327	high	-0.275	medium low	0.558	high	0.382	medium low
18141	Sant'Alessio con Vialone	2.130	high	-0.590	low	0.769	high	0.492	medium high
12123	Somma Lombardo	1.401	medium high	0.207	high	0.470	medium high	0.269	medium low
19012	Capergnanica	0.762	medium high	-0.225	medium low	0.266	medium high	0.526	medium high
19013	Cappella Cantone	0.491	medium low	0.361	high	0.204	medium low	0.643	high
19014	Cappella de' Picenardi	0.274	medium low	1.496	high	0.009	low	0.551	medium high
19015	Capralba	0.318	medium low	-0.435	medium low	0.251	medium high	0.537	medium high
19082	Ripalta Guerina	0.777	medium high	-0.073	medium high	0.470	medium high	0.634	high
19083	Rivarolo del Re ed Uniti	0.377	medium low	0.760	high	0.078	low	0.595	high
19084	Rivolta d'Adda	0.345	medium low	-1.037	low	0.529	high	0.559	medium high
19085	Robecco d'Oglio	-0.105	low	0.460	high	0.039	low	0.595	high
18069	Garlasco	2.489	high	-0.345	medium low	0.613	high	0.443	medium high
18144	Sant'Angelo Lomellina	1.994	high	-0.550	low	0.440	medium high	0.371	medium low
12127	Tradate	0.425	medium low	-0.242	medium low	0.235	medium low	0.178	low
12130	Uboldo	0.746	medium high	-0.243	medium low	0.321	medium high	0.244	low
35004	Bibbiano	0.036	medium low	-0.013	medium high	0.064	low	0.299	medium low
6052	Castelnuovo Bormida	1.560	high	-0.489	low	0.412	medium high	0.251	low
6054	Castelspina	1.635	high	-0.226	medium low	0.277	medium high	0.163	low
6006	Alluvioni Cambiù	1.925	high	-0.285	medium low	0.803	high	0.390	medium low

Munic code	Comune	log_Ae. caspius	quartile class	log_Ae. vexans	quartile class	prob_Ae. caspius	quartile class	prob_Ae. vexans	quartile class
6013	Bassignana	1.914	high	-0.175	medium low	0.723	high	0.567	medium high
6021	Bosco Marengo	1.793	high	-0.175	medium low	0.133	medium low	0.187	low
6060	Coniolo	2.112	high	-0.254	medium low	0.888	high	0.614	high
17145	Polpenazze del Garda	-0.861	low	-0.080	medium high	0.072	low	0.218	low
17146	Pompiano	-0.158	low	-0.032	medium high	0.101	medium low	0.649	high
35005	Boretto	0.868	medium high	1.681	high	0.295	medium high	0.675	high
6042	Cassano Spinola	2.113	high	-0.275	medium low	0.156	medium low	0.202	low
35006	Brescello	0.621	medium high	1.575	high	0.203	medium low	0.665	high
30043	Gemona del Friuli	0.000	low	0.000	medium high	0.008	low	0.062	low
6061	Conzano	2.192	high	0.096	medium high	0.413	medium high	0.268	low
6003	Alessandria	1.808	high	-0.148	medium high	0.336	medium high	0.285	medium low
30120	Terzo d'Aquileia	1.277	medium high	0.171	high	0.154	medium low	0.564	medium high
6008	Alzano Scrivia	2.308	high	-0.280	medium low	0.737	high	0.412	medium low
30122	Torreano	-0.679	low	-0.320	medium low	0.010	low	0.082	low
30083	Premariacco	-0.715	low	-0.328	medium low	0.021	low	0.340	medium low
29052	Porto Viro	2.300	high	0.557	high	0.758	high	0.775	high
30001	Aiello del Friuli	0.634	medium high	0.155	high	0.249	medium low	0.615	high
6043	Cassine	1.546	high	-0.445	medium low	0.406	medium high	0.248	low
6001	Acqui Terme	1.284	medium high	-0.627	low	0.243	medium low	0.159	low
17203	Visano	-0.535	low	0.039	medium high	0.044	low	0.614	high
5118	Villanova d'Asti	0.932	medium high	-0.186	medium low	0.128	medium low	0.306	medium low
18178	Villa Biscossi	2.252	high	-0.231	medium low	0.481	medium high	0.481	medium high
6019	Borgoratto Alessandrino	1.622	high	-0.157	medium high	0.471	medium high	0.242	low
6039	Casale Monferrato	2.202	high	-0.126	medium high	0.717	high	0.492	medium high
6037	Casal Cermelli	1.703	high	-0.157	medium high	0.441	medium high	0.294	medium low
6027	Camino	2.066	high	-0.231	medium low	0.774	high	0.332	medium low
18179	Villanova d'Ardenghi	2.246	high	-0.444	medium low	0.655	high	0.449	medium high
6051	Castelletto Monferrato	1.917	high	0.046	medium high	0.505	high	0.239	low
6023	Bozzole	2.104	high	0.041	medium high	0.899	high	0.666	high
6049	Castelletto d'Orba	1.669	high	-0.151	medium high	0.151	medium low	0.177	low
6053	Castelnuovo Scrivia	2.225	high	-0.246	medium low	0.464	medium high	0.354	medium low
6020	Borgo San Martino	2.243	high	0.094	medium high	0.715	high	0.537	medium high
6040	Casalnoceto	2.252	high	-0.100	medium high	0.157	medium low	0.175	low
16097	Fara Olivana con Sola	0.150	medium low	-0.415	medium low	0.142	medium low	0.476	medium high

Munic code	Comune	log_Ae. caspius	quartile class	log_Ae. vexans	quartile class	prob_Ae. caspius	quartile class	prob_Ae. vexans	quartile class
16098	Filago	-0.183	low	-0.666	low	0.120	medium low	0.271	medium low
15086	Cormano	1.363	medium high	-0.821	low	0.552	high	0.338	medium low
15087	Cornaredo	1.183	medium high	-0.469	low	0.630	high	0.354	medium low
29046	Taglio di Po	2.026	high	0.785	high	0.731	high	0.760	high
29047	Trecenta	1.367	medium high	-0.154	medium high	0.591	high	0.745	high
29048	Villadose	1.806	high	0.382	high	0.554	high	0.747	high
16044	Calcio	0.030	medium low	-0.239	medium low	0.118	medium low	0.540	medium high
16072	Chignolo d'Isola	-0.142	low	-0.584	low	0.277	medium high	0.203	low
16101	Fontanella	0.145	medium low	-0.231	medium low	0.179	medium low	0.565	medium high
15113	Inveruno	1.482	high	0.050	medium high	0.578	high	0.369	medium low
16073	Chiuduno	-0.368	low	-0.490	low	0.014	low	0.157	low
15237	Vignate	0.918	medium high	-1.059	low	0.762	high	0.475	medium high
15242	Vimodrone	1.294	medium high	-0.772	low	0.658	high	0.428	medium high
15243	Vittuone	1.305	medium high	-0.397	medium low	0.631	high	0.350	medium low
15244	Vizzolo Predabissi	1.507	high	-1.011	low	0.889	high	0.533	medium high
30082	Precenicco	1.286	medium high	0.259	high	0.546	high	0.579	high
15114	Inzago	0.172	medium low	-1.028	low	0.498	medium high	0.440	medium high
15115	Lacchiarella	1.809	high	-0.895	low	0.708	high	0.420	medium low
15116	Lainate	0.957	medium high	-0.410	medium low	0.490	medium high	0.307	medium low
15246	Zelo Surrigone	1.480	high	-0.703	low	0.704	high	0.454	medium high
16046	Calusco d'Adda	-0.014	low	-0.835	low	0.267	medium high	0.232	low
16047	Calvenzano	-0.049	low	-0.697	low	0.415	medium high	0.516	medium high
15146	Milano	1.362	medium high	-0.769	low	0.758	high	0.364	medium low
15150	Morimondo	1.699	high	-0.632	low	0.727	high	0.436	medium high
31020	San Lorenzo Isontino	0.092	medium low	0.065	medium high	0.058	low	0.510	medium high
31021	San Pier d'Isonzo	1.011	medium high	0.200	high	0.095	medium low	0.637	high
15205	Segrate	1.278	medium high	-0.745	low	0.710	high	0.465	medium high
15206	Senago	1.128	medium high	-0.598	low	0.606	high	0.323	medium low
15209	Sesto San Giovanni	1.401	medium high	-0.745	low	0.648	high	0.333	medium low
16049	Canonica d'Adda	-0.158	low	-0.757	low	0.328	medium high	0.462	medium high
15247	Zibido San Giacomo	1.636	high	-1.003	low	0.681	high	0.468	medium high
15248	Villa Cortese	1.218	medium high	0.027	medium high	0.433	medium high	0.298	medium low
15249	Vanzaghello	1.534	high	0.164	high	0.459	medium high	0.284	medium low
15250	Baranzate	1.234	medium high	-0.600	low	0.611	high	0.323	medium low
29049	Villamarzana	1.445	medium high	-0.192	medium low	0.417	medium high	0.728	high

Munic code	Comune	log_Ae. caspius	quartile class	log_Ae. vexans	quartile class	prob_Ae. caspius	quartile class	prob_Ae. vexans	quartile class
29050	Villanova del Ghebbo	1.019	medium high	-0.676	low	0.239	medium low	0.605	high
29051	Villanova Marchesana	2.082	high	1.117	high	0.784	high	0.751	high
16034	Bottanuco	-0.043	low	-0.749	low	0.270	medium high	0.272	medium low
15072	Cerro Maggiore	0.898	medium high	-0.228	medium low	0.407	medium high	0.293	medium low
15173	Pieve Emanuele	1.720	high	-0.924	low	0.817	high	0.486	medium high
15175	Pioltello	1.165	medium high	-0.917	low	0.667	high	0.476	medium high
16003	Albano Sant'Alessandro	-0.543	low	-0.644	low	0.046	low	0.183	low
16051	Capriate San Gervasio	-0.110	low	-0.693	low	0.327	medium high	0.346	medium low
15093	Corsico	1.396	medium high	-0.862	low	0.712	high	0.349	medium low
15096	Cuggiono	1.698	high	0.189	high	0.754	high	0.426	medium high
15188	Rosate	1.580	high	-0.930	low	0.626	high	0.463	medium high
16069	Cenate Sotto	-0.508	low	-0.625	low	0.043	low	0.168	low
15176	Pogliano Milanese	1.038	medium high	-0.324	medium low	0.582	high	0.331	medium low
15177	Pozzo d'Adda	0.073	medium low	-1.039	low	0.165	medium low	0.282	medium low
16079	Cologno al Serio	-0.097	low	-0.546	low	0.062	low	0.353	medium low
15189	Rozzano	1.602	high	-0.955	low	0.734	high	0.398	medium low
15191	San Colombano al Lambro	1.802	high	-0.130	medium high	0.516	high	0.458	medium high
15118	Legnano	1.022	medium high	-0.061	medium high	0.395	medium high	0.253	low
15122	Liscate	0.806	medium high	-1.093	low	0.764	high	0.487	medium high
15125	Locate di Triulzi	1.676	high	-0.902	low	0.663	high	0.454	medium high
16016	Azzano San Paolo	-0.403	low	-0.725	low	0.072	low	0.279	medium low
16081	Comun Nuovo	-0.276	low	-0.681	low	0.041	low	0.255	low
15151	Motta Visconti	1.989	high	-0.653	low	0.630	high	0.420	medium high
16024	Bergamo	-0.448	low	-0.681	low	0.103	medium low	0.193	low
15210	Settala	0.937	medium high	-1.005	low	0.765	high	0.506	medium high
15211	Settimo Milanese	1.261	medium high	-0.585	low	0.741	high	0.417	medium low
15213	Solaro	0.792	medium high	-0.588	low	0.566	high	0.269	medium low
15219	Trezzano Rosa	0.178	medium low	-1.024	low	0.194	medium low	0.253	low
16066	Cavernago	-0.346	low	-0.634	low	0.017	low	0.270	medium low
15154	Nerviano	1.015	medium high	-0.320	medium low	0.568	high	0.358	medium low
15155	Nosate	1.833	high	0.197	high	0.436	medium high	0.415	medium low
15157	Novate Milanese	1.323	medium high	-0.680	low	0.458	medium high	0.283	medium low
16083	Cortenuova	0.053	medium low	-0.312	medium low	0.129	medium low	0.481	medium high
16084	Costa di Mezzate	-0.505	low	-0.710	low	0.011	low	0.200	low
15097	Cusago	1.315	medium high	-0.625	low	0.876	high	0.479	medium high
15098	Cusano Milanino	1.383	medium high	-0.814	low	0.549	high	0.296	medium low

Munic code	Comune	log_Ae. caspius	quartile class	log_Ae. vexans	quartile class	prob_Ae. caspius	quartile class	prob_Ae. vexans	quartile class
16037	Brembate	-0.161	low	-0.699	low	0.223	medium low	0.338	medium low
16038	Brembate di Sopra	-0.350	low	-0.501	low	0.265	medium high	0.263	low
16089	Curno	-0.363	low	-0.589	low	0.112	medium low	0.261	low
15074	Cesano Boscone	1.372	medium high	-0.762	low	0.755	high	0.371	medium low
15076	Cesate	0.917	medium high	-0.599	low	0.577	high	0.283	medium low
15099	Dairago	1.321	medium high	0.088	medium high	0.310	medium high	0.315	medium low
15101	Dresano	1.376	medium high	-0.992	low	0.604	high	0.451	medium high
15103	Gaggiano	1.439	medium high	-0.786	low	0.654	high	0.467	medium high
16091	Dalmine	-0.285	low	-0.631	low	0.120	medium low	0.296	medium low
15077	Cinisello Balsamo	1.382	medium high	-0.766	low	0.493	medium high	0.315	medium low
15078	Cisliano	1.352	medium high	-0.542	low	0.608	high	0.421	medium high
15178	Pozzuolo Martesana	0.349	medium low	-1.067	low	0.758	high	0.533	medium high
15179	Pregnana Milanese	1.120	medium high	-0.406	medium low	0.631	high	0.380	medium low
16053	Caravaggio	0.069	medium low	-0.547	low	0.199	medium low	0.468	medium high
16055	Carobbio degli Angeli	-0.488	low	-0.618	low	0.013	low	0.146	low
15035	Bubbiano	1.699	high	-0.842	low	0.954	high	0.529	medium high
15192	San Donato Milanese	1.446	medium high	-0.869	low	0.837	high	0.501	medium high
15194	San Giorgio su Legnano	1.113	medium high	-0.033	medium high	0.359	medium high	0.232	low
15195	San Giuliano Milanese	1.514	high	-0.902	low	0.794	high	0.507	medium high
15130	Magenta	1.587	high	0.001	medium high	0.696	high	0.420	medium low
15131	Magnago	1.459	medium high	0.135	high	0.368	medium high	0.279	medium low
15134	Marcallo con Casone	1.504	high	-0.046	medium high	0.602	high	0.401	medium low
15136	Masate	0.305	medium low	-1.066	low	0.384	medium high	0.418	medium low
15181	Rescaldina	0.760	medium high	-0.191	medium low	0.477	medium high	0.269	medium low
15182	Rho	1.118	medium high	-0.493	low	0.641	high	0.357	medium low
15036	Buccinasco	1.485	high	-0.908	low	0.784	high	0.475	medium high
15038	Buscate	1.537	high	0.123	high	0.540	high	0.352	medium low
15040	Bussero	0.954	medium high	-1.073	low	0.755	high	0.436	medium high
15041	Busto Garolfo	1.282	medium high	0.018	medium high	0.463	medium high	0.342	medium low
16028	Bolgare	-0.361	low	-0.562	low	0.014	low	0.269	low
16029	Boltiere	-0.226	low	-0.719	low	0.209	medium low	0.347	medium low
15220	Trezzano sul Naviglio	1.409	medium high	-0.786	low	0.786	high	0.412	medium low
15221	Trezzo sull'Adda	0.034	medium low	-0.876	low	0.325	medium high	0.330	medium low
15222	Tribiano	1.240	medium high	-0.994	low	0.673	high	0.489	medium high
15224	Truccazzano	0.423	medium low	-1.089	low	0.689	high	0.544	medium high

Munic code	Comune	log_Ae. caspius	quartile class	log_Ae. vexans	quartile class	prob_Ae. caspius	quartile class	prob_Ae. vexans	quartile class
16018	Bagnatica	-0.500	low	-0.736	low	0.008	low	0.203	low
16019	Barbata	0.201	medium low	-0.307	medium low	0.131	medium low	0.495	medium high
16020	Bariano	0.148	medium low	-0.432	medium low	0.111	medium low	0.381	medium low
15158	Noviglio	1.594	high	-0.976	low	0.431	medium high	0.369	medium low
15159	Opera	1.626	high	-0.942	low	0.800	high	0.500	medium high
15164	Ossona	1.382	medium high	-0.191	medium low	0.580	high	0.385	medium low
15165	Ozzero	1.664	high	-0.413	medium low	0.610	high	0.389	medium low
16087	Covo	0.084	medium low	-0.341	medium low	0.130	medium low	0.503	medium high
16040	Brignano Gera d'Adda	-0.125	low	-0.629	low	0.099	medium low	0.360	medium low
16075	Ciserano	-0.239	low	-0.726	low	0.158	medium low	0.364	medium low
16076	Cividate al Piano	0.015	low	-0.269	medium low	0.063	low	0.425	medium high
15081	Cologno Monzese	1.334	medium high	-0.797	low	0.557	high	0.380	medium low
16005	Almè	-0.425	low	-0.439	medium low	0.215	medium low	0.221	low
15105	Garbagnate Milanese	1.057	medium high	-0.597	low	0.717	high	0.326	medium low
15106	Gessate	0.501	medium low	-1.088	low	0.430	medium high	0.366	medium low
15108	Gorgonzola	0.679	medium high	-1.060	low	0.665	high	0.458	medium high
15110	Grezzago	0.099	medium low	-1.001	low	0.174	medium low	0.279	medium low
15112	Gudo Visconti	1.481	high	-0.803	low	0.723	high	0.464	medium high
15139	Mediglia	1.307	medium high	-0.993	low	0.760	high	0.524	medium high
15140	Melegnano	1.541	high	-0.988	low	0.782	high	0.420	medium low
15082	Colturano	1.426	medium high	-0.992	low	0.787	high	0.572	medium high
15085	Corbetta	1.387	medium high	-0.323	medium low	0.692	high	0.414	medium low
15183	Robecchetto con Induno	1.807	high	0.224	high	0.648	high	0.414	medium low
30075	Pocenia	0.689	medium high	0.261	high	0.495	medium high	0.659	high
30077	Porpetto	0.613	medium high	0.202	high	0.361	medium high	0.628	high
15200	Santo Stefano Ticino	1.373	medium high	-0.257	medium low	0.778	high	0.414	medium low
15201	San Vittore Olona	0.984	medium high	-0.115	medium high	0.581	high	0.325	medium low
15202	San Zenone al Lambro	1.602	high	-0.918	low	0.554	high	0.484	medium high
15204	Sedriano	1.258	medium high	-0.435	medium low	0.731	high	0.393	medium low
15142	Melzo	0.634	medium high	-1.089	low	0.712	high	0.439	medium high
15144	Mesero	1.539	high	0.053	medium high	0.597	high	0.415	medium low
15226	Turbigo	1.884	high	0.230	high	0.699	high	0.385	medium low
15229	Vanzago	1.127	medium high	-0.360	medium low	0.642	high	0.436	medium high
15230	Vaprio d'Adda	-0.060	low	-0.873	low	0.275	medium high	0.356	medium low

Munic code	Comune	log_Ae. caspius	quartile class	log_Ae. vexans	quartile class	prob_Ae. caspius	quartile class	prob_Ae. vexans	quartile class
15184	Robecco sul Naviglio	1.611	high	-0.041	medium high	0.668	high	0.466	medium high
15185	Rodano	1.052	medium high	-0.952	low	0.858	high	0.557	medium high
16021	Barzana	-0.352	low	-0.507	low	0.276	medium high	0.218	low
30078	Povoletto	-0.426	low	-0.099	medium high	0.031	low	0.273	medium low
30079	Pozzuolo del Friuli	-0.220	low	-0.108	medium high	0.096	medium low	0.452	medium high
30080	Pradamano	-0.417	low	-0.228	medium low	0.042	low	0.341	medium low
16030	Bonate Sopra	-0.251	low	-0.544	low	0.149	medium low	0.236	low
16031	Bonate Sotto	-0.225	low	-0.559	low	0.087	medium low	0.242	low
15235	Vermezzo	1.455	medium high	-0.631	low	0.855	high	0.444	medium high
15236	Vernate	1.725	high	-0.883	low	0.368	medium high	0.321	medium low
16042	Brusaporto	-0.526	low	-0.760	low	0.024	low	0.214	low
16043	Calcinate	-0.327	low	-0.584	low	0.018	low	0.266	low
16096	Fara Gera d'Adda	-0.212	low	-0.827	low	0.434	medium high	0.490	medium high
16122	Isso	0.177	medium low	-0.404	medium low	0.141	medium low	0.494	medium high
16123	Lallio	-0.351	low	-0.646	low	0.118	medium low	0.290	medium low
15055	Casarile	1.775	high	-0.883	low	0.538	high	0.405	medium low
15058	Casorezzo	1.271	medium high	-0.152	medium high	0.585	high	0.424	medium high
15059	Cassano d'Adda	-0.074	low	-1.018	low	0.506	high	0.480	medium high
15060	Cassina de' Pecchi	0.924	medium high	-1.035	low	0.699	high	0.437	medium high
15061	Cassinetta di Lugagnano	1.516	high	-0.220	medium low	0.679	high	0.461	medium high
16131	Madone	-0.192	low	-0.652	low	0.097	medium low	0.198	low
16239	Villa d'Almè	-0.418	low	-0.483	low	0.119	medium low	0.078	low
16240	Villa di Serio	-0.491	low	-0.523	low	0.067	low	0.146	low
15170	Pero	1.214	medium high	-0.591	low	0.617	high	0.318	medium low
15171	Peschiera Borromeo	1.276	medium high	-0.841	low	0.820	high	0.523	medium high
15172	Pessano con Bornago	0.815	medium high	-1.115	low	0.476	medium high	0.403	medium low
16013	Arzago d'Adda	0.057	medium low	-0.901	low	0.329	medium high	0.484	medium high
16132	Mapello	-0.255	low	-0.511	low	0.146	medium low	0.160	low
16133	Martinengo	-0.029	low	-0.415	medium low	0.087	medium low	0.385	medium low
16177	Pumenengo	-0.009	low	-0.151	medium high	0.143	medium low	0.581	high
16178	Ranica	-0.488	low	-0.699	low	0.049	low	0.089	low
16189	San Paolo d'Argon	-0.546	low	-0.713	low	0.029	low	0.175	low
16062	Castelli Calepio	-0.141	low	-0.092	medium high	0.046	low	0.252	low
16063	Castel Rozzone	-0.198	low	-0.700	low	0.172	medium low	0.363	medium low
17020	Borgo San Giacomo	-0.014	low	0.149	high	0.102	medium low	0.624	high
17021	Borgosatollo	-0.593	low	-0.358	medium low	0.056	low	0.609	high

Munic code	Comune	log_Ae. caspius	quartile class	log_Ae. vexans	quartile class	prob_Ae. caspius	quartile class	prob_Ae. vexans	quartile class
15062	Castano Primo	1.706	high	0.179	high	0.627	high	0.351	medium low
15070	Cernusco sul Naviglio	1.141	medium high	-0.991	low	0.653	high	0.393	medium low
15071	Cerro al Lambro	1.642	high	-0.981	low	0.672	high	0.486	medium high
16135	Misano di Gera d'Adda	0.127	medium low	-0.588	low	0.299	medium high	0.541	medium high
16160	Pedrengo	-0.522	low	-0.747	low	0.063	low	0.265	low
17023	Botticino	-0.665	low	-0.021	medium high	0.037	low	0.082	low
16169	Ponteranica	-0.477	low	-0.002	medium high	0.056	low	0.041	low
16170	Ponte San Pietro	-0.333	low	-0.529	low	0.177	medium low	0.270	medium low
16183	Romano di Lombardia	0.103	medium low	-0.407	medium low	0.117	medium low	0.420	medium low
16245	Zanica	-0.354	low	-0.704	low	0.047	low	0.286	medium low
17004	Alfianello	-0.151	low	0.497	high	0.030	low	0.584	high
17013	Bassano Bresciano	-0.275	low	0.124	high	0.013	low	0.536	medium high
17014	Bedizzole	-0.902	low	-0.080	medium high	0.020	low	0.300	medium low
16206	Spirano	-0.204	low	-0.669	low	0.022	low	0.248	low
16207	Stezzano	-0.341	low	-0.685	low	0.090	medium low	0.285	medium low
16209	Suisio	-0.038	low	-0.802	low	0.424	medium high	0.326	medium low
16150	Orio al Serio	-0.454	low	-0.743	low	0.040	low	0.305	medium low
17015	Berlingo	-0.103	low	0.114	medium high	0.145	medium low	0.582	high
16114	Gorlago	-0.539	low	-0.687	low	0.011	low	0.166	low
16115	Gorle	-0.500	low	-0.731	low	0.081	medium low	0.256	low
16194	Scanzorosciate	-0.515	low	-0.729	low	0.054	low	0.154	low
16218	Trescore Balneario	-0.470	low	-0.437	medium low	0.015	low	0.094	low
16139	Montello	-0.561	low	-0.620	low	0.014	low	0.191	low
16140	Morengo	0.071	medium low	-0.475	low	0.081	medium low	0.394	medium low
16141	Mornico al Serio	-0.132	low	-0.432	medium low	0.041	low	0.351	medium low
16219	Treviglio	-0.222	low	-0.755	low	0.276	medium high	0.441	medium high
16105	Fornovo San Giovanni	0.151	medium low	-0.452	medium low	0.139	medium low	0.468	medium high
16006	Almenno San Bartolomeo	-0.378	low	-0.461	low	0.194	medium low	0.133	low
16007	Almenno San Salvatore	-0.396	low	-0.463	low	0.203	medium low	0.134	low
16126	Levate	-0.296	low	-0.682	low	0.091	medium low	0.300	medium low
16172	Pontirolo Nuovo	-0.212	low	-0.757	low	0.264	medium high	0.447	medium high
16057	Carvico	-0.064	low	-0.826	low	0.388	medium high	0.155	low
15042	Calvignasco	1.693	high	-0.877	low	0.873	high	0.396	medium low
16232	Verdellino	-0.266	low	-0.715	low	0.104	medium low	0.258	low
16233	Verdello	-0.271	low	-0.713	low	0.048	low	0.255	low
16212	Telgate	-0.236	low	-0.367	medium low	0.073	low	0.385	medium low
16117	Grassobbio	-0.432	low	-0.733	low	0.050	low	0.273	medium low

Munic code	Comune	log_Ae. caspius	quartile class	log_Ae. vexans	quartile class	prob_Ae. caspius	quartile class	prob_Ae. vexans	quartile class
15044	Cambiago	0.563	medium high	-1.081	low	0.380	medium high	0.367	medium low
15046	Canegrate	1.064	medium high	-0.059	medium high	0.432	medium high	0.273	medium low
15050	Carpiano	1.669	high	-0.908	low	0.679	high	0.464	medium high
15051	Carugate	1.107	medium high	-1.089	low	0.602	high	0.385	medium low
16220	Treviolo	-0.333	low	-0.605	low	0.098	medium low	0.274	medium low
16142	Mozzanica	0.237	medium low	-0.434	medium low	0.189	medium low	0.471	medium high
16143	Mozzo	-0.391	low	-0.563	low	0.238	medium low	0.208	low
16167	Pognano	-0.228	low	-0.695	low	0.049	low	0.296	medium low
15166	Paderno Dugnano	1.262	medium high	-0.715	low	0.577	high	0.306	medium low
15167	Pantigliate	1.096	medium high	-0.979	low	0.795	high	0.467	medium high
15168	Parabiago	1.080	medium high	-0.215	medium low	0.503	medium high	0.302	medium low
15169	Paullo	1.045	medium high	-0.994	low	0.753	high	0.535	medium high
16222	Urgnano	-0.256	low	-0.640	low	0.027	low	0.249	low
16224	Valbrembo	-0.405	low	-0.500	low	0.218	medium low	0.232	low
16250	Medolago	-0.042	low	-0.778	low	0.264	medium high	0.231	low
16251	Solza	0.010	low	-0.846	low	0.304	medium high	0.195	low
17001	Acquafredda	-0.534	low	0.077	medium high	0.121	medium low	0.655	high
16008	Alzano Lombardo	-0.481	low	-0.683	low	0.030	low	0.039	low
16009	Ambivere	-0.301	low	-0.492	low	0.133	medium low	0.125	low
16010	Antegnate	0.086	medium low	-0.325	medium low	0.107	medium low	0.473	medium high
16011	Arcene	-0.230	low	-0.717	low	0.155	medium low	0.377	medium low
16129	Lurano	-0.191	low	-0.678	low	0.048	low	0.240	low
17002	Adro	0.028	medium low	0.319	high	0.061	low	0.214	low
16213	Terno d'Isola	-0.155	low	-0.551	low	0.292	medium high	0.235	low
16214	Torre Boldone	-0.494	low	-0.708	low	0.099	medium low	0.122	low
16059	Casirate d'Adda	-0.142	low	-0.907	low	0.513	high	0.540	medium high
16176	Presezzo	-0.288	low	-0.531	low	0.124	medium low	0.247	low
17008	Azzano Mella	-0.348	low	-0.141	medium high	0.068	low	0.563	medium high
17009	Bagnolo Mella	-0.537	low	-0.548	low	0.020	low	0.542	medium high
17011	Barbariga	-0.248	low	-0.112	medium high	0.062	low	0.577	high
16216	Torre de' Roveri	-0.536	low	-0.743	low	0.042	low	0.163	low
16217	Torre Pallavicina	-0.001	low	-0.131	medium high	0.154	medium low	0.577	high
16120	Grumello del Monte	-0.214	low	-0.240	medium low	0.019	low	0.168	low
20001	Acquanegra sul Chiese	0.094	medium low	0.200	high	0.083	medium low	0.509	medium high
19095	Sesto ed Uniti	0.259	medium low	0.609	high	0.097	medium low	0.627	high
19096	Solarolo Rainerio	0.317	medium low	1.105	high	0.053	low	0.606	high

Munic code	Comune	log_Ae. caspius	quartile class	log_Ae. vexans	quartile class	prob_Ae. caspius	quartile class	prob_Ae. vexans	quartile class
18122	Rivanazzano Terme	2.275	high	-0.096	medium high	0.136	medium low	0.157	low
19065	Paderno Ponchielli	0.233	medium low	0.479	high	0.036	low	0.510	medium high
19066	Palazzo Pignano	0.524	medium low	-0.596	low	0.359	medium high	0.567	medium high
19067	Pandino	0.566	medium high	-0.776	low	0.370	medium high	0.536	medium high
18113	Pieve del Cairo	2.208	high	-0.281	medium low	0.738	high	0.568	medium high
19068	Persico Dosimo	-0.055	low	0.863	high	0.029	low	0.550	medium high
16156	Palazzago	-0.317	low	-0.489	low	0.084	medium low	0.056	low
16157	Palosco	-0.100	low	-0.326	medium low	0.063	low	0.394	medium low
18123	Robbio	2.055	high	-0.362	medium low	0.363	medium high	0.400	medium low
18124	Robecco Pavese	2.022	high	-0.523	low	0.382	medium high	0.405	medium low
20021	Curtatone	0.888	medium high	0.744	high	0.572	high	0.701	high
19069	Pescarolo ed Uniti	0.203	medium low	1.221	high	0.009	low	0.521	medium high
19070	Pessina Cremonese	0.304	medium low	1.260	high	0.009	low	0.481	medium high
19071	Piadena	0.181	medium low	0.661	high	0.084	medium low	0.563	medium high
19109	Trescore Cremasco	0.480	medium low	-0.526	low	0.367	medium high	0.528	medium high
16244	Zandobbio	-0.414	low	-0.301	medium low	0.012	low	0.057	low
18168	Valle Lomellina	2.161	high	-0.300	medium low	0.402	medium high	0.406	medium low
18169	Valle Salimbene	2.231	high	-0.559	low	0.604	high	0.492	medium high
18135	San Genesio ed Uniti	2.125	high	-0.597	low	0.682	high	0.470	medium high
18136	San Giorgio di Lomellina	2.427	high	-0.250	medium low	0.406	medium high	0.343	medium low
19110	Trigolo	0.375	medium low	0.074	medium high	0.054	low	0.499	medium high
19111	Vaiano Cremasco	0.710	medium high	-0.501	low	0.393	medium high	0.532	medium high
19112	Vailate	0.184	medium low	-0.718	low	0.252	medium high	0.443	medium high
19113	Vescovato	0.129	medium low	1.350	high	0.005	low	0.455	medium high
19114	Volongo	0.116	medium low	0.915	high	0.008	low	0.483	medium high
16203	Sotto il Monte Giovanni XXIII	-0.145	low	-0.651	low	0.283	medium high	0.125	low
19048	Gerre de' Caprioli	0.007	low	1.049	high	0.108	medium low	0.785	high
19049	Gombito	0.808	medium high	0.115	medium high	0.442	medium high	0.653	high
19050	Grontardo	0.038	medium low	1.026	high	0.015	low	0.568	medium high
18190	Zinasco	2.375	high	-0.345	medium low	0.678	high	0.570	medium high
18137	San Martino Siccomario	2.419	high	-0.478	low	0.676	high	0.528	medium high
18138	Sannazzaro de' Burgondi	2.484	high	-0.241	medium low	0.658	high	0.488	medium high
16113	Ghisalba	-0.198	low	-0.532	low	0.053	low	0.325	medium low
19051	Grumello Cremonese ed Uniti	0.480	medium low	0.623	high	0.093	medium low	0.553	medium high

Munic code	Comune	log_Ae. caspius	quartile class	log_Ae. vexans	quartile class	prob_Ae. caspius	quartile class	prob_Ae. vexans	quartile class
19052	Gussola	0.420	medium low	1.345	high	0.134	medium low	0.765	high
20004	Bigarello	1.684	high	0.097	medium high	0.735	high	0.727	high
15032	Bresso	1.431	medium high	-0.824	low	0.675	high	0.417	medium low
19022	Casalmorano	0.262	medium low	0.251	high	0.091	medium low	0.565	medium high
19023	Casteldidone	0.320	medium low	0.834	high	0.073	low	0.566	medium high
19090	San Giovanni in Croce	0.293	medium low	0.904	high	0.105	medium low	0.623	high
19091	San Martino del Lago	0.285	medium low	1.287	high	0.079	medium low	0.684	high
18189	Zerbolù	2.224	high	-0.500	low	0.629	high	0.507	medium high
19001	Acquanegra Cremonese	0.420	medium low	0.658	high	0.196	medium low	0.672	high
18159	Torre d'Isola	2.155	high	-0.599	low	0.810	high	0.479	medium high
18160	Torrevecchia Pia	1.913	high	-0.792	low	0.694	high	0.492	medium high
18161	Torricella Verzate	1.912	high	-0.289	medium low	0.172	medium low	0.224	low
18127	Rognano	1.858	high	-0.841	low	0.563	high	0.446	medium high
19092	Scandolara Ravara	0.308	medium low	1.375	high	0.072	low	0.725	high
19093	Scandolara Ripa d'Oglio	0.019	low	0.845	high	0.029	low	0.662	high
18102	Mortara	2.189	high	-0.260	medium low	0.481	medium high	0.318	medium low
19072	Pianengo	0.304	medium low	-0.210	medium low	0.268	medium high	0.579	high
19073	Pieranica	0.352	medium low	-0.675	low	0.158	medium low	0.458	medium high
19074	Pieve d'Olmi	0.006	low	1.443	high	0.056	low	0.719	high
19075	Pieve San Giacomo	0.172	medium low	1.531	high	0.003	low	0.434	medium high
19076	Pizzighettone	0.686	medium high	0.654	high	0.262	medium high	0.643	high
19036	Cremona	-0.071	low	0.845	high	0.115	medium low	0.650	high
19037	Cremosano	0.472	medium low	-0.406	medium low	0.231	medium low	0.553	medium high
19038	Crotta d'Adda	0.546	medium low	0.734	high	0.277	medium high	0.736	high
18149	Silvano Pietra	2.477	high	-0.231	medium low	0.675	high	0.401	medium low
18150	Siziano	1.835	high	-0.833	low	0.789	high	0.462	medium high
18151	Sommo	2.361	high	-0.424	medium low	0.761	high	0.643	high
18152	Spessa	1.666	high	-0.422	medium low	0.584	high	0.670	high
18066	Galliavola	2.337	high	-0.248	medium low	0.580	high	0.520	medium high
18067	Gambarana	2.044	high	-0.235	medium low	0.570	high	0.600	high
19039	Cumignano sul Naviglio	0.232	medium low	0.147	high	0.042	low	0.444	medium high
20002	Asola	-0.106	low	0.295	high	0.027	low	0.471	medium high
18172	Velezzo Lomellina	2.258	high	-0.273	medium low	0.186	medium low	0.313	medium low
19053	Isola Dovarese	0.202	medium low	1.056	high	0.037	low	0.557	medium high
19054	Izano	0.390	medium low	-0.030	medium high	0.391	medium high	0.633	high

Munic code	Comune	log_Ae. caspius	quartile class	log_Ae. vexans	quartile class	prob_Ae. caspius	quartile class	prob_Ae. vexans	quartile class
19055	Madignano	0.556	medium high	-0.217	medium low	0.305	medium high	0.585	high
19024	Castel Gabbiano	0.177	medium low	-0.440	medium low	0.117	medium low	0.484	medium high
19025	Castelleone	0.586	medium high	0.007	medium high	0.208	medium low	0.559	medium high
19026	Castelverde	0.029	medium low	0.606	high	0.030	low	0.512	medium high
18139	Santa Cristina e Bissone	1.830	high	-0.236	medium low	0.619	high	0.616	high
18140	Santa Giuletta	1.835	high	-0.542	low	0.296	medium high	0.314	medium low
19002	Agnadello	0.323	medium low	-0.945	low	0.267	medium high	0.506	medium high
20003	Bagnolo San Vito	1.240	medium high	0.977	high	0.645	high	0.739	high
18114	Pieve Porto Morone	1.659	high	0.015	medium high	0.765	high	0.697	high
19056	Malagnino	0.005	low	1.234	high	0.014	low	0.521	medium high
19057	Martignana di Po	0.476	medium low	1.265	high	0.146	medium low	0.784	high
19104	Ticengo	0.181	medium low	0.030	medium high	0.063	low	0.479	medium high
19105	Torlino Vimercati	0.371	medium low	-0.719	low	0.163	medium low	0.495	medium high
19106	Tornata	0.188	medium low	0.398	high	0.069	low	0.577	high
19100	Spinadesco	0.326	medium low	0.690	high	0.211	medium low	0.789	high
19101	Spineda	0.315	medium low	0.368	high	0.038	low	0.456	medium high
19102	Spino d'Adda	0.824	medium high	-0.858	low	0.499	medium high	0.549	medium high
19103	Stagno Lombardo	-0.014	low	1.270	high	0.081	medium low	0.767	high
18118	Portalbera	1.520	high	-0.498	low	0.568	high	0.559	medium high
18119	Rea	2.398	high	-0.509	low	0.783	high	0.590	high
19060	Moscazzano	0.900	medium high	-0.060	medium high	0.450	medium high	0.628	high
19061	Motta Baluffi	0.171	medium low	1.584	high	0.057	low	0.725	high
19062	Offanengo	0.314	medium low	-0.151	medium high	0.144	medium low	0.479	medium high
19063	Olmeneta	-0.054	low	0.523	high	0.018	low	0.513	medium high
19097	Soncino	0.105	medium low	0.127	high	0.134	medium low	0.567	medium high
19032	Corte de' Cortesi con Cignone	-0.039	low	0.377	high	0.067	low	0.637	high
20030	Mantova	1.409	medium high	0.588	high	0.862	high	0.686	high
16153	Osio Sotto	-0.243	low	-0.702	low	0.191	medium low	0.343	medium low
16154	Pagazzano	-0.022	low	-0.550	low	0.167	medium low	0.432	medium high
16155	Paladina	-0.410	low	-0.502	low	0.245	medium low	0.241	low
18120	Redavalle	1.681	high	-0.662	low	0.380	medium high	0.302	medium low
18121	Retorbido	2.267	high	-0.032	medium high	0.071	low	0.118	low
18165	Trovo	1.902	high	-0.788	low	0.547	high	0.373	medium low
19098	Soresina	0.380	medium low	0.219	high	0.100	medium low	0.514	medium high

Munic code	Comune	log_Ae. caspius	quartile class	log_Ae. vexans	quartile class	prob_Ae. caspius	quartile class	prob_Ae. vexans	quartile class
19099	Sospiro	0.080	medium low	1.490	high	0.014	low	0.544	medium high
18112	Pieve Albignola	2.475	high	-0.286	medium low	0.615	high	0.586	high
18155	Torrazza Coste	2.171	high	-0.114	medium high	0.155	medium low	0.167	low
16202	Sorisole	-0.442	low	0.000	medium high	0.097	medium low	0.079	low
18167	Valeggio	2.569	high	-0.232	medium low	0.411	medium high	0.404	medium low
19044	Formigara	0.776	medium high	0.299	high	0.354	medium high	0.690	high
18133	San Cipriano Po	1.707	high	-0.574	low	0.611	high	0.644	high
18134	San Damiano al Colle	1.283	medium high	-0.218	medium low	0.257	medium high	0.267	low
18156	Torre Beretti e Castellaro	2.064	high	-0.062	medium high	0.624	high	0.585	high
18157	Torre d'Arese	1.958	high	-0.598	low	0.608	high	0.459	medium high
18158	Torre de' Negri	1.812	high	-0.410	medium low	0.745	high	0.622	high
19033	Corte de' Frati	-0.054	low	0.729	high	0.027	low	0.562	medium high
17025	Bovezzo	-0.364	low	0.058	medium high	0.022	low	0.046	low
19045	Gabbioneta-Binanuova	0.148	medium low	0.977	high	0.017	low	0.568	medium high
19046	Gadesco-Pieve Delmona	0.012	low	1.118	high	0.020	low	0.529	medium high
19047	Genivolta	0.254	medium low	0.196	high	0.049	low	0.488	medium high
19094	Sergnano	0.300	medium low	-0.298	medium low	0.170	medium low	0.484	medium high
18177	Vigevano	2.031	high	-0.248	medium low	0.612	high	0.438	medium high
19064	Ostiano	0.085	medium low	0.863	high	0.004	low	0.418	medium low
19034	Credera Rubbiano	0.971	medium high	-0.135	medium high	0.352	medium high	0.580	high
19035	Crema	0.514	medium low	-0.226	medium low	0.348	medium high	0.559	medium high
19115	Voltido	0.267	medium low	1.062	high	0.029	low	0.576	high
20057	San Giorgio di Mantova	1.653	high	0.255	high	0.705	high	0.623	high
20068	Villimpenta	1.449	medium high	0.288	high	0.480	medium high	0.561	medium high
41019	Gabicce Mare	0.000	low	0.000	medium high	0.003	low	0.282	medium low
20070	Volta Mantovana	-0.184	low	-0.395	medium low	0.598	high	0.634	high
20058	San Giovanni del Dosso	1.305	medium high	0.708	high	0.463	medium high	0.637	high
20059	San Martino dall'Argine	0.246	medium low	0.084	medium high	0.163	medium low	0.558	medium high
20060	Schivenoglia	1.115	medium high	0.536	high	0.593	high	0.710	high
41020	Gradara	0.000	low	0.000	medium high	0.002	low	0.323	medium low
20061	Sermide	1.180	medium high	0.387	high	0.611	high	0.721	high
20062	Serravalle a Po	1.175	medium high	0.349	high	0.736	high	0.812	high
20063	Solferino	-0.859	low	-0.224	medium low	0.334	medium high	0.491	medium high
20064	Sustinente	1.256	medium high	0.486	high	0.736	high	0.774	high
20022	Dosolo	0.900	medium high	1.371	high	0.546	high	0.795	high

Munic code	Comune	log_Ae. caspius	quartile class	log_Ae. vexans	quartile class	prob_Ae. caspius	quartile class	prob_Ae. vexans	quartile class
20023	Felonica	1.263	medium high	0.413	high	0.614	high	0.810	high
20024	Gazoldo degli Ippoliti	0.158	medium low	0.080	medium high	0.396	medium high	0.625	high
20025	Gazzuolo	0.391	medium low	0.355	high	0.269	medium high	0.634	high
20033	Marmirolo	0.811	medium high	-0.133	medium high	0.598	high	0.672	high
20034	Medole	-0.682	low	-0.153	medium high	0.174	medium low	0.516	medium high
20043	Pomponesco	0.880	medium high	1.502	high	0.467	medium high	0.735	high
18185	Zeccone	2.012	high	-0.669	low	0.493	medium high	0.383	medium low
20045	Porto Mantovano	1.178	medium high	0.275	high	0.788	high	0.734	high
20046	Quingentole	1.143	medium high	0.541	high	0.631	high	0.788	high
18099	Monticelli Pavese	1.554	high	0.257	high	0.672	high	0.718	high
18181	Vistarino	2.013	high	-0.541	low	0.699	high	0.496	medium high
18182	Voghera	2.394	high	-0.163	medium high	0.297	medium high	0.322	medium low
18186	Zeme	2.098	high	-0.442	medium low	0.457	medium high	0.421	medium high
20035	Moglia	1.650	high	1.324	high	0.551	high	0.620	high
18077	Inverno e Monteleone	1.942	high	-0.441	medium low	0.616	high	0.539	medium high
20014	Castel d'Ario	1.586	high	0.064	medium high	0.739	high	0.671	high
20015	Castel Goffredo	-0.501	low	0.049	medium high	0.172	medium low	0.570	medium high
18188	Zerbo	1.639	high	-0.167	medium high	0.596	high	0.638	high
20006	Borgofranco sul Po	1.049	medium high	0.188	high	0.806	high	0.781	high
20047	Quistello	1.348	medium high	0.911	high	0.495	medium high	0.652	high
20048	Redondesco	0.111	medium low	0.080	medium high	0.227	medium low	0.604	high
15012	Bareggio	1.248	medium high	-0.491	low	0.764	high	0.434	medium high
15014	Basiano	0.287	medium low	-1.041	low	0.439	medium high	0.336	medium low
15015	Basiglio	1.728	high	-0.963	low	0.783	high	0.439	medium high
15016	Bellinzago Lombardo	0.424	medium low	-1.043	low	0.471	medium high	0.460	medium high
15019	Bernate Ticino	1.701	high	0.194	high	0.810	high	0.479	medium high
18088	Mede	2.168	high	-0.177	medium low	0.665	high	0.477	medium high
18103	Nicorvo	2.047	high	-0.344	medium low	0.270	medium high	0.376	medium low
18104	Olevano di Lomellina	2.187	high	-0.367	medium low	0.345	medium high	0.309	medium low
18078	Landriano	1.825	high	-0.860	low	0.765	high	0.492	medium high
18079	Langosco	2.070	high	-0.449	medium low	0.440	medium high	0.479	medium high
18080	Lardirago	2.088	high	-0.663	low	0.528	high	0.515	medium high
18091	Mezzana Rabattone	2.379	high	-0.339	medium low	0.608	high	0.656	high
18092	Mezzanino	2.232	high	-0.574	low	0.729	high	0.515	medium high
20026	Goito	0.262	medium low	-0.128	medium high	0.578	high	0.702	high

Munic code	Comune	log_Ae. caspius	quartile class	log_Ae. vexans	quartile class	prob_Ae. caspius	quartile class	prob_Ae. vexans	quartile class
20037	Motteggiana	0.972	medium high	1.234	high	0.534	high	0.754	high
20038	Ostiglia	1.099	medium high	0.180	high	0.774	high	0.730	high
20016	Castellucchio	0.478	medium low	0.379	high	0.229	medium low	0.582	high
20017	Castiglione delle Stiviere	-0.870	low	-0.182	medium low	0.182	medium low	0.525	medium high
20027	Gonzaga	1.488	high	1.503	high	0.566	high	0.609	high
15022	Besate	1.899	high	-0.635	low	0.507	high	0.393	medium low
15024	Binasco	1.727	high	-0.932	low	0.473	medium high	0.328	medium low
15026	Boffalora sopra Ticino	1.668	high	0.174	high	0.805	high	0.480	medium high
15027	Bollate	1.214	medium high	-0.603	low	0.625	high	0.356	medium low
18093	Miradolo Terme	1.878	high	-0.256	medium low	0.536	high	0.463	medium high
18106	Ottobiano	2.519	high	-0.235	medium low	0.600	high	0.353	medium low
18107	Palestro	2.032	high	-0.381	medium low	0.524	high	0.475	medium high
18108	Pancarana	2.363	high	-0.342	medium low	0.593	high	0.443	medium high
18109	Parona	2.112	high	-0.148	medium high	0.659	high	0.391	medium low
18081	Linarolo	2.071	high	-0.547	low	0.727	high	0.591	high
18083	Lomello	2.344	high	-0.237	medium low	0.547	high	0.492	medium high
18084	Lungavilla	2.249	high	-0.348	medium low	0.435	medium high	0.463	medium high
18095	Montebello della Battaglia	2.151	high	-0.241	medium low	0.229	medium low	0.296	medium low
20028	Guidizzolo	-0.513	low	-0.188	medium low	0.278	medium high	0.503	medium high
20039	Pegognaga	1.275	medium high	1.392	high	0.535	high	0.654	high
20040	Pieve di Coriano	1.058	medium high	0.344	high	0.614	high	0.776	high
20041	Piubega	-0.051	low	0.100	medium high	0.100	medium low	0.516	medium high
20018	Cavriana	-0.628	low	-0.282	medium low	0.370	medium high	0.486	medium high
20019	Ceresara	-0.133	low	0.013	medium high	0.090	medium low	0.485	medium high
20020	Commessaggio	0.480	medium low	0.660	high	0.272	medium high	0.659	high
20029	Magnacavallo	1.051	medium high	0.319	high	0.661	high	0.669	high
20032	Mariana Mantovana	0.048	medium low	0.128	high	0.078	low	0.541	medium high
17186	Torbole Casaglia	-0.271	low	0.131	high	0.053	low	0.534	medium high
20056	San Giacomo delle Segnate	1.490	high	0.967	high	0.474	medium high	0.633	high
18002	Albaredo Arnaboldi	1.904	high	-0.636	low	0.676	high	0.538	medium high
18014	Bereguardo	2.100	high	-0.603	low	0.714	high	0.514	medium high
18015	Borgarello	2.113	high	-0.655	low	0.819	high	0.431	medium high
18018	Borgo San Siro	2.365	high	-0.381	medium low	0.515	high	0.409	medium low
18076	Gropello Cairoli	2.341	high	-0.396	medium low	0.606	high	0.445	medium high
35021	Fabbrico	1.772	high	1.339	high	0.691	high	0.643	high

Munic code	Comune	log_Ae. caspius	quartile class	log_Ae. vexans	quartile class	prob_Ae. caspius	quartile class	prob_Ae. vexans	quartile class
35022	Gattatico	0.283	medium low	0.782	high	0.060	low	0.432	medium high
35023	Gualtieri	1.077	medium high	1.508	high	0.332	medium high	0.693	high
35012	Casalgrande	0.100	medium low	0.490	high	0.146	medium low	0.275	medium low
35002	Bagnolo in Piano	1.204	medium high	0.847	high	0.195	medium low	0.545	medium high
18023	Bressana Bottarone	2.240	high	-0.472	low	0.675	high	0.506	medium high
17107	Mazzano	-0.822	low	-0.173	medium low	0.034	low	0.331	medium low
17132	Paitone	-0.790	low	-0.138	medium high	0.025	low	0.020	low
17108	Milzano	-0.189	low	0.389	high	0.037	low	0.650	high
18005	Arena Po	1.521	high	-0.235	medium low	0.650	high	0.530	medium high
18006	Badia Pavese	1.718	high	-0.001	medium high	0.773	high	0.707	high
17155	Prevalle	-0.831	low	-0.134	medium high	0.023	low	0.221	low
17192	Urago d'Oglio	0.018	low	-0.165	medium high	0.088	medium low	0.488	medium high
17122	Offlaga	-0.348	low	-0.204	medium low	0.036	low	0.579	high
34041	Torrile	0.246	medium low	1.236	high	0.052	low	0.515	medium high
35024	Guastalla	1.240	medium high	1.485	high	0.511	high	0.693	high
17188	Travagliato	-0.160	low	0.208	high	0.083	medium low	0.533	medium high
17200	Villachiara	0.111	medium low	0.162	high	0.092	medium low	0.601	high
34042	Traversetolo	-0.356	low	0.212	high	0.042	low	0.213	low
20054	Sabbioneta	0.515	medium low	0.989	high	0.210	medium low	0.678	high
18073	Godiasco Salice Terme	2.162	high	-0.032	medium high	0.125	medium low	0.116	low
18001	Alagna	2.606	high	-0.262	medium low	0.628	high	0.385	medium low
34045	Varano de' Melegari	-0.787	low	0.874	high	0.008	low	0.127	low
34035	Solignano	-0.782	low	0.767	high	0.005	low	0.093	low
17172	San Gervasio Bresciano	-0.253	low	0.234	high	0.028	low	0.598	high
18040	Castelnovetto	2.020	high	-0.506	low	0.272	medium high	0.376	medium low
18041	Cava Manara	2.397	high	-0.463	low	0.778	high	0.575	high
18024	Broni	1.482	high	-0.744	low	0.448	medium high	0.396	medium low
34029	Polesine Parmense	0.072	medium low	1.164	high	0.070	low	0.718	high
17161	Rezzato	-0.719	low	-0.117	medium high	0.055	low	0.366	medium low
17162	Roccafranca	-0.068	low	-0.017	medium high	0.141	medium low	0.625	high
18043	Ceranova	2.027	high	-0.686	low	0.522	high	0.485	medium high
35017	Cavriago	0.275	medium low	0.167	high	0.059	low	0.310	medium low
18026	Campospinoso	1.653	high	-0.702	low	0.658	high	0.495	medium high
18027	Candia Lomellina	2.130	high	-0.315	medium low	0.632	high	0.512	medium high
17173	San Zeno Naviglio	-0.522	low	-0.314	medium low	0.055	low	0.609	high
17112	Monticelli Brusati	0.079	medium low	0.736	high	0.011	low	0.117	low

Munic code	Comune	log_Ae. caspius	quartile class	log_Ae. vexans	quartile class	prob_Ae. caspius	quartile class	prob_Ae. vexans	quartile class
17126	Orzivecchi	-0.116	low	0.061	medium high	0.111	medium low	0.667	high
17127	Ospitaletto	-0.091	low	0.311	high	0.027	low	0.383	medium low
35018	Canossa	-0.226	low	0.168	high	0.022	low	0.085	low
34030	Roccabianca	0.210	medium low	1.490	high	0.044	low	0.679	high
18053	Copiano	1.969	high	-0.494	low	0.605	high	0.502	medium high
18044	Ceretto Lomellina	2.022	high	-0.481	low	0.429	medium high	0.403	medium low
34031	Sala Baganza	-0.751	low	0.882	high	0.015	low	0.194	low
18045	Cergnago	2.346	high	-0.268	medium low	0.449	medium high	0.321	medium low
18046	Certosa di Pavia	2.068	high	-0.678	low	0.618	high	0.396	medium low
17149	Pontevico	-0.174	low	0.390	high	0.033	low	0.589	high
17150	Pontoglio	-0.026	low	-0.196	medium low	0.110	medium low	0.511	medium high
17147	Poncarale	-0.493	low	-0.426	medium low	0.040	low	0.580	high
35020	Correggio	1.016	medium high	0.401	high	0.306	medium high	0.544	medium high
17113	Montichiari	-0.850	low	-0.318	medium low	0.062	low	0.502	medium high
17163	Rodengo Saiano	-0.072	low	0.505	high	0.037	low	0.308	medium low
18062	Ferrera Erbognone	2.427	high	-0.246	medium low	0.633	high	0.438	medium high
18063	Filighera	1.946	high	-0.458	low	0.510	high	0.507	medium high
17114	Montirone	-0.623	low	-0.470	low	0.045	low	0.575	high
17195	Verolanuova	-0.217	low	0.224	high	0.022	low	0.481	medium high
17165	Roncadelle	-0.276	low	0.177	high	0.028	low	0.476	medium high
35008	Cadelbosco di Sopra	1.046	medium high	0.971	high	0.241	medium low	0.609	high
35009	Campagnola Emilia	1.534	high	1.123	high	0.469	medium high	0.576	high
17196	Verolavecchia	-0.140	low	0.344	high	0.031	low	0.522	medium high
18030	Carbonara al Ticino	2.236	high	-0.519	low	0.608	high	0.510	medium high
18031	Casanova Lonati	1.884	high	-0.676	low	0.459	medium high	0.451	medium high
18032	Casatisma	2.144	high	-0.434	medium low	0.473	medium high	0.461	medium high
18054	Corana	2.470	high	-0.267	medium low	0.673	high	0.432	medium high
18056	Corteolona	1.841	high	-0.359	medium low	0.705	high	0.602	high
18057	Corvino San Quirico	1.966	high	-0.411	medium low	0.183	medium low	0.237	low
18047	Cervesina	2.422	high	-0.284	medium low	0.670	high	0.456	medium high
35010	Campegine	0.568	medium high	0.706	high	0.078	low	0.487	medium high
34032	Salsomaggiore Terme	-0.698	low	0.687	high	0.034	low	0.237	low
18048	Chignolo Po	1.694	high	0.070	medium high	0.669	high	0.632	high
18049	Cigognola	1.465	medium high	-0.440	medium low	0.364	medium high	0.226	low
17152	Pralboino	-0.136	low	0.483	high	0.017	low	0.516	medium high
17136	Passirano	0.027	medium low	0.593	high	0.031	low	0.294	medium low

Munic code	Comune	log_Ae. caspius	quartile class	log_Ae. vexans	quartile class	prob_Ae. caspius	quartile class	prob_Ae. vexans	quartile class
17137	Pavone del Mella	-0.306	low	0.152	high	0.038	low	0.629	high
18065	Frascarolo	1.972	high	-0.001	medium high	0.625	high	0.599	high
17116	Muscoline	-0.818	low	-0.116	medium high	0.040	low	0.217	low
17166	Rovato	-0.012	low	0.196	high	0.077	low	0.475	medium high
17167	Rudiano	-0.005	low	-0.127	medium high	0.139	medium low	0.570	medium high
18058	Costa de' Nobili	1.741	high	-0.253	medium low	0.590	high	0.602	high
18059	Cozzo	2.044	high	-0.490	low	0.431	medium high	0.469	medium high
18050	Cilavegna	2.061	high	-0.097	medium high	0.620	high	0.410	medium low
34033	San Secondo Parmense	0.173	medium low	1.086	high	0.021	low	0.496	medium high
18033	Casei Gerola	2.456	high	-0.176	medium low	0.530	high	0.389	medium low
18034	Casorate Primo	1.827	high	-0.801	low	0.678	high	0.363	medium low
18051	Codevilla	2.234	high	-0.072	medium high	0.138	medium low	0.172	low
18052	Confienza	2.057	high	-0.240	medium low	0.385	medium high	0.421	medium high
18035	Cassolnovo	1.876	high	0.056	medium high	0.298	medium high	0.389	medium low
17159	Quinzano d'Oglio	-0.031	low	0.352	high	0.069	low	0.554	medium high
17160	Remedello	-0.349	low	0.216	high	0.072	low	0.636	high
17119	Nuvolento	-0.824	low	-0.138	medium high	0.027	low	0.230	low
17120	Nuvolera	-0.837	low	-0.098	medium high	0.035	low	0.168	low
18060	Cura Carpignano	2.135	high	-0.584	low	0.675	high	0.497	medium high
18061	Dorno	2.483	high	-0.308	medium low	0.619	high	0.450	medium high
35015	Castelnovo di Sotto	0.958	medium high	1.198	high	0.159	medium low	0.541	medium high
35028	Novellara	1.394	medium high	1.224	high	0.379	medium high	0.597	high
26024	Crespano del Grappa	0.158	medium low	-0.169	medium high	0.061	low	0.076	low
26025	Crocetta del Montello	0.435	medium low	-0.451	medium low	0.101	medium low	0.326	medium low
26048	Moriago della Battaglia	0.411	medium low	-0.507	low	0.021	low	0.300	medium low
26045	Monfumo	0.331	medium low	-0.070	medium high	0.080	medium low	0.250	low
26049	Motta di Livenza	0.936	medium high	0.242	high	0.171	medium low	0.526	medium high
26050	Nervesa della Battaglia	0.386	medium low	-0.254	medium low	0.027	low	0.326	medium low
26046	Montebelluna	0.441	medium low	-0.449	medium low	0.195	medium low	0.429	medium high
26047	Morgano	0.434	medium low	-0.377	medium low	0.501	medium high	0.779	high
24101	Solagna	-0.288	low	-0.614	low	0.046	low	0.011	low
26064	Quinto di Treviso	0.455	medium low	-0.360	medium low	0.346	medium high	0.727	high
26065	Refrontolo	0.395	medium low	-0.212	medium low	0.025	low	0.248	low
24102	Sossano	0.027	medium low	-0.839	low	0.313	medium high	0.516	medium high
24103	Sovizzo	0.085	medium low	-0.526	low	0.129	medium low	0.332	medium low

Munic code	Comune	log_Ae. caspius	quartile class	log_Ae. vexans	quartile class	prob_Ae. caspius	quartile class	prob_Ae. vexans	quartile class
26041	Meduna di Livenza	0.685	medium high	0.006	medium high	0.154	medium low	0.508	medium high
24100	Schio	-0.709	low	-0.787	low	0.037	low	0.101	low
26021	Conegliano	0.317	medium low	0.003	medium high	0.044	low	0.393	medium low
26058	Ponte di Piave	0.683	medium high	-0.306	medium low	0.093	medium low	0.550	medium high
26044	Monastier di Treviso	1.055	medium high	-0.369	medium low	0.075	low	0.619	high
24091	Sandrigo	-0.187	low	-0.806	low	0.446	medium high	0.602	high
26008	Carbonera	0.474	medium low	-0.377	medium low	0.089	medium low	0.670	high
26059	Ponzano Veneto	0.321	medium low	-0.523	low	0.133	medium low	0.610	high
26060	Portobuffolè	0.411	medium low	-0.223	medium low	0.160	medium low	0.647	high
28005	Arquù Petrarca	0.379	medium low	-0.611	low	0.280	medium high	0.395	medium low
28006	Arre	1.159	medium high	-0.427	medium low	0.050	low	0.517	medium high
24092	San Germano dei Berici	0.122	medium low	-0.687	low	0.409	medium high	0.341	medium low
26082	Spresiano	0.380	medium low	-0.266	medium low	0.077	low	0.552	medium high
26073	San Pietro di Feletto	0.328	medium low	-0.010	medium high	0.023	low	0.235	low
26009	Casale sul Sile	1.040	medium high	-0.230	medium low	0.144	medium low	0.714	high
26056	Pederobba	0.392	medium low	-0.282	medium low	0.091	medium low	0.274	medium low
27011	Concordia Sagittaria	1.294	medium high	0.187	high	0.303	medium high	0.564	medium high
27012	Dolo	1.046	medium high	0.016	medium high	0.396	medium high	0.670	high
27014	Fiesso d'Artico	0.681	medium high	-0.275	medium low	0.600	high	0.654	high
26057	Pieve di Soligo	0.430	medium low	-0.268	medium low	0.024	low	0.253	low
24104	Tezze sul Brenta	-0.033	low	-0.394	medium low	0.374	medium high	0.505	medium high
26034	Gorgo al Monticano	0.756	medium high	0.003	medium high	0.138	medium low	0.565	medium high
26035	Istrana	0.389	medium low	-0.481	low	0.332	medium high	0.710	high
24105	Thiene	-0.691	low	-0.868	low	0.082	medium low	0.323	medium low
24086	Romano d'Ezzelino	-0.029	low	-0.304	medium low	0.133	medium low	0.273	medium low
26074	San Polo di Piave	0.435	medium low	-0.168	medium high	0.126	medium low	0.589	high
26075	Santa Lucia di Piave	0.380	medium low	-0.062	medium high	0.046	low	0.515	medium high
26010	Casier	0.737	medium high	-0.275	medium low	0.182	medium low	0.675	high
26036	Loria	0.340	medium low	0.153	high	0.256	medium high	0.473	medium high
26043	Mogliano Veneto	1.044	medium high	-0.083	medium high	0.182	medium low	0.659	high
26076	San Vendemiano	0.311	medium low	0.039	medium high	0.064	low	0.507	medium high
27013	Eraclea	1.643	high	-0.146	medium high	0.196	medium low	0.442	medium high
26011	Castelcucco	0.283	medium low	-0.129	medium high	0.136	medium low	0.238	low
26023	Cornuda	0.427	medium low	-0.329	medium low	0.123	medium low	0.272	medium low

Munic code	Comune	log_Ae. caspius	quartile class	log_Ae. vexans	quartile class	prob_Ae. caspius	quartile class	prob_Ae. vexans	quartile class
26066	Resana	0.207	medium low	-0.103	medium high	0.514	high	0.664	high
26037	Mansuè	0.512	medium low	-0.155	medium high	0.115	medium low	0.606	high
24095	Santorso	-0.769	low	-0.748	low	0.090	medium low	0.123	low
24096	San Vito di Leguzzano	-0.652	low	-0.724	low	0.139	medium low	0.243	low
27015	Fossalta di Piave	1.284	medium high	-0.261	medium low	0.172	medium low	0.641	high
26068	Riese Pio X	0.574	medium high	0.256	high	0.299	medium high	0.534	medium high
24107	Torrebelvicino	-0.654	low	-0.297	medium low	0.011	low	0.031	low
24087	Rosù	-0.054	low	-0.358	medium low	0.177	medium low	0.418	medium low
26054	Paderno del Grappa	0.234	medium low	-0.115	medium high	0.044	low	0.048	low
24108	Torri di Quartesolo	0.512	medium low	-0.236	medium low	0.691	high	0.612	high
24110	Trissino	-0.088	low	-0.690	low	0.121	medium low	0.199	low
26038	Mareno di Piave	0.366	medium low	-0.026	medium high	0.058	low	0.527	medium high
26055	Paese	0.367	medium low	-0.532	low	0.246	medium low	0.654	high
24097	Sarcedo	-0.641	low	-0.903	low	0.134	medium low	0.431	medium high
26012	Castelfranco Veneto	0.324	medium low	0.043	medium high	0.417	medium high	0.591	high
26039	Maser	0.466	medium low	-0.106	medium high	0.069	low	0.212	low
26040	Maserada sul Piave	0.440	medium low	-0.288	medium low	0.059	low	0.567	medium high
24088	Rossano Veneto	0.163	medium low	-0.034	medium high	0.215	medium low	0.414	medium low
24098	Sarego	0.158	medium low	-0.406	medium low	0.238	medium low	0.380	medium low
24099	Schiavon	-0.265	low	-0.831	low	0.215	medium low	0.519	medium high
27019	Jesolo	1.682	high	-0.619	low	0.308	medium high	0.490	medium high
27005	Caorle	1.620	high	-0.192	medium low	0.577	high	0.607	high
27008	Chioggia	1.747	high	0.131	high	0.333	medium high	0.655	high
27010	Cona	1.544	high	-0.110	medium high	0.229	medium low	0.708	high
26005	Breda di Piave	0.540	medium low	-0.350	medium low	0.100	medium low	0.609	high
26006	Caerano di San Marco	0.475	medium low	-0.223	medium low	0.162	medium low	0.407	medium low
27024	Mirano	0.869	medium high	0.086	medium high	0.287	medium high	0.614	high
27025	Musile di Piave	1.577	high	-0.324	medium low	0.287	medium high	0.726	high
26089	Vedelago	0.414	medium low	-0.262	medium low	0.335	medium high	0.638	high
26090	Vidor	0.424	medium low	-0.506	low	0.064	low	0.292	medium low
27002	Campagna Lupia	1.524	high	0.164	high	0.131	medium low	0.592	high
27003	Campolongo Maggiore	0.950	medium high	-0.354	medium low	0.210	medium low	0.632	high
27004	Camponogara	1.121	medium high	-0.069	medium high	0.177	medium low	0.585	high
26081	Silea	0.856	medium high	-0.324	medium low	0.148	medium low	0.727	high

Munic code	Comune	log_Ae. caspius	quartile class	log_Ae. vexans	quartile class	prob_Ae. caspius	quartile class	prob_Ae. vexans	quartile class
27026	Noale	0.521	medium low	-0.152	medium high	0.394	medium high	0.648	high
26007	Cappella Maggiore	0.177	medium low	-0.310	medium low	0.023	low	0.248	low
27020	Marcon	1.421	medium high	-0.137	medium high	0.129	medium low	0.680	high
27027	Noventa di Piave	1.240	medium high	-0.056	medium high	0.291	medium high	0.701	high
26071	San Biagio di Callalta	0.736	medium high	-0.376	medium low	0.087	medium low	0.630	high
27028	Pianiga	0.597	medium high	-0.207	medium low	0.389	medium high	0.588	high
27021	Martellago	0.909	medium high	-0.006	medium high	0.352	medium high	0.699	high
26091	Villorba	0.329	medium low	-0.414	medium low	0.120	medium low	0.629	high
23049	Montecchia di Crosara	0.047	medium low	-0.238	medium low	0.107	medium low	0.174	low
23050	Monteforte d'Alpone	0.102	medium low	-0.188	medium low	0.094	medium low	0.291	medium low
26083	Susegana	0.396	medium low	-0.125	medium high	0.015	low	0.292	medium low
27029	Portogruaro	1.140	medium high	0.162	high	0.395	medium high	0.580	high
26093	Volpago del Montello	0.386	medium low	-0.662	low	0.058	low	0.282	medium low
26094	Zenson di Piave	1.020	medium high	-0.328	medium low	0.254	medium high	0.744	high
26085	Trevignano	0.391	medium low	-0.699	low	0.218	medium low	0.570	medium high
26013	Castello di Godego	0.377	medium low	0.187	high	0.445	medium high	0.615	high
26086	Treviso	0.463	medium low	-0.383	medium low	0.196	medium low	0.647	high
23012	Bovolone	0.901	medium high	-0.408	medium low	0.866	high	0.643	high
26014	Cavaso del Tomba	0.289	medium low	-0.164	medium high	0.058	low	0.106	low
26015	Cessalto	1.232	medium high	0.439	high	0.129	medium low	0.503	medium high
27022	Meolo	1.437	medium high	-0.337	medium low	0.161	medium low	0.710	high
26092	Vittorio Veneto	0.191	medium low	-0.365	medium low	0.010	low	0.115	low
26016	Chiarano	0.999	medium high	0.133	high	0.088	medium low	0.524	medium high
27030	Pramaggiore	0.769	medium high	0.160	high	0.159	medium low	0.479	medium high
27016	Fossalta di Portogruaro	1.037	medium high	0.155	high	0.358	medium high	0.510	medium high
26017	Cimadolmo	0.430	medium low	-0.199	medium low	0.043	low	0.501	medium high
27031	Quarto d'Altino	1.687	high	-0.296	medium low	0.189	medium low	0.754	high
27017	Fossù	0.819	medium high	-0.346	medium low	0.425	medium high	0.665	high
27018	Gruaro	0.692	medium high	0.132	high	0.329	medium high	0.565	medium high
27006	Cavarzere	1.835	high	0.093	medium high	0.324	medium high	0.728	high
27007	Ceggia	1.442	medium high	0.611	high	0.359	medium high	0.634	high
27009	Cinto Caomaggiore	0.662	medium high	0.102	medium high	0.290	medium high	0.596	high
26001	Altivole	0.550	medium low	0.040	medium high	0.099	medium low	0.401	medium low
26002	Arcade	0.356	medium low	-0.329	medium low	0.052	low	0.491	medium high

Munic code	Comune	log_Ae. caspius	quartile class	log_Ae. vexans	quartile class	prob_Ae. caspius	quartile class	prob_Ae. vexans	quartile class
26003	Asolo	0.455	medium low	0.070	medium high	0.188	medium low	0.377	medium low
27023	Mira	1.474	high	0.192	high	0.323	medium high	0.697	high
26087	Valdobbiadene	0.393	medium low	-0.359	medium low	0.025	low	0.063	low
26088	Vazzola	0.381	medium low	-0.068	medium high	0.039	low	0.473	medium high
26095	Zero Branco	0.562	medium high	-0.254	medium low	0.322	medium high	0.634	high
27001	Annone Veneto	0.991	medium high	0.330	high	0.339	medium high	0.614	high
26004	Borso del Grappa	0.024	low	-0.244	medium low	0.073	low	0.061	low
26077	San Zenone degli Ezzelini	0.272	medium low	0.035	medium high	0.229	medium low	0.408	medium low
26078	Sarmede	0.185	medium low	-0.240	medium low	0.011	low	0.051	low
26019	Codognè	0.316	medium low	-0.050	medium high	0.085	medium low	0.518	medium high
26020	Colle Umberto	0.238	medium low	-0.126	medium high	0.039	low	0.446	medium high
26072	San Fior	0.282	medium low	-0.016	medium high	0.044	low	0.448	medium high
27032	Salzano	0.753	medium high	-0.008	medium high	0.253	medium high	0.626	high
27033	San Donù di Piave	1.526	high	-0.022	medium high	0.267	medium high	0.575	high
26080	Sernaglia della Battaglia	0.438	medium low	-0.398	medium low	0.058	low	0.384	medium low
23037	Gazzo Veronese	1.299	medium high	0.172	high	0.701	high	0.685	high
23024	Cazzano di Tramigna	-0.008	low	-0.217	medium low	0.248	medium low	0.089	low
23047	Mezzane di Sotto	-0.218	low	-0.424	medium low	0.280	medium high	0.112	low
23048	Minerbe	-0.204	low	-0.807	low	0.693	high	0.694	high
23025	Cerea	0.919	medium high	-0.176	medium low	0.786	high	0.639	high
24038	Dueville	-0.171	low	-0.723	low	0.427	medium high	0.531	medium high
23038	Grezzana	-0.411	low	-0.487	low	0.166	medium low	0.075	low
23039	Illasi	-0.137	low	-0.367	medium low	0.227	medium low	0.184	low
23058	Pescantina	-0.660	low	-0.402	medium low	0.332	medium high	0.485	medium high
23060	Povegliano Veronese	0.251	medium low	-0.783	low	0.839	high	0.700	high
23094	Vigasio	0.427	medium low	-0.761	low	0.800	high	0.680	high
23095	Villa Bartolomea	0.838	medium high	-0.472	low	0.514	high	0.665	high
23019	Casaleone	1.105	medium high	-0.008	medium high	0.712	high	0.621	high
24075	Orgiano	-0.049	low	-0.793	low	0.405	medium high	0.501	medium high
24077	Pianezze	-0.561	low	-1.013	low	0.083	medium low	0.303	medium low
23020	Castagnaro	1.023	medium high	-0.367	medium low	0.572	high	0.668	high
23051	Mozzecane	0.549	medium low	-0.818	low	0.874	high	0.707	high
23021	Castel d'Azzano	-0.009	low	-0.693	low	0.715	high	0.696	high
23040	Isola della Scala	0.928	medium high	-0.562	low	0.775	high	0.623	high
23041	Isola Rizza	0.624	medium high	-0.294	medium low	0.621	high	0.590	high

Munic code	Comune	log_Ae. caspius	quartile class	log_Ae. vexans	quartile class	prob_Ae. caspius	quartile class	prob_Ae. vexans	quartile class
23032	Erbè	1.348	medium high	-0.503	low	0.681	high	0.632	high
23042	Lavagno	-0.101	low	-0.301	medium low	0.552	high	0.377	medium low
23052	Negrar	-0.553	low	-0.365	medium low	0.158	medium low	0.091	low
23053	Nogara	1.362	medium high	-0.061	medium high	0.614	high	0.585	high
23044	Legnago	0.604	medium high	-0.468	low	0.525	high	0.632	high
23054	Nogarole Rocca	0.951	medium high	-0.728	low	0.803	high	0.687	high
23015	Bussolengo	-0.659	low	-0.480	low	0.284	medium high	0.388	medium low
23016	Buttapietra	0.172	medium low	-0.637	low	0.800	high	0.730	high
23017	Caldiero	0.065	medium low	-0.216	medium low	0.439	medium high	0.513	medium high
23064	Ronco all'Adige	0.358	medium low	-0.118	medium high	0.639	high	0.804	high
24069	Mossano	0.368	medium low	-0.583	low	0.304	medium high	0.360	medium low
24070	Mussolente	0.138	medium low	-0.102	medium high	0.260	medium high	0.462	medium high
24035	Costabissara	-0.159	low	-0.552	low	0.334	medium high	0.432	medium high
23002	Albaredo d'Adige	0.116	medium low	-0.375	medium low	0.456	medium high	0.651	high
23003	Angiari	0.418	medium low	-0.460	low	0.727	high	0.756	high
23004	Arcole	0.140	medium low	-0.139	medium high	0.181	medium low	0.510	medium high
24036	Creazzo	0.071	medium low	-0.510	low	0.340	medium high	0.348	medium low
23072	Sanguinetto	1.161	medium high	-0.023	medium high	0.435	medium high	0.464	medium high
23073	San Martino Buon Albergo	-0.075	low	-0.381	medium low	0.640	high	0.570	medium high
23081	Soave	0.072	medium low	-0.160	medium high	0.222	medium low	0.225	low
23082	Sommacampagna	-0.376	low	-0.633	low	0.306	medium high	0.435	medium high
23083	Sona	-0.577	low	-0.532	low	0.312	medium high	0.379	medium low
23084	Sorgù	1.493	high	-0.154	medium high	0.768	high	0.657	high
23055	Oppeano	0.580	medium high	-0.443	medium low	0.695	high	0.630	high
23056	Palù	0.491	medium low	-0.311	medium low	0.719	high	0.755	high
24071	Nanto	0.380	medium low	-0.508	low	0.416	medium high	0.391	medium low
24014	Breganze	-0.494	low	-0.945	low	0.215	medium low	0.414	medium low
24057	Marostica	-0.500	low	-0.938	low	0.122	medium low	0.193	low
24058	Mason Vicentino	-0.495	low	-0.989	low	0.115	medium low	0.439	medium high
24059	Molvena	-0.613	low	-1.034	low	0.049	low	0.203	low
24060	Montebello Vicentino	0.183	medium low	-0.405	medium low	0.085	medium low	0.392	medium low
24061	Montecchio Maggiore	0.158	medium low	-0.485	low	0.137	medium low	0.360	medium low
24062	Montecchio Precalcino	-0.442	low	-0.840	low	0.310	medium high	0.490	medium high
23091	Verona	-0.365	low	-0.523	low	0.384	medium high	0.425	medium high

Munic code	Comune	log_Ae. caspius	quartile class	log_Ae. vexans	quartile class	prob_Ae. caspius	quartile class	prob_Ae. vexans	quartile class
24012	Bassano del Grappa	-0.251	low	-0.617	low	0.118	medium low	0.260	low
24025	Cartigliano	-0.226	low	-0.674	low	0.370	medium high	0.525	medium high
24026	Cassola	0.041	medium low	-0.210	medium low	0.195	medium low	0.414	medium low
24027	Castegnero	0.392	medium low	-0.417	medium low	0.457	medium high	0.350	medium low
24028	Castelgomberto	-0.200	low	-0.720	low	0.203	medium low	0.319	medium low
24029	Chiampo	-0.051	low	-0.208	medium low	0.057	low	0.066	low
24030	Chiuppano	-0.807	low	-0.744	low	0.043	low	0.162	low
24073	Nove	-0.356	low	-0.844	low	0.357	medium high	0.515	medium high
24074	Noventa Vicentina	-0.047	low	-0.934	low	0.418	medium high	0.611	high
23075	San Pietro di Morubio	0.695	medium high	-0.313	medium low	0.694	high	0.569	medium high
20065	Suzzara	1.016	medium high	1.331	high	0.496	medium high	0.694	high
23007	Belfiore	0.213	medium low	-0.075	medium high	0.495	medium high	0.733	high
23065	Roverchiara	0.412	medium low	-0.358	medium low	0.643	high	0.726	high
23066	Roveredo di Guù	-0.196	low	-0.851	low	0.557	high	0.663	high
24040	Fara Vicentino	-0.660	low	-0.946	low	0.160	medium low	0.250	low
23076	San Pietro in Cariano	-0.596	low	-0.373	medium low	0.279	medium high	0.339	medium low
24051	Longare	0.422	medium low	-0.269	medium low	0.418	medium high	0.376	medium low
23085	Terrazzo	0.421	medium low	-0.630	low	0.681	high	0.776	high
23087	Tregnago	-0.238	low	-0.518	low	0.199	medium low	0.067	low
24052	Lonigo	0.060	medium low	-0.426	medium low	0.322	medium high	0.566	medium high
24015	Brendola	0.222	medium low	-0.444	medium low	0.343	medium high	0.354	medium low
24016	Bressanvido	0.004	low	-0.680	low	0.231	medium low	0.477	medium high
24063	Monte di Malo	-0.514	low	-0.524	low	0.054	low	0.070	low
24064	Montegalda	0.426	medium low	-0.207	medium low	0.303	medium high	0.475	medium high
24065	Montegaldella	0.403	medium low	-0.311	medium low	0.311	medium high	0.492	medium high
24008	Arzignano	0.056	medium low	-0.533	low	0.052	low	0.177	low
24017	Brogliano	-0.243	low	-0.640	low	0.078	low	0.100	low
20066	Viadana	0.715	medium high	1.296	high	0.374	medium high	0.745	high
23008	Bevilacqua	-0.053	low	-0.781	low	0.762	high	0.742	high
23009	Bonavigo	0.107	medium low	-0.593	low	0.613	high	0.793	high
23010	Boschi Sant'Anna	0.037	medium low	-0.754	low	0.559	high	0.639	high
23096	Villafranca di Verona	-0.016	low	-0.755	low	0.537	high	0.593	high
23088	Trevenzuolo	1.125	medium high	-0.642	low	0.535	high	0.591	high
23068	Salizzole	1.182	medium high	-0.270	medium low	0.592	high	0.539	medium high
24018	Caldogno	-0.212	low	-0.636	low	0.410	medium high	0.548	medium high
23097	Zevio	0.230	medium low	-0.314	medium low	0.698	high	0.730	high

Munic code	Comune	log_Ae. caspius	quartile class	log_Ae. vexans	quartile class	prob_Ae. caspius	quartile class	prob_Ae. vexans	quartile class
23098	Zimella	-0.004	low	-0.426	medium low	0.312	medium high	0.604	high
24001	Agugliaro	0.068	medium low	-0.860	low	0.390	medium high	0.648	high
23089	Valeggio sul Mincio	-0.025	low	-0.734	low	0.554	high	0.540	medium high
24066	Monteviale	-0.037	low	-0.527	low	0.308	medium high	0.353	medium low
24043	Gambellara	0.128	medium low	-0.274	medium low	0.027	low	0.256	low
24045	Grancona	0.152	medium low	-0.210	medium low	0.325	medium high	0.292	medium low
24019	Caltrano	-0.800	low	-0.735	low	0.042	low	0.010	low
24002	Albettone	0.226	medium low	-0.725	low	0.362	medium high	0.610	high
24003	Alonte	-0.023	low	-0.650	low	0.500	medium high	0.399	medium low
24067	Monticello Conte Otto	0.081	medium low	-0.574	low	0.464	medium high	0.513	medium high
23061	Pressana	-0.223	low	-0.776	low	0.506	high	0.683	high
23063	Roncù	0.049	medium low	-0.247	medium low	0.038	low	0.090	low
24034	Cornedo Vicentino	-0.318	low	-0.725	low	0.081	medium low	0.125	low
23069	San Bonifacio	0.136	medium low	-0.146	medium high	0.288	medium high	0.583	high
23071	San Giovanni Lupatoto	-0.020	low	-0.522	low	0.607	high	0.635	high
24004	Altavilla Vicentina	0.145	medium low	-0.515	low	0.313	medium high	0.372	medium low
23027	Cologna Veneta	-0.142	low	-0.680	low	0.458	medium high	0.634	high
23028	Colognola ai Colli	-0.004	low	-0.216	medium low	0.481	medium high	0.363	medium low
23029	Concamarise	1.095	medium high	-0.184	medium low	0.707	high	0.498	medium high
24046	Grisignano di Zocco	0.430	medium low	-0.127	medium high	0.612	high	0.589	high
24047	Grumolo delle Abbadesse	0.526	medium low	-0.164	medium high	0.621	high	0.596	high
24048	Isola Vicentina	-0.363	low	-0.608	low	0.150	medium low	0.275	medium low
24055	Malo	-0.575	low	-0.662	low	0.137	medium low	0.356	medium low
24056	Marano Vicentino	-0.728	low	-0.787	low	0.057	low	0.332	medium low
24006	Arcugnano	0.206	medium low	-0.438	medium low	0.364	medium high	0.265	low
24078	Piovene Rocchette	-0.798	low	-0.716	low	0.034	low	0.066	low
24079	Pojana Maggiore	-0.133	low	-0.915	low	0.440	medium high	0.627	high
23092	Veronella	0.026	medium low	-0.365	medium low	0.327	medium high	0.577	high
24068	Montorso Vicentino	0.161	medium low	-0.378	medium low	0.090	medium low	0.265	low
24010	Asigliano Veneto	-0.147	low	-0.825	low	0.445	medium high	0.634	high
24011	Barbarano Vicentino	0.328	medium low	-0.658	low	0.340	medium high	0.397	medium low
24013	Bolzano Vicentino	0.240	medium low	-0.520	low	0.433	medium high	0.538	medium high
24021	Camisano Vicentino	0.512	medium low	-0.186	medium low	0.540	high	0.597	high
24022	Campiglia dei Berici	0.024	low	-0.881	low	0.453	medium high	0.680	high
24024	Carrè	-0.812	low	-0.791	low	0.036	low	0.176	low
29029	Lendinara	1.043	medium high	-0.614	low	0.408	medium high	0.667	high

Munic code	Comune	log_Ae. caspius	quartile class	log_Ae. vexans	quartile class	prob_Ae. caspius	quartile class	prob_Ae. vexans	quartile class
29031	Lusia	0.929	medium high	-0.693	low	0.373	medium high	0.638	high
28064	Piombino Dese	0.289	medium low	-0.231	medium low	0.531	high	0.696	high
28065	Piove di Sacco	1.014	medium high	-0.417	medium low	0.295	medium high	0.680	high
27036	San Stino di Livenza	1.368	medium high	0.338	high	0.345	medium high	0.607	high
28038	Fontaniva	0.182	medium low	-0.248	medium low	0.618	high	0.630	high
28039	Galliera Veneta	0.188	medium low	0.008	medium high	0.551	high	0.585	high
28040	Galzignano Terme	0.397	medium low	-0.605	low	0.396	medium high	0.394	medium low
28001	Abano Terme	0.294	medium low	-0.674	low	0.483	medium high	0.630	high
28002	Agna	1.390	medium high	-0.239	medium low	0.320	medium high	0.689	high
28003	Albignasego	0.324	medium low	-0.786	low	0.552	high	0.655	high
29002	Ariano nel Polesine	2.022	high	0.870	high	0.822	high	0.749	high
29005	Bagnolo di Po	1.447	medium high	-0.176	medium low	0.571	high	0.772	high
28032	Cittadella	0.104	medium low	-0.205	medium low	0.476	medium high	0.554	medium high
28033	Codevigo	1.445	medium high	-0.018	medium high	0.246	medium low	0.629	high
28085	Saonara	0.415	medium low	-0.702	low	0.567	high	0.647	high
28086	Selvazzano Dentro	0.238	medium low	-0.537	low	0.698	high	0.608	high
28055	Monselice	0.668	medium high	-0.616	low	0.378	medium high	0.631	high
28056	Montagnana	-0.128	low	-0.774	low	0.728	high	0.703	high
17029	Brescia	-0.455	low	-0.076	medium high	0.034	low	0.312	medium low
28075	San Giorgio delle Pertiche	0.054	medium low	-0.296	medium low	0.474	medium high	0.622	high
28076	San Giorgio in Bosco	0.188	medium low	-0.146	medium high	0.669	high	0.694	high
29041	Rovigo	1.649	high	-0.008	medium high	0.523	high	0.738	high
29042	Salara	1.367	medium high	0.122	high	0.602	high	0.787	high
28099	Vigodarzere	0.107	medium low	-0.507	low	0.549	high	0.619	high
28100	Vigonza	0.226	medium low	-0.555	low	0.561	high	0.666	high
28019	Camposampiero	0.032	medium low	-0.176	medium low	0.629	high	0.691	high
28020	Campo San Martino	0.121	medium low	-0.192	medium low	0.622	high	0.676	high
28021	Candiana	1.169	medium high	-0.426	medium low	0.105	medium low	0.571	medium high
28077	San Martino di Lupari	0.137	medium low	0.040	medium high	0.560	high	0.600	high
29040	Rosolina	2.272	high	0.243	high	0.588	high	0.587	high
28041	Gazzo	0.591	medium high	-0.291	medium low	0.434	medium high	0.563	medium high
28042	Grantorto	0.340	medium low	-0.300	medium low	0.588	high	0.652	high
28043	Granze	0.874	medium high	-0.635	low	0.225	medium low	0.626	high
29039	Porto Tolle	2.072	high	0.659	high	0.811	high	0.758	high
28004	Anguillara Veneta	1.436	medium high	-0.131	medium high	0.313	medium high	0.698	high
28066	Polverara	0.499	medium low	-0.848	low	0.234	medium low	0.581	high

Munic code	Comune	log_Ae. caspius	quartile class	log_Ae. vexans	quartile class	prob_Ae. caspius	quartile class	prob_Ae. vexans	quartile class
28067	Ponso	0.264	medium low	-0.582	low	0.611	high	0.659	high
28068	Pontelongo	1.149	medium high	-0.408	medium low	0.325	medium high	0.726	high
29006	Bergantino	1.058	medium high	-0.044	medium high	0.842	high	0.754	high
27037	Scorzè	0.670	medium high	-0.153	medium high	0.210	medium low	0.576	high
28087	Solesino	0.854	medium high	-0.630	low	0.296	medium high	0.609	high
28088	Stanghella	1.101	medium high	-0.522	low	0.282	medium high	0.680	high
29026	Gavello	2.047	high	0.927	high	0.664	high	0.747	high
30036	Faedis	-0.610	low	-0.095	medium high	0.016	low	0.098	low
29004	Badia Polesine	1.098	medium high	-0.440	medium low	0.684	high	0.740	high
28022	Carceri	0.320	medium low	-0.574	low	0.385	medium high	0.608	high
28034	Conselve	1.028	medium high	-0.477	low	0.024	low	0.440	medium high
28035	Correzzola	1.380	medium high	-0.170	medium high	0.383	medium high	0.733	high
29027	Giacciano con Baruchella	1.222	medium high	-0.298	medium low	0.684	high	0.728	high
29028	Guarda Veneta	2.230	high	0.859	high	0.433	medium high	0.731	high
29030	Loreo	2.173	high	0.313	high	0.714	high	0.795	high
30072	Pasian di Prato	-0.413	low	-0.129	medium high	0.081	medium low	0.418	medium low
17032	Calcinato	-0.914	low	-0.189	medium low	0.024	low	0.361	medium low
27035	Santa Maria di Sala	0.459	medium low	-0.180	medium low	0.236	medium low	0.524	medium high
28078	San Pietro in Gu	0.287	medium low	-0.482	low	0.356	medium high	0.537	medium high
28079	San Pietro Viminario	0.832	medium high	-0.534	low	0.112	medium low	0.523	medium high
28080	Santa Giustina in Colle	-0.047	low	-0.132	medium high	0.328	medium high	0.616	high
28044	Legnaro	0.394	medium low	-0.860	low	0.370	medium high	0.614	high
28045	Limena	0.170	medium low	-0.451	medium low	0.784	high	0.658	high
28046	Loreggia	0.031	medium low	-0.108	medium high	0.646	high	0.686	high
31014	Mossa	0.000	low	0.000	medium high	0.047	low	0.505	medium high
31015	Romans d'Isonzo	0.474	medium low	0.162	high	0.118	medium low	0.554	medium high
31016	Ronchi dei Legionari	1.167	medium high	0.178	high	0.078	low	0.448	medium high
28023	Carmignano di Brenta	0.169	medium low	-0.442	medium low	0.618	high	0.603	high
28026	Cartura	0.703	medium high	-0.613	low	0.059	low	0.480	medium high
28027	Casale di Scodosia	0.169	medium low	-0.516	low	0.820	high	0.713	high
29023	Frassinelle Polesine	1.600	high	0.088	medium high	0.305	medium high	0.775	high
29024	Fratta Polesine	1.281	medium high	-0.407	medium low	0.396	medium high	0.717	high
29025	Gaiba	1.471	high	0.079	medium high	0.641	high	0.778	high
28089	Teolo	0.279	medium low	-0.499	low	0.429	medium high	0.370	medium low
28090	Terrassa Padovana	0.925	medium high	-0.567	low	0.020	low	0.460	medium high

Munic code	Comune	log_Ae. caspius	quartile class	log_Ae. vexans	quartile class	prob_Ae. caspius	quartile class	prob_Ae. vexans	quartile class
28091	Tombolo	0.137	medium low	-0.027	medium high	0.722	high	0.621	high
30037	Fagagna	-0.476	low	-0.146	medium high	0.049	low	0.277	medium low
28069	Ponte San Nicolù	0.317	medium low	-0.846	low	0.460	medium high	0.616	high
28070	Pozzonovo	1.143	medium high	-0.439	medium low	0.189	medium low	0.647	high
28071	Rovolon	0.320	medium low	-0.520	low	0.393	medium high	0.419	medium low
27038	Spinea	1.119	medium high	0.151	high	0.276	medium high	0.620	high
27039	Stra	0.674	medium high	-0.331	medium low	0.477	medium high	0.677	high
29036	Pincara	1.414	medium high	-0.199	medium low	0.412	medium high	0.776	high
29037	Polesella	1.868	high	0.425	high	0.496	medium high	0.742	high
29038	Pontecchio Polesine	2.119	high	0.723	high	0.540	high	0.751	high
28047	Lozzo Atestino	0.077	medium low	-0.776	low	0.424	medium high	0.541	medium high
30038	Fiumicello	1.461	medium high	0.131	high	0.174	medium low	0.563	medium high
29007	Bosaro	2.006	high	0.534	high	0.619	high	0.765	high
29008	Calto	1.194	medium high	0.217	high	0.610	high	0.789	high
29009	Canaro	1.481	high	0.150	high	0.399	medium high	0.768	high
28036	Curtarolo	0.132	medium low	-0.299	medium low	0.594	high	0.684	high
28037	Este	0.302	medium low	-0.634	low	0.440	medium high	0.590	high
28081	Santa Margherita d'Adige	0.184	medium low	-0.599	low	0.635	high	0.659	high
28048	Maserù di Padova	0.467	medium low	-0.728	low	0.235	medium low	0.557	medium high
28049	Masi	0.783	medium high	-0.452	medium low	0.674	high	0.773	high
28050	Massanzago	0.302	medium low	-0.212	medium low	0.565	high	0.692	high
28051	Megliadino San Fidenzio	-0.012	low	-0.710	low	0.738	high	0.693	high
30074	Pavia di Udine	-0.177	low	-0.120	medium high	0.112	medium low	0.426	medium high
31017	Sagrado	0.601	medium high	0.121	high	0.040	low	0.253	low
29010	Canda	1.375	medium high	-0.381	medium low	0.616	high	0.749	high
28028	Casalserugo	0.523	medium low	-0.779	low	0.151	medium low	0.543	medium high
28029	Castelbaldo	0.697	medium high	-0.461	low	0.712	high	0.777	high
28082	Sant'Angelo di Piove di Sacco	0.573	medium high	-0.704	low	0.407	medium high	0.650	high
28083	Sant'Elena	0.621	medium high	-0.666	low	0.515	high	0.681	high
28084	Sant'Urbano	0.716	medium high	-0.704	low	0.515	high	0.734	high
28092	Torreglia	0.309	medium low	-0.580	low	0.361	medium high	0.422	medium high
28093	Trebaseleghe	0.368	medium low	-0.241	medium low	0.477	medium high	0.667	high
28094	Tribano	1.158	medium high	-0.441	medium low	0.096	medium low	0.542	medium high
31018	San Canzian d'Isonzo	1.520	high	0.144	high	0.073	low	0.638	high
29043	San Bellino	1.266	medium high	-0.455	medium low	0.284	medium high	0.638	high
27034	San Michele al Tagliamento	1.468	medium high	-0.056	medium high	0.557	high	0.583	high

Munic code	Comune	log_Ae. caspius	quartile class	log_Ae. vexans	quartile class	prob_Ae. caspius	quartile class	prob_Ae. vexans	quartile class
29032	Melara	1.078	medium high	0.095	medium high	0.823	high	0.731	high
29033	Occhiobello	1.315	medium high	-0.027	medium high	0.491	medium high	0.759	high
29034	Papozze	1.927	high	1.013	high	0.725	high	0.766	high
29035	Pettorazza Grimani	1.701	high	0.204	high	0.414	medium high	0.762	high
29013	Castelnovo Bariano	1.078	medium high	-0.126	medium high	0.725	high	0.799	high
29014	Ceneselli	1.195	medium high	-0.039	medium high	0.667	high	0.773	high
29015	Ceregnano	1.960	high	0.609	high	0.578	high	0.714	high
29017	Corbola	2.005	high	0.847	high	0.572	high	0.679	high
29044	San Martino di Venezze	1.586	high	0.095	medium high	0.488	medium high	0.753	high
29045	Stienta	1.444	medium high	-0.062	medium high	0.578	high	0.787	high
28063	Piazzola sul Brenta	0.311	medium low	-0.237	medium low	0.542	high	0.639	high
28030	Cervarese Santa Croce	0.346	medium low	-0.413	medium low	0.523	high	0.558	medium high
28031	Cinto Euganeo	0.148	medium low	-0.680	low	0.296	medium high	0.263	low
28072	Rubano	0.228	medium low	-0.447	medium low	0.788	high	0.631	high
27040	Teglio Veneto	0.793	medium high	0.167	high	0.265	medium high	0.488	medium high
27041	Torre di Mosto	1.524	high	0.448	high	0.297	medium high	0.523	medium high
27042	Venezia	1.662	high	-0.106	medium high	0.194	medium low	0.597	high
28012	Boara Pisani	1.342	medium high	-0.311	medium low	0.332	medium high	0.741	high
27043	Vigonovo	0.552	medium low	-0.522	low	0.513	high	0.698	high
27044	Cavallino-Treporti	1.831	high	-0.365	medium low	0.286	medium high	0.518	medium high
29001	Adria	1.923	high	0.592	high	0.542	high	0.716	high
28073	Saccolongo	0.295	medium low	-0.433	medium low	0.519	high	0.634	high
28074	Saletto	0.008	low	-0.761	low	0.626	high	0.668	high
28052	Megliadino San Vitale	0.263	medium low	-0.550	low	0.742	high	0.738	high
28053	Merlara	0.396	medium low	-0.498	low	0.682	high	0.764	high
28054	Mestrino	0.333	medium low	-0.252	medium low	0.573	high	0.613	high
29011	Castelguglielmo	1.391	medium high	-0.304	medium low	0.463	medium high	0.710	high
29012	Castelmassa	1.139	medium high	0.065	medium high	0.706	high	0.734	high
28095	Urbana	0.050	medium low	-0.676	low	0.685	high	0.691	high
28096	Veggiano	0.363	medium low	-0.267	medium low	0.591	high	0.626	high
28097	Vescovana	1.097	medium high	-0.576	low	0.259	medium high	0.686	high
28098	Vighizzolo d'Este	0.500	medium low	-0.606	low	0.500	medium high	0.732	high
28016	Cadoneghe	0.078	medium low	-0.639	low	0.657	high	0.661	high
28017	Campodarsego	0.099	medium low	-0.444	medium low	0.576	high	0.649	high
28018	Campodoro	0.367	medium low	-0.191	medium low	0.439	medium high	0.571	medium high
24117	Villaga	0.192	medium low	-0.743	low	0.236	medium low	0.376	medium low

Munic code	Comune	log_Ae. caspius	quartile class	log_Ae. vexans	quartile class	prob_Ae. caspius	quartile class	prob_Ae. vexans	quartile class
24118	Villaverla	-0.435	low	-0.737	low	0.296	medium high	0.551	medium high
30087	Ragogna	0.000	low	0.000	medium high	0.034	low	0.185	low
30056	Marano Lagunare	1.424	medium high	0.197	high	0.528	high	0.288	medium low
30020	Castions di Strada	0.427	medium low	0.143	high	0.401	medium high	0.613	high
30004	Aquileia	1.451	medium high	0.123	high	0.174	medium low	0.565	medium high
30057	Martignacco	-0.424	low	-0.141	medium high	0.016	low	0.266	low
31002	Cormons	-0.062	low	0.078	medium high	0.050	low	0.420	medium low
30069	Palazzolo dello Stella	1.120	medium high	0.350	high	0.556	high	0.634	high
30070	Palmanova	0.326	medium low	0.100	medium high	0.246	medium low	0.569	medium high
31013	Moraro	0.076	medium low	0.165	high	0.044	low	0.503	medium high
30049	Lignano Sabbiadoro	1.626	high	0.003	medium high	0.816	high	0.491	medium high
30126	Treppo Grande	0.000	low	0.000	medium high	0.015	low	0.204	low
30127	Tricesimo	0.000	low	0.000	medium high	0.023	low	0.207	low
31003	Doberdù del Lago	0.000	low	0.000	medium high	0.015	low	0.303	medium low
31005	Farra d'Isonzo	0.283	medium low	0.059	medium high	0.052	low	0.547	medium high
31006	Fogliano Redipuglia	0.832	medium high	0.191	high	0.038	low	0.329	medium low
30101	San Giovanni al Natisone	-0.175	low	-0.011	medium high	0.026	low	0.390	medium low
31012	Monfalcone	1.288	medium high	0.003	medium high	0.153	medium low	0.527	medium high
30128	Trivignano Udinese	0.049	medium low	0.011	medium high	0.066	low	0.434	medium high
30090	Reana del Rojale	-0.381	low	-0.025	medium high	0.038	low	0.260	low
30099	San Daniele del Friuli	-0.791	low	-0.071	medium high	0.061	low	0.301	medium low
30100	San Giorgio di Nogaro	0.937	medium high	0.227	high	0.404	medium high	0.604	high
30103	San Pietro al Natisone	-0.633	low	-0.073	medium high	0.002	low	0.063	low
30023	Cervignano del Friuli	0.964	medium high	0.184	high	0.286	medium high	0.634	high
30024	Chiopris-Viscone	0.121	medium low	0.076	medium high	0.083	medium low	0.510	medium high
30116	Tarcento	0.000	low	0.000	medium high	0.022	low	0.115	low
30118	Tavagnacco	-0.389	low	-0.135	medium high	0.020	low	0.274	medium low
30006	Artegna	0.000	low	0.000	medium high	0.029	low	0.216	low
30060	Moimacco	-0.696	low	-0.379	medium low	0.021	low	0.331	medium low
30052	Magnano in Riviera	0.000	low	0.000	medium high	0.026	low	0.168	low
30062	Mortegliano	0.048	medium low	-0.012	medium high	0.169	medium low	0.449	medium high
30063	Moruzzo	-0.423	low	-0.088	medium high	0.014	low	0.179	low
26031	Gaiarine	0.295	medium low	-0.237	medium low	0.098	medium low	0.511	medium high
26032	Giavera del Montello	0.370	medium low	-0.432	medium low	0.085	medium low	0.372	medium low

Munic code	Comune	log_Ae. caspius	quartile class	log_Ae. vexans	quartile class	prob_Ae. caspius	quartile class	prob_Ae. vexans	quartile class
30129	Udine	-0.391	low	-0.186	medium low	0.065	low	0.364	medium low
30130	Varmo	0.481	medium low	0.263	high	0.243	medium low	0.482	medium high
26022	Cordignano	0.226	medium low	-0.215	medium low	0.024	low	0.333	medium low
26026	Farra di Soligo	0.440	medium low	-0.384	medium low	0.019	low	0.193	low
31007	Gorizia	0.000	low	0.000	medium high	0.019	low	0.341	medium low
26033	Godega di Sant'Urbano	0.264	medium low	-0.084	medium high	0.052	low	0.461	medium high
24081	Pove del Grappa	-0.267	low	-0.590	low	0.064	low	0.041	low
24082	Pozzoleone	-0.080	low	-0.624	low	0.331	medium high	0.534	medium high
30091	Remanzacco	-0.557	low	-0.311	medium low	0.012	low	0.286	medium low
26069	Roncade	1.302	medium high	-0.324	medium low	0.144	medium low	0.707	high
26070	Salgareda	0.983	medium high	-0.141	medium high	0.148	medium low	0.613	high
31008	Gradisca d'Isonzo	0.458	medium low	0.192	high	0.076	low	0.561	medium high
30104	Santa Maria la Longa	0.105	medium low	-0.007	medium high	0.168	medium low	0.543	medium high
30105	San Vito al Torre	0.397	medium low	0.116	medium high	0.095	medium low	0.410	medium low
30106	San Vito di Fagagna	-0.534	low	-0.190	medium low	0.025	low	0.299	medium low
24083	Quinto Vicentino	0.530	medium low	-0.356	medium low	0.563	high	0.584	high
24120	Zermeghedo	0.186	medium low	-0.414	medium low	0.086	medium low	0.299	medium low
24122	Zugliano	-0.780	low	-0.863	low	0.142	medium low	0.295	medium low
30008	Bagnaria Arsa	0.641	medium high	0.173	high	0.276	medium high	0.666	high
30009	Basiliano	-0.364	low	-0.089	medium high	0.118	medium low	0.423	medium high
30053	Majano	0.000	low	0.000	medium high	0.048	low	0.304	medium low
24119	Zanè	-0.806	low	-0.837	low	0.028	low	0.248	low
30026	Cividale del Friuli	-0.723	low	-0.302	medium low	0.024	low	0.247	low
30131	Venzone	0.000	low	0.000	medium high	0.005	low	0.028	low
26027	Follina	0.426	medium low	0.000	medium high	0.025	low	0.126	low
30064	Muzzana del Turgnano	0.967	medium high	0.309	high	0.492	medium high	0.652	high
30134	Villa Vicentina	1.221	medium high	0.167	high	0.171	medium low	0.492	medium high
26028	Fontanelle	0.445	medium low	-0.149	medium high	0.052	low	0.488	medium high
26051	Oderzo	0.643	medium high	-0.159	medium high	0.188	medium low	0.622	high
30065	Nimis	0.000	low	0.000	medium high	0.010	low	0.088	low
30066	Osoppo	0.000	low	0.000	medium high	0.019	low	0.293	medium low
30095	Rive d'Arcano	-0.647	low	-0.211	medium low	0.038	low	0.250	low
30011	Bicinicco	0.195	medium low	0.030	medium high	0.271	medium high	0.521	medium high
30012	Bordano	0.000	low	0.000	medium high	0.005	low	0.020	low
26052	Ormelle	0.507	medium low	-0.249	medium low	0.074	low	0.527	medium high

Munic code	Comune	log_Ae. caspius	quartile class	log_Ae. vexans	quartile class	prob_Ae. caspius	quartile class	prob_Ae. vexans	quartile class
26053	Orsago	0.246	medium low	-0.179	medium low	0.061	low	0.500	medium high
26029	Fonte	0.363	medium low	0.062	medium high	0.214	medium low	0.423	medium high
31009	Grado	1.556	high	0.105	medium high	0.126	medium low	0.469	medium high
30013	Buja	0.000	low	0.000	medium high	0.024	low	0.247	low
30014	Buttrio	-0.424	low	-0.218	medium low	0.081	medium low	0.413	medium low
30055	Manzano	-0.317	low	-0.108	medium high	0.022	low	0.324	medium low
30135	Visco	0.431	medium low	0.123	high	0.237	medium low	0.648	high
30028	Colloredo di Monte Albano	0.000	low	0.000	medium high	0.036	low	0.252	low
30044	Gonars	0.431	medium low	0.131	high	0.360	medium high	0.633	high
30137	Forgaria nel Friuli	0.000	low	0.000	medium high	0.017	low	0.102	low
30138	Campolongo Tapogliano	0.709	medium high	0.158	high	0.155	medium low	0.501	medium high
31001	Capriva del Friuli	-0.056	low	0.131	high	0.081	medium low	0.517	medium high
26030	Fregona	0.155	medium low	0.000	medium high	0.007	low	0.025	low
26061	Possagno	0.241	medium low	-0.137	medium high	0.070	low	0.051	low
26062	Povegliano	0.340	medium low	-0.488	low	0.086	medium low	0.543	medium high
30030	Corno di Rosazzo	-0.396	low	-0.075	medium high	0.049	low	0.388	medium low
30046	Latisana	1.351	medium high	0.127	high	0.536	high	0.576	high
30123	Torviscosa	0.947	medium high	0.203	high	0.194	medium low	0.609	high
30097	Ronchis	0.862	medium high	0.334	high	0.295	medium high	0.479	medium high
30098	Ruda	0.995	medium high	0.173	high	0.174	medium low	0.552	medium high
30015	Camino al Tagliamento	0.103	medium low	0.188	high	0.196	medium low	0.385	medium low
26063	Preganziol	0.739	medium high	-0.218	medium low	0.156	medium low	0.603	high
24116	Vicenza	0.180	medium low	-0.418	medium low	0.398	medium high	0.481	medium high
30068	Pagnacco	-0.401	low	-0.065	medium high	0.009	low	0.192	low
31010	Mariano del Friuli	0.248	medium low	0.158	high	0.045	low	0.474	medium high
31011	Medea	0.218	medium low	0.121	high	0.082	medium low	0.454	medium high
30124	Trasaghis	0.000	low	0.000	medium high	0.006	low	0.027	low
30016	Campoformido	-0.365	low	-0.120	medium high	0.127	medium low	0.532	medium high
30018	Carlino	1.077	medium high	0.254	high	0.418	medium high	0.664	high
30019	Cassacco	0.000	low	0.000	medium high	0.011	low	0.206	low
34007	Busseto	0.045	medium low	0.912	high	0.041	low	0.521	medium high
33007	Cadeo	-0.237	low	0.119	high	0.086	medium low	0.445	medium high
32001	Duino-Aurisina	0.000	low	0.000	medium high	0.022	low	0.169	low
33041	San Pietro in Cerro	0.291	medium low	0.550	high	0.092	medium low	0.509	medium high

Munic code	Comune	log_Ae. caspius	quartile class	log_Ae. vexans	quartile class	prob_Ae. caspius	quartile class	prob_Ae. vexans	quartile class
33042	Sarmato	1.469	medium high	0.311	high	0.555	high	0.548	medium high
36001	Bastiglia	0.928	medium high	-0.526	low	0.263	medium high	0.566	medium high
33023	Gossolengo	0.223	medium low	0.318	high	0.287	medium high	0.349	medium low
33024	Gragnano Trebbiense	0.651	medium high	0.456	high	0.332	medium high	0.409	medium low
33046	Villanova sull'Arda	0.098	medium low	0.893	high	0.079	medium low	0.626	high
34018	Langhirano	-0.590	low	0.597	high	0.013	low	0.071	low
33045	Vigolzone	-0.186	low	-0.004	medium high	0.140	medium low	0.183	low
34019	Lesignano de' Bagni	-0.546	low	0.405	high	0.023	low	0.123	low
34024	Neviano degli Arduini	-0.226	low	0.146	high	0.008	low	0.069	low
34013	Felino	-0.717	low	0.732	high	0.020	low	0.157	low
97091	Verderio	0.191	medium low	-0.948	low	0.231	medium low	0.242	low
33001	Agazzano	0.778	medium high	0.221	high	0.329	medium high	0.268	low
36008	Castelvetro di Modena	0.100	medium low	0.157	high	0.075	low	0.187	low
33002	Alseno	-0.619	low	0.578	high	0.058	low	0.365	medium low
37005	Bentivoglio	1.200	medium high	0.663	high	0.116	medium low	0.655	high
33043	Travo	0.083	medium low	0.074	medium high	0.053	low	0.081	low
33048	Ziano Piacentino	1.240	medium high	0.000	medium high	0.286	medium high	0.203	low
33035	Podenzano	-0.099	low	0.039	medium high	0.203	medium low	0.317	medium low
33036	Ponte dell'Olio	-0.232	low	0.015	medium high	0.120	medium low	0.157	low
34020	Medesano	-0.807	low	1.044	high	0.013	low	0.211	low
34023	Montechiarugolo	-0.347	low	0.161	high	0.053	low	0.317	medium low
36009	Cavezzo	1.919	high	0.748	high	0.357	medium high	0.610	high
36010	Concordia sulla Secchia	1.817	high	1.132	high	0.408	medium high	0.583	high
34009	Collecchio	-0.785	low	0.866	high	0.014	low	0.259	low
34010	Colorno	0.505	medium low	1.410	high	0.105	medium low	0.660	high
34021	Mezzani	0.591	medium high	1.541	high	0.184	medium low	0.755	high
33021	Fiorenzuola d'Arda	-0.455	low	0.347	high	0.064	low	0.396	medium low
33003	Besenzone	-0.109	low	0.579	high	0.026	low	0.437	medium high
33022	Gazzola	0.356	medium low	0.323	high	0.270	medium high	0.223	low
34027	Parma	-0.391	low	0.684	high	0.045	low	0.398	medium low
33037	Pontenure	-0.054	low	0.159	high	0.097	medium low	0.359	medium low
33038	Rivergaro	0.047	medium low	0.145	high	0.183	medium low	0.198	low
33032	Piacenza	0.343	medium low	0.474	high	0.250	medium low	0.449	medium high
34014	Fidenza	-0.396	low	0.877	high	0.024	low	0.341	medium low
33018	Cortemaggiore	0.058	medium low	0.405	high	0.040	low	0.436	medium high
34015	Fontanellato	-0.111	low	0.937	high	0.016	low	0.421	medium high

Munic code	Comune	log_Ae. caspius	quartile class	log_Ae. vexans	quartile class	prob_Ae. caspius	quartile class	prob_Ae. vexans	quartile class
34016	Fontevivo	-0.390	low	0.909	high	0.020	low	0.405	medium low
31023	Staranzano	1.478	high	0.098	medium high	0.088	medium low	0.649	high
33039	Rottofreno	1.024	medium high	0.454	high	0.406	medium high	0.453	medium high
33040	San Giorgio Piacentino	-0.332	low	-0.009	medium high	0.136	medium low	0.274	medium low
33013	Castel San Giovanni	1.471	high	0.018	medium high	0.503	medium high	0.462	medium high
33014	Castelvetro Piacentino	0.125	medium low	0.887	high	0.132	medium low	0.642	high
34025	Noceto	-0.627	low	0.916	high	0.017	low	0.306	medium low
33027	Monticelli d'Ongina	0.448	medium low	0.732	high	0.183	medium low	0.667	high
33006	Borgonovo Val Tidone	1.116	medium high	0.155	high	0.422	medium high	0.366	medium low
33033	Pianello Val Tidone	0.935	medium high	0.051	medium high	0.173	medium low	0.106	low
37006	Bologna	0.504	medium low	0.225	high	0.041	low	0.385	medium low
36027	Nonantola	0.706	medium high	-0.587	low	0.235	medium low	0.560	medium high
36028	Novi di Modena	1.986	high	1.125	high	0.570	high	0.643	high
35043	Vezzano sul Crostolo	0.085	medium low	0.197	high	0.076	low	0.167	low
37011	Casalecchio di Reno	0.272	medium low	0.129	high	0.050	low	0.293	medium low
37003	Baricella	1.486	high	0.977	high	0.261	medium high	0.702	high
37008	Budrio	1.622	high	0.577	high	0.184	medium low	0.597	high
35033	Reggio nell'Emilia	0.459	medium low	0.354	high	0.135	medium low	0.449	medium high
35034	Rio Saliceto	1.604	high	0.918	high	0.553	high	0.608	high
35035	Rolo	1.942	high	1.405	high	0.655	high	0.623	high
36021	Medolla	1.835	high	0.701	high	0.371	medium high	0.643	high
36015	Formigine	0.117	medium low	0.262	high	0.125	medium low	0.327	medium low
36039	San Prospero	1.623	high	0.204	high	0.283	medium high	0.595	high
36044	Soliera	1.159	medium high	-0.264	medium low	0.307	medium high	0.572	medium high
36045	Spilamberto	0.064	medium low	0.045	medium high	0.084	medium low	0.347	medium low
36040	Sassuolo	0.101	medium low	0.483	high	0.114	medium low	0.203	low
36023	Modena	0.242	medium low	-0.362	medium low	0.265	medium high	0.477	medium high
36022	Mirandola	1.637	high	0.954	high	0.410	medium high	0.651	high
36007	Castelnuovo Rangone	0.123	medium low	0.075	medium high	0.125	medium low	0.349	medium low
37016	Castel Guelfo di Bologna	1.369	medium high	-0.108	medium high	0.065	low	0.465	medium high
35036	Rubiera	0.192	medium low	-0.004	medium high	0.314	medium high	0.523	medium high
28007	Arzergrande	1.169	medium high	-0.317	medium low	0.231	medium low	0.659	high
28057	Montegrotto Terme	0.360	medium low	-0.612	low	0.453	medium high	0.566	medium high
28058	Noventa Padovana	0.260	medium low	-0.672	low	0.621	high	0.559	medium high
36046	Vignola	0.072	medium low	0.099	medium high	0.091	medium low	0.320	medium low

Munic code	Comune	log_Ae. caspius	quartile class	log_Ae. vexans	quartile class	prob_Ae. caspius	quartile class	prob_Ae. vexans	quartile class
36002	Bomporto	1.291	medium high	-0.187	medium low	0.339	medium high	0.597	high
35029	Poviglio	0.759	medium high	1.431	high	0.143	medium low	0.552	medium high
35030	Quattro Castella	0.091	medium low	0.146	high	0.110	medium low	0.229	low
36005	Carpi	1.616	high	0.433	high	0.576	high	0.605	high
35037	San Martino in Rio	0.489	medium low	-0.020	medium high	0.234	medium low	0.509	medium high
35038	San Polo d'Enza	-0.170	low	0.063	medium high	0.063	low	0.197	low
28059	Ospedaletto Euganeo	0.115	medium low	-0.683	low	0.322	medium high	0.578	high
28101	Villa del Conte	0.027	medium low	-0.083	medium high	0.541	high	0.648	high
28102	Villa Estense	0.610	medium high	-0.661	low	0.470	medium high	0.666	high
36003	Campogalliano	0.396	medium low	-0.371	medium low	0.362	medium high	0.556	medium high
36004	Camposanto	1.680	high	0.352	high	0.326	medium high	0.649	high
28103	Villafranca Padovana	0.263	medium low	-0.316	medium low	0.507	high	0.570	medium high
29018	Costa di Rovigo	1.335	medium high	-0.371	medium low	0.367	medium high	0.712	high
29019	Crespino	2.227	high	1.018	high	0.689	high	0.776	high
36041	Savignano sul Panaro	0.067	medium low	0.029	medium high	0.086	medium low	0.265	low
29021	Ficarolo	1.469	medium high	0.195	high	0.616	high	0.819	high
29022	Fiesso Umbertiano	1.427	medium high	-0.119	medium high	0.495	medium high	0.791	high
28008	Bagnoli di Sopra	1.304	medium high	-0.323	medium low	0.099	medium low	0.566	medium high
36033	Prignano sulla Secchia	0.056	medium low	0.000	medium high	0.027	low	0.070	low
36034	Ravarino	1.231	medium high	-0.265	medium low	0.310	medium high	0.629	high
37017	Castello d'Argile	1.218	medium high	-0.038	medium high	0.114	medium low	0.599	high
28009	Baone	0.347	medium low	-0.665	low	0.313	medium high	0.375	medium low
28010	Barbona	0.951	medium high	-0.661	low	0.364	medium high	0.768	high
28011	Battaglia Terme	0.460	medium low	-0.616	low	0.611	high	0.650	high
28104	Villanova di Camposampiero	0.241	medium low	-0.368	medium low	0.381	medium high	0.566	medium high
37001	Anzola dell'Emilia	0.262	medium low	-0.015	medium high	0.100	medium low	0.492	medium high
37007	Borgo Tossignano	0.859	medium high	0.132	high	0.018	low	0.226	low
35039	Sant'Ilario d'Enza	0.079	medium low	0.268	high	0.091	medium low	0.443	medium high
35040	Scandiano	0.160	medium low	0.488	high	0.146	medium low	0.262	low
28060	Padova	0.186	medium low	-0.689	low	0.653	high	0.581	high
35032	Reggiolo	1.617	high	1.499	high	0.661	high	0.652	high
28105	Vo'	0.197	medium low	-0.638	low	0.360	medium high	0.314	medium low
28106	Due Carrare	0.491	medium low	-0.654	low	0.313	medium high	0.601	high
29003	Arquà Polesine	1.734	high	0.162	high	0.527	high	0.745	high
17026	Brandico	-0.216	low	-0.105	medium high	0.034	low	0.453	medium high
36012	Finale Emilia	1.564	high	0.754	high	0.330	medium high	0.653	high

Munic code	Comune	log_Ae. caspius	quartile class	log_Ae. vexans	quartile class	prob_Ae. caspius	quartile class	prob_Ae. vexans	quartile class
37045	Mordano	1.249	medium high	-0.284	medium low	0.111	medium low	0.589	high
37028	Galliera	1.388	medium high	0.440	high	0.157	medium low	0.694	high
20067	Villa Poma	0.991	medium high	0.370	high	0.446	medium high	0.595	high
38005	Codigoro	2.002	high	0.768	high	0.709	high	0.677	high
39004	Brisighella	0.470	medium low	-0.047	medium high	0.033	low	0.268	low
37021	Castenaso	1.058	medium high	0.225	high	0.088	medium low	0.464	medium high
37052	San Giorgio di Piano	1.209	medium high	0.431	high	0.109	medium low	0.573	high
38006	Comacchio	1.743	high	0.484	high	0.690	high	0.614	high
37048	Pieve di Cento	1.388	medium high	0.048	medium high	0.158	medium low	0.687	high
38007	Copparo	2.214	high	0.588	high	0.548	high	0.680	high
37032	Imola	1.239	medium high	-0.115	medium high	0.065	low	0.434	medium high
37035	Malalbergo	1.423	medium high	0.873	high	0.188	medium low	0.733	high
38001	Argenta	1.549	high	0.369	high	0.357	medium high	0.597	high
38002	Berra	2.125	high	1.118	high	0.682	high	0.745	high
37038	Minerbio	1.462	medium high	0.853	high	0.170	medium low	0.614	high
39002	Bagnacavallo	1.070	medium high	-0.090	medium high	0.082	medium low	0.488	medium high
40004	Borghi	-0.104	low	-0.038	medium high	0.007	low	0.222	low
40005	Castrocaro Terme e Terra del Sole	-0.046	low	-0.299	medium low	0.025	low	0.242	low
38014	Mesola	1.927	high	0.897	high	0.807	high	0.682	high
39009	Cotignola	0.795	medium high	-0.346	medium low	0.074	low	0.527	medium high
40045	Savignano sul Rubicone	0.056	medium low	-0.048	medium high	0.026	low	0.447	medium high
40016	Gatteo	0.114	medium low	-0.087	medium high	0.043	low	0.496	medium high
40018	Longiano	-0.051	low	-0.099	medium high	0.013	low	0.331	medium low
37056	Sant'Agata Bolognese	0.687	medium high	-0.492	low	0.249	medium low	0.549	medium high
39010	Faenza	0.374	medium low	-0.326	medium low	0.045	low	0.467	medium high
39011	Fusignano	1.273	medium high	0.041	medium high	0.154	medium low	0.486	medium high
38016	Mirabello	1.324	medium high	0.435	high	0.189	medium low	0.686	high
39003	Bagnara di Romagna	1.066	medium high	-0.285	medium low	0.084	medium low	0.562	medium high
33008	Calendasco	0.923	medium high	0.407	high	0.433	medium high	0.593	high
33010	Caorso	0.617	medium high	0.552	high	0.189	medium low	0.589	high
39017	Sant'Agata sul Santerno	1.160	medium high	-0.158	medium high	0.057	low	0.459	medium high
39018	Solarolo	0.872	medium high	-0.385	medium low	0.042	low	0.489	medium high
40028	Montiano	-0.143	low	0.000	medium high	0.009	low	0.264	low
38017	Ostellato	1.686	high	0.176	high	0.567	high	0.606	high
38022	Vigarano Mainarda	1.292	medium high	0.316	high	0.222	medium low	0.667	high
34017	Fornovo di Taro	-0.795	low	0.712	high	0.009	low	0.154	low
40012	Forlù	-0.126	low	-0.468	low	0.030	low	0.394	medium low

Munic code	Comune	log_Ae. caspius	quartile class	log_Ae. vexans	quartile class	prob_Ae. caspius	quartile class	prob_Ae. vexans	quartile class
38018	Poggio Renatico	1.353	medium high	0.420	high	0.152	medium low	0.619	high
38019	Portomaggiore	1.620	high	-0.124	medium high	0.486	medium high	0.642	high
33011	Carpaneto Piacentino	-0.490	low	0.116	medium high	0.136	medium low	0.309	medium low
39012	Lugo	1.159	medium high	-0.005	medium high	0.113	medium low	0.486	medium high
38023	Voghiera	1.649	high	-0.591	low	0.456	medium high	0.640	high
38024	Tresigallo	2.111	high	-0.031	medium high	0.539	high	0.643	high
38008	Ferrara	1.433	medium high	-0.110	medium high	0.439	medium high	0.741	high
38009	Formignana	2.176	high	0.074	medium high	0.581	high	0.686	high
38010	Jolanda di Savoia	2.434	high	0.789	high	0.680	high	0.718	high
31022	Savogna d'Isonzo	0.000	low	0.000	medium high	0.010	low	0.286	medium low
31024	Turriaco	1.204	medium high	0.174	high	0.120	medium low	0.654	high
40041	San Mauro Pascoli	0.102	medium low	-0.021	medium high	0.025	low	0.452	medium high
40003	Bertinoro	-0.169	low	-0.353	medium low	0.019	low	0.301	medium low
31025	Villesse	0.809	medium high	0.184	high	0.158	medium low	0.658	high
33012	Castell'Arquato	-0.641	low	0.294	high	0.078	low	0.272	medium low
40015	Gambettola	0.014	low	-0.113	medium high	0.037	low	0.440	medium high
38011	Lagosanto	1.773	high	0.583	high	0.862	high	0.664	high
38012	Masi Torello	1.878	high	-0.695	low	0.550	high	0.719	high
39006	Castel Bolognese	0.810	medium high	-0.301	medium low	0.053	low	0.416	medium low
38025	Goro	1.796	high	0.745	high	0.822	high	0.616	high
40013	Forlimpopoli	-0.220	low	-0.413	medium low	0.025	low	0.437	medium high
40007	Cesena	-0.017	low	-0.252	medium low	0.021	low	0.362	medium low
40008	Cesenatico	0.265	medium low	-0.155	medium high	0.064	low	0.495	medium high
39016	Russi	0.595	medium high	-0.413	medium low	0.138	medium low	0.513	medium high
39001	Alfonsine	1.488	high	0.134	high	0.254	medium high	0.504	medium high
39007	Cervia	0.553	medium low	-0.362	medium low	0.076	low	0.494	medium high
39008	Conselice	1.298	medium high	0.447	high	0.215	medium low	0.553	medium high
38020	Ro	2.106	high	0.687	high	0.454	medium high	0.745	high
38021	Sant'Agostino	1.381	medium high	0.352	high	0.157	medium low	0.667	high
39015	Riolo Terme	0.706	medium high	-0.088	medium high	0.027	low	0.261	low
39013	Massa Lombarda	1.292	medium high	-0.016	medium high	0.151	medium low	0.558	medium high
39014	Ravenna	1.065	medium high	-0.091	medium high	0.205	medium low	0.510	medium high
37054	San Lazzaro di Savena	0.830	medium high	0.068	medium high	0.029	low	0.306	medium low
38004	Cento	1.473	high	0.181	high	0.190	medium low	0.686	high
37055	San Pietro in Casale	1.386	medium high	0.500	high	0.135	medium low	0.605	high
37042	Monte San Pietro	0.083	medium low	0.059	medium high	0.032	low	0.188	low

Munic code	Comune	log_Ae. caspius	quartile class	log_Ae. vexans	quartile class	prob_Ae. caspius	quartile class	prob_Ae. vexans	quartile class
30058	Mereto di Tomba	-0.476	low	-0.139	medium high	0.031	low	0.306	medium low
41065	Tavullia	0.000	low	0.000	medium high	0.003	low	0.248	low
96051	Quaregna	1.677	high	0.072	medium high	0.187	medium low	0.115	low
96029	Lessona	1.655	high	0.049	medium high	0.426	medium high	0.186	low
96012	Candelo	1.725	high	-0.153	medium high	0.468	medium high	0.215	low
96032	Masserano	1.620	high	0.033	medium high	0.424	medium high	0.251	low
96035	Mongrando	1.685	high	-0.124	medium high	0.158	medium low	0.143	low
96020	Cossato	1.674	high	0.059	medium high	0.491	medium high	0.212	low
96004	Biella	1.681	high	-0.162	medium high	0.232	medium low	0.066	low
96037	Mottalciata	1.694	high	-0.023	medium high	0.743	high	0.332	medium low
96018	Cerrione	1.817	high	-0.115	medium high	0.386	medium high	0.233	low
96038	Muzzano	1.647	high	-0.155	medium high	0.127	medium low	0.031	low
96007	Brusnengo	1.603	high	0.034	medium high	0.565	high	0.251	low
96010	Camburzano	1.655	high	-0.114	medium high	0.211	medium low	0.075	low
96041	Occhieppo Superiore	1.649	high	-0.158	medium high	0.305	medium high	0.042	low
96031	Massazza	1.770	high	-0.168	medium high	0.560	high	0.316	medium low
96047	Ponderano	1.712	high	-0.147	medium high	0.140	medium low	0.160	low
96025	Dorzano	1.814	high	-0.070	medium high	0.743	high	0.297	medium low
96006	Borriana	1.737	high	-0.107	medium high	0.079	medium low	0.207	low
96040	Occhieppo Inferiore	1.671	high	-0.151	medium high	0.398	medium high	0.144	low
93052	Vajont	-1.249	low	-0.510	low	0.006	low	0.132	low
108030	Meda	0.459	medium low	-0.696	low	0.529	high	0.228	low
108031	Mezzago	0.303	medium low	-0.986	low	0.384	medium high	0.243	low
108032	Misinto	0.394	medium low	-0.398	medium low	0.531	high	0.269	medium low
93034	Prata di Pordenone	0.306	medium low	-0.325	medium low	0.057	low	0.410	medium low
93035	Pravisdomini	0.664	medium high	0.041	medium high	0.223	medium low	0.534	medium high
93036	Roveredo in Piano	-0.520	low	-0.579	low	0.045	low	0.310	medium low
108039	Seregno	0.649	medium high	-0.936	low	0.293	medium high	0.222	low
108040	Seveso	0.634	medium high	-0.639	low	0.670	high	0.267	low
108015	Carate Brianza	0.431	medium low	-1.060	low	0.459	medium high	0.247	low
108016	Carnate	0.514	medium low	-0.975	low	0.417	medium high	0.248	low
108017	Cavenago di Brianza	0.577	medium high	-1.056	low	0.549	high	0.351	medium low
108018	Ceriano Laghetto	0.665	medium high	-0.571	low	0.651	high	0.300	medium low
108041	Sovico	0.767	medium high	-0.945	low	0.572	high	0.249	low

Munic code	Comune	log_Ae. caspius	quartile class	log_Ae. vexans	quartile class	prob_Ae. caspius	quartile class	prob_Ae. vexans	quartile class
108042	Sulbiate	0.371	medium low	-0.985	low	0.513	high	0.280	medium low
30188	Rivignano Teor	0.624	medium high	0.279	high	0.355	medium high	0.554	medium high
108043	Triuggio	0.599	medium high	-1.022	low	0.502	medium high	0.254	low
108044	Usmate Velate	0.634	medium high	-0.977	low	0.524	high	0.277	medium low
108045	Varedo	1.100	medium high	-0.708	low	0.652	high	0.319	medium low
108046	Vedano al Lambro	1.216	medium high	-0.956	low	0.520	high	0.304	medium low
108048	Verano Brianza	0.223	medium low	-1.030	low	0.461	medium high	0.226	low
108049	Villasanta	1.189	medium high	-1.009	low	0.494	medium high	0.317	medium low
108050	Vimercate	0.767	medium high	-1.026	low	0.414	medium high	0.320	medium low
108051	Busnago	0.234	medium low	-0.994	low	0.464	medium high	0.308	medium low
108052	Caponago	0.879	medium high	-1.104	low	0.364	medium high	0.353	medium low
108053	Cornate d'Adda	0.127	medium low	-0.949	low	0.310	medium high	0.303	medium low
108054	Lentate sul Seveso	0.276	medium low	-0.523	low	0.619	high	0.240	low
108055	Roncello	0.313	medium low	-1.018	low	0.429	medium high	0.302	medium low
108034	Muggiù	1.287	medium high	-0.750	low	0.434	medium high	0.327	medium low
108035	Nova Milanese	1.231	medium high	-0.806	low	0.526	high	0.322	medium low
108036	Ornago	0.516	medium low	-1.031	low	0.496	medium high	0.334	medium low
108038	Ronco Briantino	0.308	medium low	-0.956	low	0.199	medium low	0.237	low
37061	Valsamoggia	0.062	medium low	-0.007	medium high	0.045	low	0.234	low
34049	Sissa Trecasali	0.202	medium low	1.270	high	0.045	low	0.540	medium high
108033	Monza	1.329	medium high	-0.881	low	0.571	high	0.335	medium low
96026	Gaglianico	1.727	high	-0.158	medium high	0.267	medium high	0.148	low
96027	Gifflenga	1.682	high	-0.039	medium high	0.755	high	0.355	medium low
99028	Poggio Torriana	-0.101	low	-0.034	medium high	0.009	low	0.310	medium low
96003	Benna	1.758	high	-0.165	medium high	0.509	high	0.229	low
38027	Fiscaglia	1.908	high	0.425	high	0.570	high	0.649	high
20071	Borgo Virgilio	1.121	medium high	0.998	high	0.647	high	0.729	high
93022	Fontanafredda	-0.138	low	-0.532	low	0.077	low	0.412	medium low
93043	Sesto al Reghena	0.429	medium low	0.092	medium high	0.290	medium high	0.554	medium high
93033	Pordenone	-0.250	low	-0.449	medium low	0.128	medium low	0.401	medium low
93029	Pasiano di Pordenone	0.421	medium low	-0.238	medium low	0.097	medium low	0.419	medium low
93037	Sacile	0.155	medium low	-0.409	medium low	0.032	low	0.406	medium low
93038	San Giorgio della Richinvelda	-1.089	low	-0.321	medium low	0.077	low	0.280	medium low
93044	Spilimbergo	-1.091	low	-0.515	low	0.041	low	0.270	medium low

Munic code	Comune	log_Ae. caspius	quartile class	log_Ae. vexans	quartile class	prob_Ae. caspius	quartile class	prob_Ae. vexans	quartile class
93039	San Martino al Tagliamento	-0.889	low	-0.183	medium low	0.125	medium low	0.367	medium low
93031	Polcenigo	-0.165	low	-0.569	low	0.024	low	0.118	low
93032	Porcia	-0.086	low	-0.468	low	0.107	medium low	0.438	medium high
93025	Maniago	-1.257	low	-0.432	medium low	0.010	low	0.105	low
93026	Meduno	0.000	low	0.000	medium high	0.001	low	0.030	low
93047	Travesio	0.000	low	0.000	medium high	0.010	low	0.086	low
93027	Montereale Valcellina	-1.221	low	-0.652	low	0.012	low	0.087	low
93028	Morsano al Tagliamento	0.592	medium high	0.246	high	0.279	medium high	0.491	medium high
93030	Pinzano al Tagliamento	0.000	low	0.000	medium high	0.050	low	0.292	medium low
93050	Vivaro	-1.225	low	-0.562	low	0.037	low	0.225	low
93040	San Quirino	-0.917	low	-0.578	low	0.045	low	0.282	medium low
93041	San Vito al Tagliamento	0.059	medium low	0.107	medium high	0.256	medium high	0.527	medium high
93051	Zoppola	-0.578	low	-0.212	medium low	0.146	medium low	0.424	medium high
93042	Sequals	-1.184	low	-0.109	medium high	0.016	low	0.199	low
108009	Biassono	1.001	medium high	-0.954	low	0.475	medium high	0.299	medium low
108010	Bovisio-Masciago	0.953	medium high	-0.647	low	0.633	high	0.288	medium low
108011	Briosco	0.095	medium low	-1.014	low	0.310	medium high	0.212	low
108012	Brugherio	1.287	medium high	-0.952	low	0.516	high	0.354	medium low
108013	Burago di Molgora	0.753	medium high	-1.064	low	0.561	high	0.367	medium low
108019	Cesano Maderno	0.800	medium high	-0.648	low	0.681	high	0.281	medium low
108020	Cogliate	0.537	medium low	-0.501	low	0.678	high	0.292	medium low
108021	Concorezzo	1.107	medium high	-1.060	low	0.427	medium high	0.300	medium low
108003	Albiate	0.635	medium high	-0.982	low	0.395	medium high	0.261	low
108004	Arcore	0.932	medium high	-1.019	low	0.463	medium high	0.311	medium low
108005	Barlassina	0.509	medium low	-0.629	low	0.602	high	0.218	low
108006	Bellusco	0.476	medium low	-1.013	low	0.515	high	0.265	low
108007	Bernareggio	0.410	medium low	-0.974	low	0.536	high	0.244	low
108023	Desio	1.009	medium high	-0.813	low	0.455	medium high	0.298	medium low
108024	Giussano	0.128	medium low	-1.020	low	0.393	medium high	0.212	low
108025	Lazzate	0.228	medium low	-0.395	medium low	0.586	high	0.239	low
108026	Lesmo	0.884	medium high	-0.962	low	0.489	medium high	0.265	low
108027	Limbiate	0.989	medium high	-0.598	low	0.712	high	0.308	medium low
108028	Lissone	1.098	medium high	-0.874	low	0.432	medium high	0.266	low
108001	Agrate Brianza	0.982	medium high	-1.083	low	0.524	high	0.342	medium low
108002	Aicurzio	0.376	medium low	-0.980	low	0.685	high	0.301	medium low

Munic code	Comune	log_Ae. caspius	quartile class	log_Ae. vexans	quartile class	prob_Ae. caspius	quartile class	prob_Ae. vexans	quartile class
30031	Coseano	-0.639	low	-0.257	medium low	0.029	low	0.285	medium low
30039	Flaibano	-0.723	low	-0.258	medium low	0.057	low	0.293	medium low
30109	Sedegliano	-0.562	low	-0.141	medium high	0.060	low	0.326	medium low
30027	Codroipo	-0.097	low	0.057	medium high	0.230	medium low	0.432	medium high
93053	Valvasone Arzene	-0.677	low	-0.086	medium high	0.107	medium low	0.349	medium low
108029	Macherio	0.886	medium high	-0.947	low	0.517	high	0.302	medium low
30048	Lestizza	-0.079	low	-0.008	medium high	0.065	low	0.364	medium low
30010	Bertiolo	0.180	medium low	0.104	medium high	0.241	medium low	0.500	medium high
30032	Dignano	-0.852	low	-0.406	medium low	0.037	low	0.296	medium low
96058	Salussola	1.805	high	-0.148	medium high	0.515	high	0.291	medium low
99016	San Clemente	0.000	low	0.000	medium high	0.005	low	0.256	low
99014	Rimini	-0.057	low	-0.083	medium high	0.017	low	0.422	medium high
96079	Villanova Biellese	1.741	high	-0.098	medium high	0.451	medium high	0.359	medium low
98041	Mulazzano	1.265	medium high	-0.931	low	0.599	high	0.540	medium high
98042	Orio Litta	1.494	high	0.179	high	0.790	high	0.674	high
99017	San Giovanni in Marignano	0.000	low	0.000	medium high	0.004	low	0.387	medium low
99003	Coriano	-0.196	low	-0.013	medium high	0.007	low	0.352	medium low
99011	Morciano di Romagna	0.000	low	0.000	medium high	0.004	low	0.340	medium low
99018	Santarcangelo di Romagna	-0.025	low	0.044	medium high	0.012	low	0.389	medium low
96059	Sandigliano	1.761	high	-0.143	medium high	0.332	medium high	0.206	low
99013	Riccione	-0.252	low	-0.363	medium low	0.010	low	0.353	medium low
96082	Zubiena	0.000	low	0.000	medium high	0.056	low	0.073	low
96076	Verrone	1.785	high	-0.153	medium high	0.647	high	0.269	medium low
99005	Misano Adriatico	-0.276	low	0.000	medium high	0.004	low	0.368	medium low
99020	Verucchio	-0.115	low	-0.053	medium high	0.008	low	0.258	low
96077	Vigliano Biellese	1.690	high	-0.134	medium high	0.467	medium high	0.165	low
96071	Valdengo	1.709	high	0.034	medium high	0.410	medium high	0.203	low
98058	Turano Lodigiano	1.124	medium high	-0.090	medium high	0.471	medium high	0.651	high
98034	Mairago	1.263	medium high	-0.164	medium high	0.364	medium high	0.587	high
98022	Corno Giovine	0.759	medium high	0.871	high	0.297	medium high	0.656	high
98035	Maleo	0.834	medium high	0.753	high	0.193	medium low	0.573	medium high
98036	Marudo	1.829	high	-0.637	low	0.689	high	0.477	medium high
98037	Massalengo	1.543	high	-0.465	low	0.435	medium high	0.516	medium high
98038	Meleti	0.732	medium high	0.895	high	0.221	medium low	0.631	high

Munic code	Comune	log_Ae. caspius	quartile class	log_Ae. vexans	quartile class	prob_Ae. caspius	quartile class	prob_Ae. vexans	quartile class
97020	Cernusco Lombardone	0.217	medium low	-0.897	low	0.346	medium high	0.159	low
97044	Lomagna	0.449	medium low	-0.960	low	0.260	medium high	0.209	low
98003	Boffalora d'Adda	1.009	medium high	-0.714	low	0.707	high	0.640	high
97061	Osnago	0.291	medium low	-0.948	low	0.221	medium low	0.183	low
97062	Paderno d'Adda	0.101	medium low	-0.923	low	0.213	medium low	0.228	low
98004	Borghetto Lodigiano	1.738	high	-0.245	medium low	0.502	medium high	0.606	high
98005	Borgo San Giovanni	1.651	high	-0.617	low	0.606	high	0.515	medium high
98006	Brembio	1.454	medium high	-0.058	medium high	0.304	medium high	0.544	medium high
98019	Codogno	0.986	medium high	0.443	high	0.215	medium low	0.518	medium high
98020	Comazzo	0.710	medium high	-1.084	low	0.654	high	0.565	medium high
98021	Cornegliano Laudense	1.412	medium high	-0.545	low	0.439	medium high	0.555	medium high
98023	Cornovecchio	0.739	medium high	0.925	high	0.235	medium low	0.634	high
98024	Corte Palasio	0.975	medium high	-0.354	medium low	0.563	high	0.650	high
98039	Merlino	0.806	medium high	-1.030	low	0.714	high	0.543	medium high
98040	Montanaso Lombardo	1.225	medium high	-0.716	low	0.747	high	0.608	high
98025	Crespiatica	0.829	medium high	-0.462	low	0.353	medium high	0.550	medium high
98026	Fombio	0.892	medium high	0.554	high	0.498	medium high	0.697	high
97048	Merate	0.168	medium low	-0.813	low	0.531	high	0.224	low
98043	Ospedaletto Lodigiano	1.348	medium high	0.167	high	0.484	medium high	0.576	high
98044	Ossago Lodigiano	1.513	high	-0.281	medium low	0.305	medium high	0.550	medium high
98059	Valera Fratta	1.890	high	-0.660	low	0.652	high	0.466	medium high
98007	Camairago	0.905	medium high	0.378	high	0.422	medium high	0.713	high
98008	Casaletto Lodigiano	1.725	high	-0.823	low	0.621	high	0.476	medium high
98009	Casalmaiocco	1.411	medium high	-0.989	low	0.819	high	0.520	medium high
98045	Pieve Fissiraga	1.614	high	-0.545	low	0.308	medium high	0.490	medium high
98046	Salerano sul Lambro	1.687	high	-0.731	low	0.718	high	0.544	medium high
98047	San Fiorano	0.831	medium high	0.611	high	0.258	medium high	0.577	high
98048	San Martino in Strada	1.357	medium high	-0.380	medium low	0.531	high	0.598	high
98027	Galgagnano	1.144	medium high	-0.762	low	0.647	high	0.609	high
98028	Graffignana	1.856	high	-0.389	medium low	0.618	high	0.576	high
98029	Guardamiglio	0.801	medium high	0.512	high	0.435	medium high	0.678	high
98060	Villanova del Sillaro	1.707	high	-0.434	medium low	0.455	medium high	0.573	medium high
98061	Zelo Buon Persico	1.019	medium high	-0.939	low	0.715	high	0.590	high
98010	Casalpusterlengo	1.168	medium high	0.133	high	0.449	medium high	0.582	high

Munic code	Comune	log_Ae. caspius	quartile class	log_Ae. vexans	quartile class	prob_Ae. caspius	quartile class	prob_Ae. vexans	quartile class
98049	San Rocco al Porto	0.583	medium high	0.619	high	0.348	medium high	0.673	high
98050	Sant'Angelo Lodigiano	1.864	high	-0.527	low	0.602	high	0.563	medium high
98030	Livraga	1.580	high	-0.002	medium high	0.465	medium high	0.620	high
98051	Santo Stefano Lodigiano	0.715	medium high	0.753	high	0.367	medium high	0.655	high
98011	Caselle Landi	0.718	medium high	0.852	high	0.298	medium high	0.739	high
98012	Caselle Lurani	1.798	high	-0.707	low	0.794	high	0.525	medium high
98013	Castelnuovo Bocca d'Adda	0.735	medium high	0.772	high	0.242	medium low	0.738	high
98014	Castiglione d'Adda	0.985	medium high	0.122	high	0.446	medium high	0.648	high
98052	Secugnago	1.304	medium high	-0.060	medium high	0.183	medium low	0.508	medium high
98053	Senna Lodigiana	1.229	medium high	0.348	high	0.509	high	0.689	high
98054	Somaglia	1.053	medium high	0.388	high	0.479	medium high	0.636	high
99001	Bellaria-Igea Marina	0.156	medium low	-0.051	medium high	0.044	low	0.458	medium high
98031	Lodi	1.190	medium high	-0.547	low	0.608	high	0.600	high
98032	Lodi Vecchio	1.543	high	-0.711	low	0.542	high	0.481	medium high
98033	Maccastorna	0.633	medium high	0.802	high	0.239	medium low	0.707	high
99002	Cattolica	0.000	low	0.000	medium high	0.006	low	0.399	medium low
97071	Robbiate	0.161	medium low	-0.903	low	0.335	medium high	0.230	low
98001	Abbadia Cerreto	0.856	medium high	-0.286	medium low	0.504	high	0.664	high
98055	Sordio	1.507	high	-0.960	low	0.612	high	0.507	medium high
98056	Tavazzano con Villavesco	1.425	medium high	-0.820	low	0.610	high	0.541	medium high
98002	Bertonico	1.039	medium high	-0.038	medium high	0.509	high	0.683	high
98015	Castiraga Vidardo	1.753	high	-0.630	low	0.772	high	0.555	medium high
98016	Cavacurta	0.901	medium high	0.459	high	0.221	medium low	0.590	high
98017	Cavenago d'Adda	1.152	medium high	-0.254	medium low	0.592	high	0.660	high
98018	Cervignano d'Adda	1.149	medium high	-0.922	low	0.582	high	0.515	medium high
98057	Terranova dei Passerini	1.051	medium high	0.166	high	0.408	medium high	0.635	high

Figure S1. Descriptives (graphics) of the 223 CO2 traps used for geostatistical analysis of *Ae. capisus* (traps activated in the 3 years with at least 1 female and an observation's number between 11 and 28).

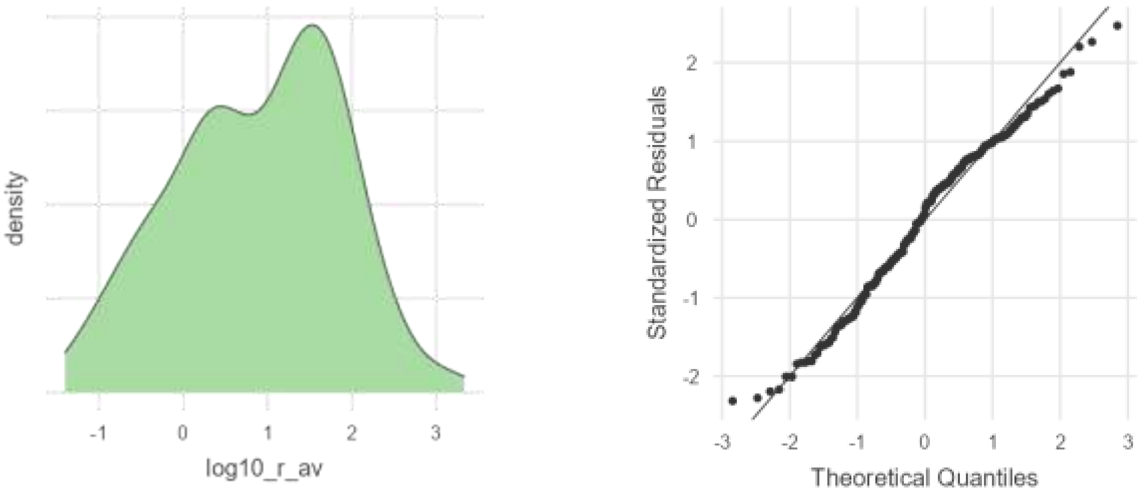

Figure S2. Descriptive (table and graphics) of the 205 CO2 traps used for geostatistical analysis of *Ae. vexans* (traps activated in the 3 years with at least 1 female and an observation's number between 11 and 28).

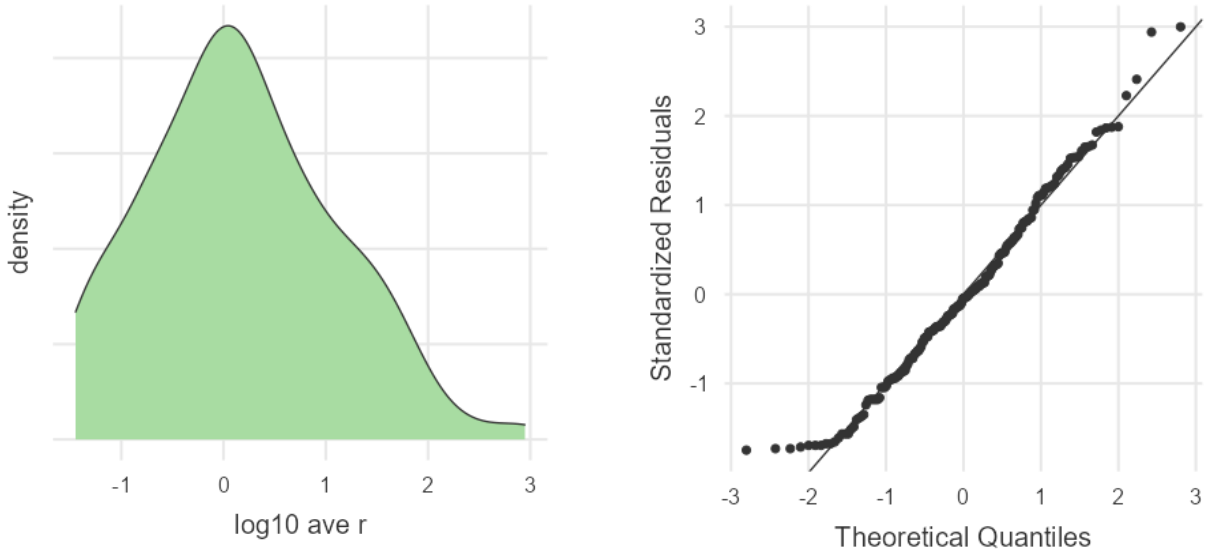

Figure S3. Curves showing how more relevant covariates affects the MaxEnt model of *Ae. caspius* and *Ae. vexans*. Name of the covariate over every graph.

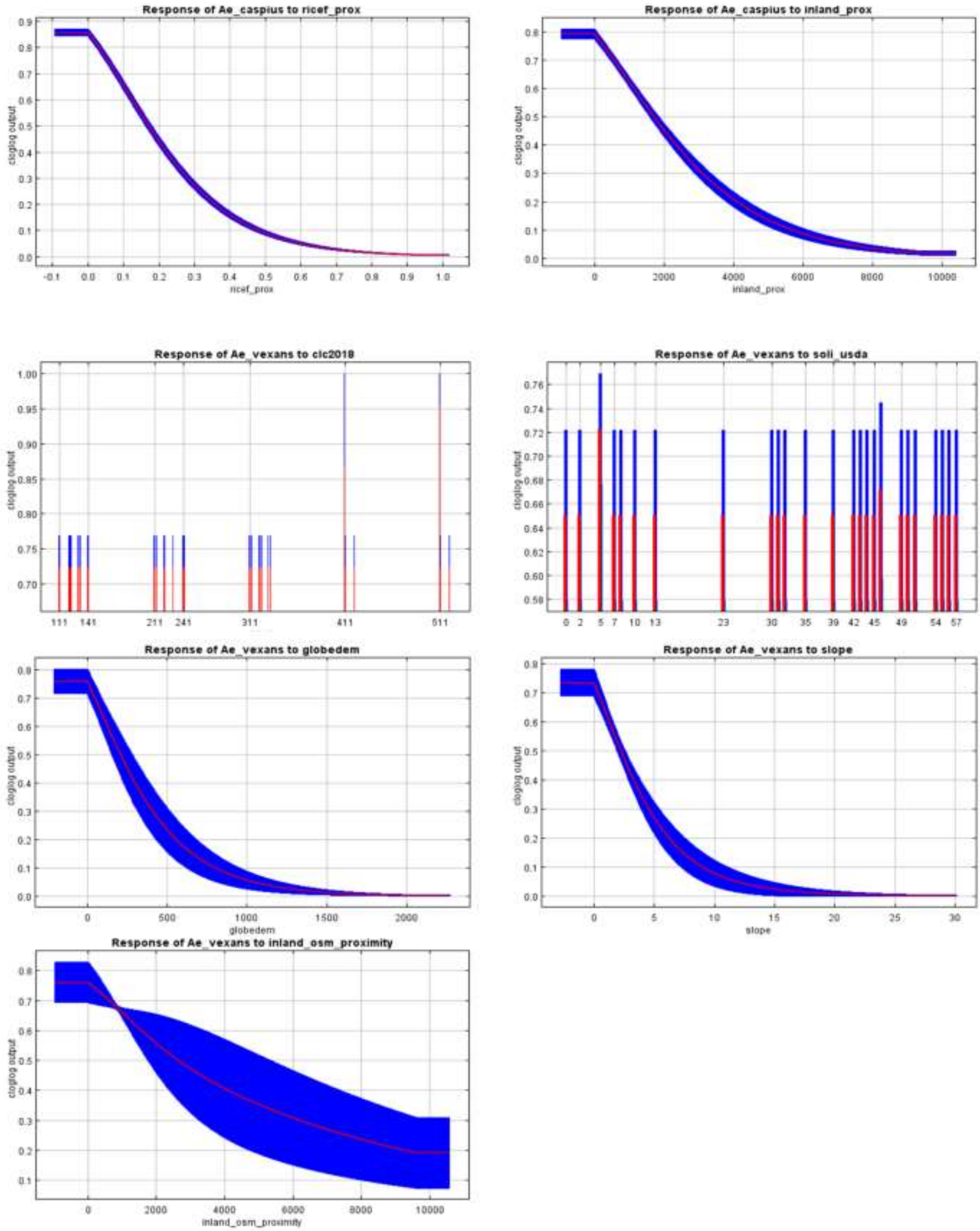

ù

Figure S4. Results of the jackknife test of variable importance using test gain.

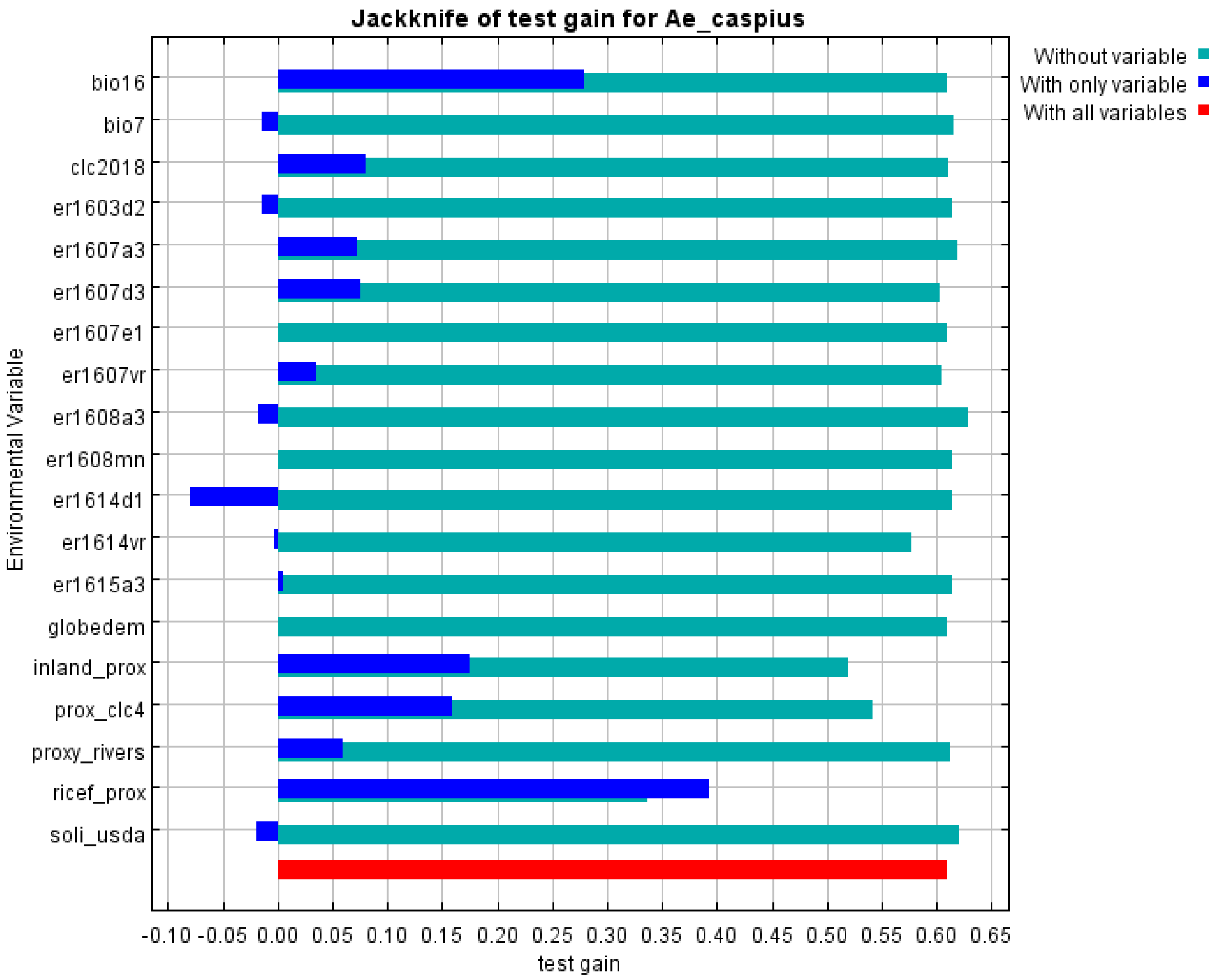

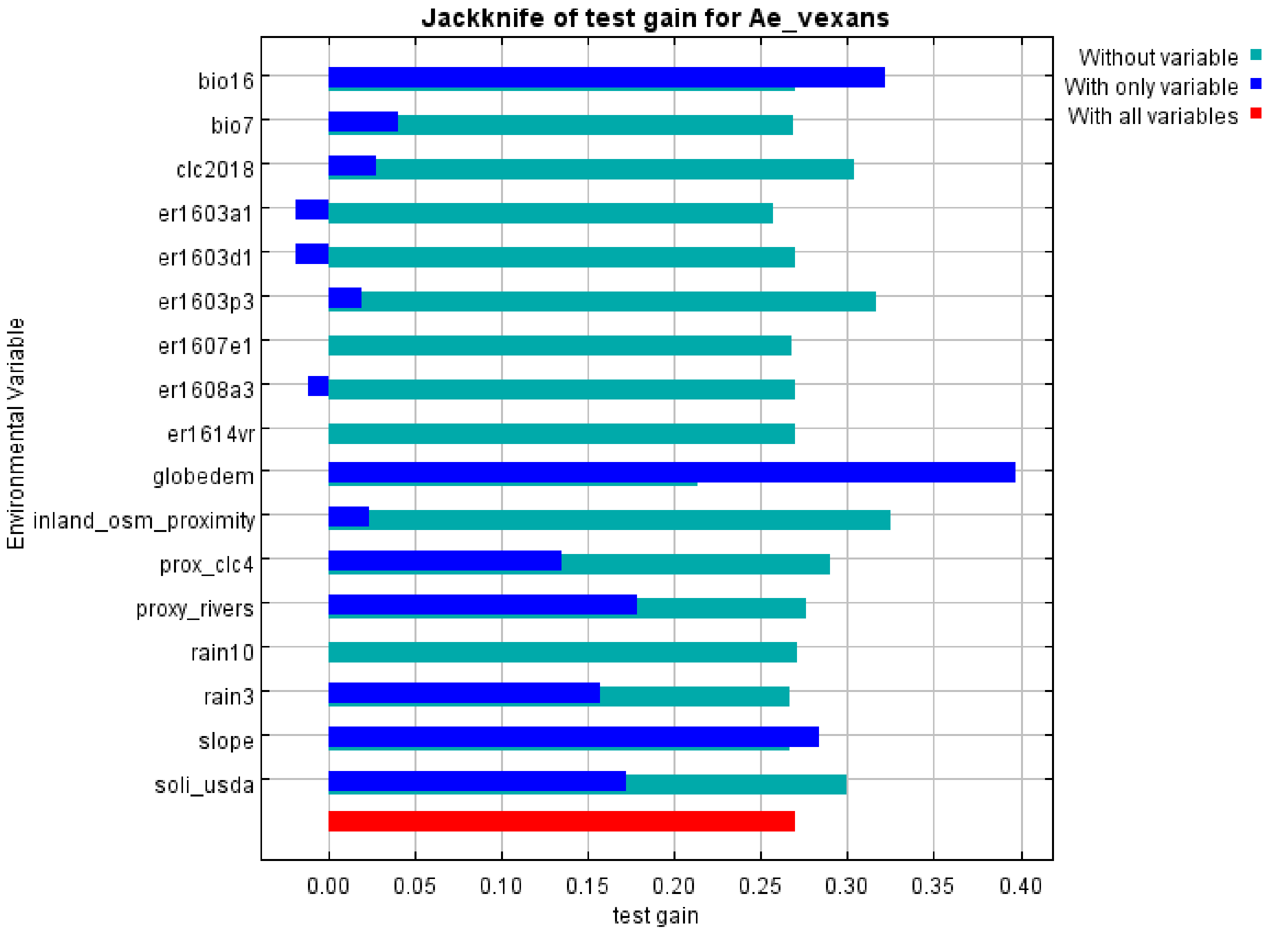
Jackknife of test gain for Ae_vexans
Without variable
With only variable
With all variables
Environmental Variable
bio16
bio7
clc2018
er1603a1
er1603d1
er1603p3
er1607e1
er1608a3
er1614vr
globedem
inland_osm_proximity
prox_clc4
proxy_rivers
rain10
rain3
slope
soli_usda
0.00
0.05
0.10
0.15
0.20
0.25
0.30
0.35
0.40
test gain

Figure S5. Choropleth map representing quartiles of ordinary kriging interpolation of *Ae. caspius* data at municipality level in the surveyed area.

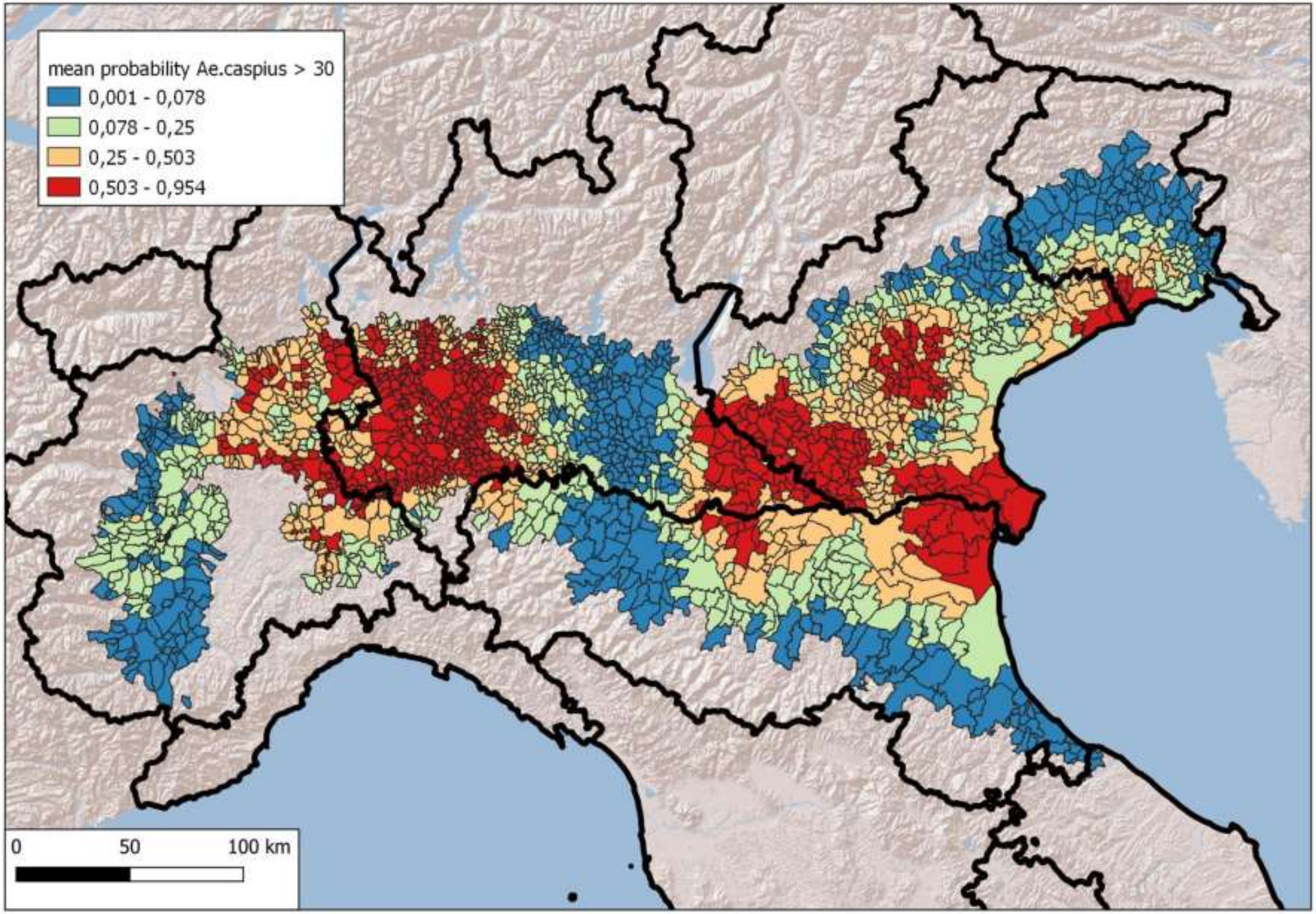

Figure S6. Choropleth map representing quartiles of Maxent model of *Ae. caspius* data at municipality level in the surveyed area.

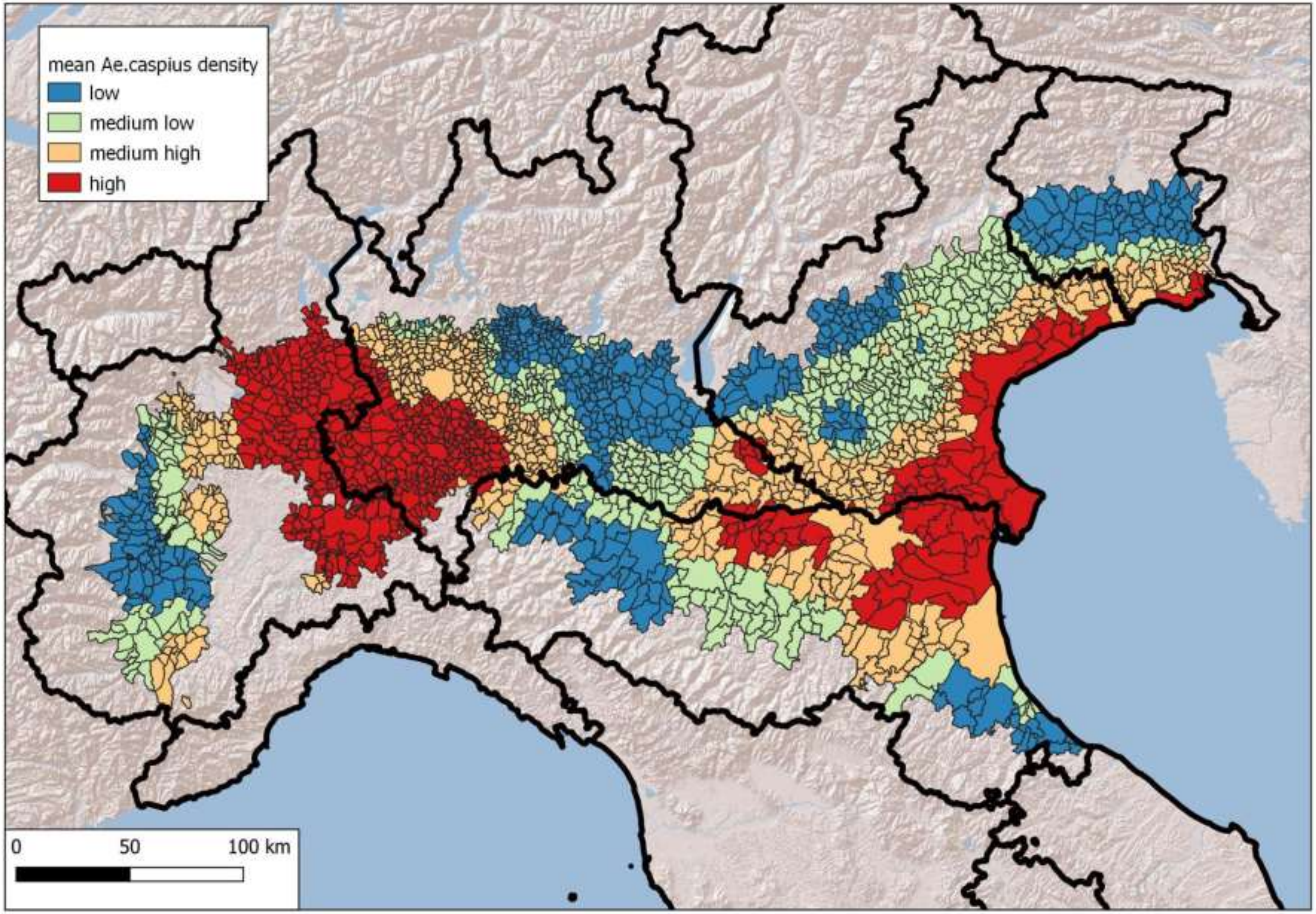

Figure S7. Choropleth map representing quartiles of ordinary kriging interpolation of *Ae. vexans* data at municipality level in the surveyed area.

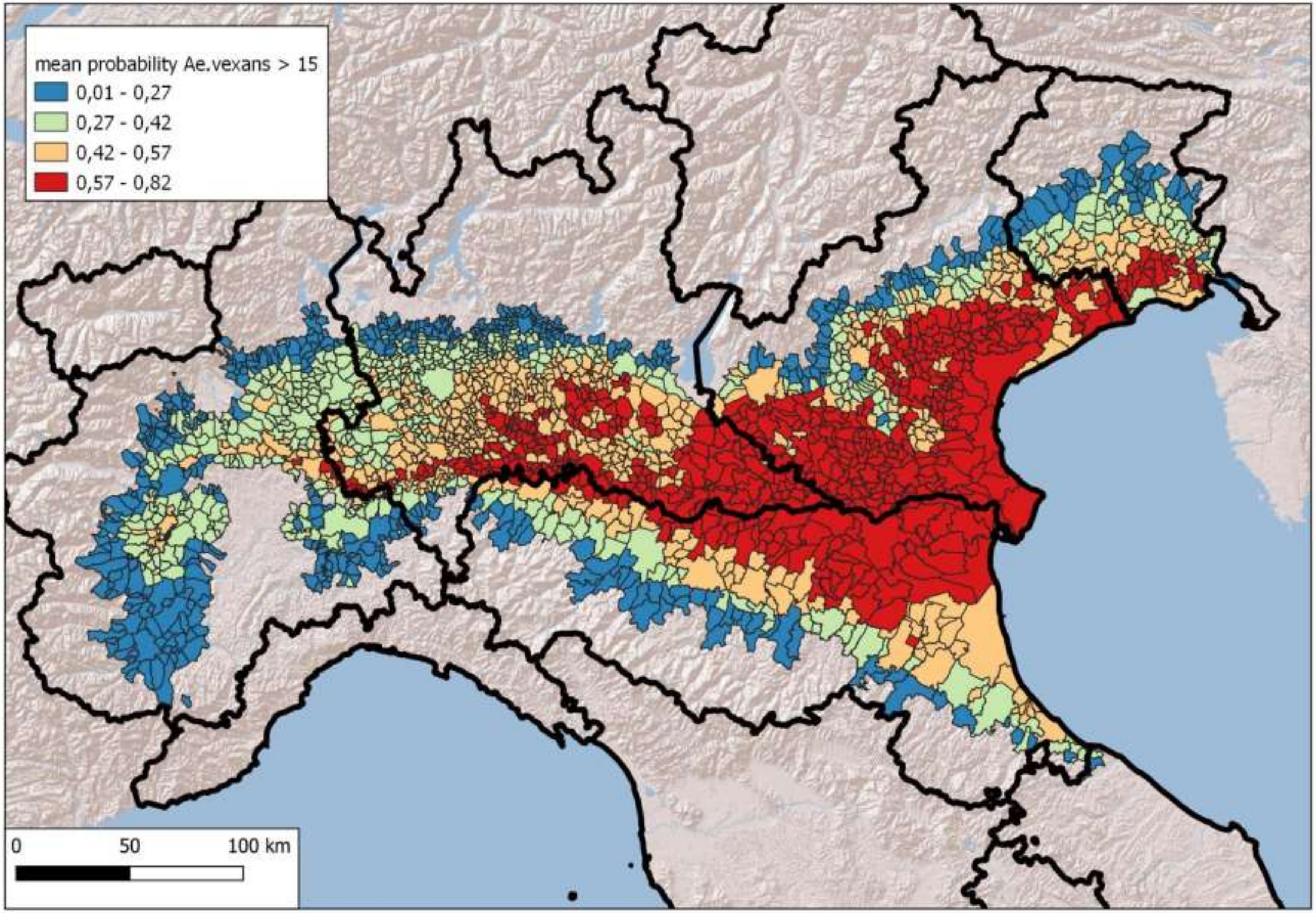

Figure S8. Choropleth map representing quartiles of Maxent model of *Ae. vexans* data at municipality level in the surveyed area.

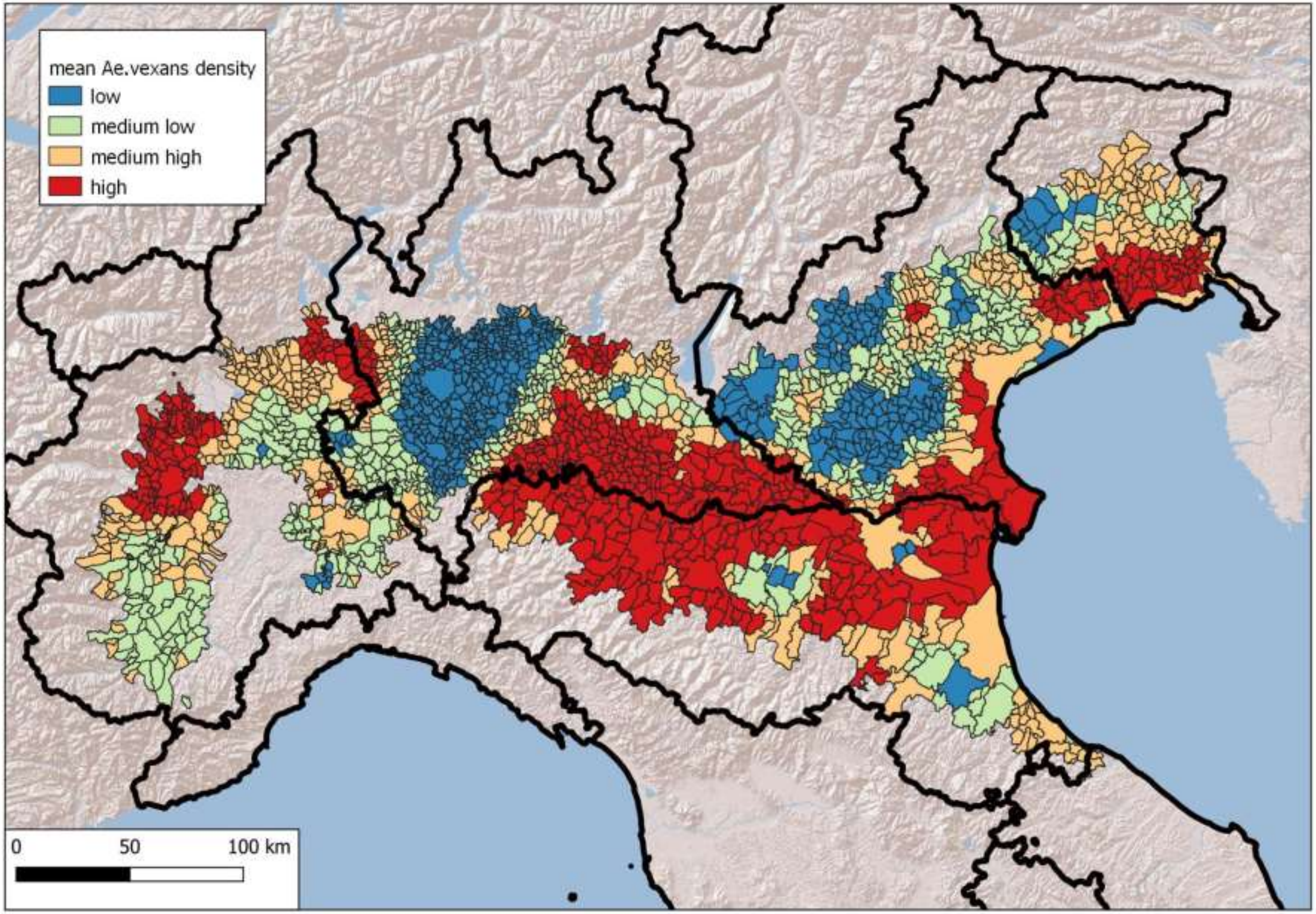

Supplement: Supplementary file 1 — Additional file 1 [file 13071_2024_6527_MOESM1_ESM.pdf]
